# Supplementary figures and images for: Combination of Hotspot Mutations With Methylation and Fragmentomic Profiles to Enhance Multi‐Cancer Early Detection
Source: Cancer Med. 2025 Jan 3;14(1):e70575. doi: 10.1002/cam4.70575 (PMC11695824; doi:10.1002/cam4.70575)

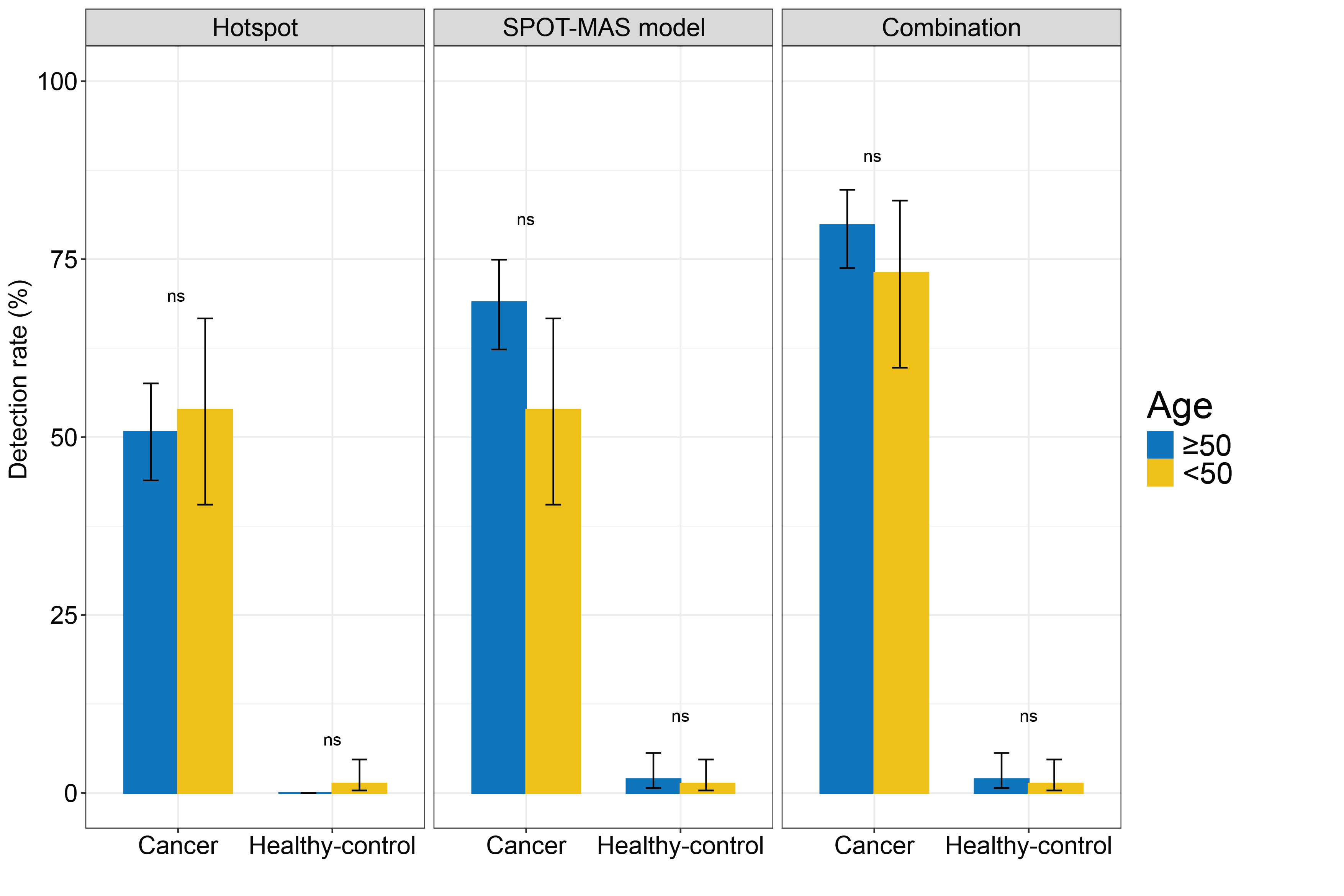

Supplement: Supplementary file 1 — Figure S1. Detection rates by (A) the Hotspot mutation assay, (B) the SPOT‐MAS assay, and (C) the combined Hotspot and SPOT‐MAS model, stratified by median age. [file CAM4-14-e70575-s003.png]
