# Supplementary material for: Combination of Hotspot Mutations With Methylation and Fragmentomic Profiles to Enhance Multi‐Cancer Early Detection
Source: Cancer Med. 2025 Jan 3;14(1):e70575. doi: 10.1002/cam4.70575 (PMC11695824; doi:10.1002/cam4.70575)
Supplement: Supplementary file 2 — Table S1. Clinical information of 559 participants. [file CAM4-14-e70575-s004.docx]

| **Table S1: Clinical information of 559 participants** | | | | | |  | |  |
| --- | --- | --- | --- | --- | --- | --- | --- | --- |
|  |  |  |  |  |  | |  | |
| **SampleID** | **roup** | **Age** | **GENDER** | **Stage** | **Diagnosis** | |  | |
| ZMC001 | Colorectal cancer | 48 | Male | II | Adenocarcinoma, moderately differentiation, infiltration | |  | |
| ZMC002 | Colorectal cancer | 34 | Female | II | Adenocarcinoma, moderately differentiation infiltration | |  | |
| ZMC004 | Colorectal cancer | 68 | Male | III | Adenocarcinoma, moderately differentiation, infiltration | |  | |
| ZMC005 | Colorectal cancer | 32 | Female | II | Adenocarcinoma, moderately differentiation, infiltration | |  | |
| ZMC006 | Colorectal cancer | 65 | Male | II | Adenocarcinoma, moderately differentiation, infiltration | |  | |
| ZMC008 | Colorectal cancer | 71 | Male | II | Adenocarcinoma, moderately differentiation, infiltration | |  | |
| ZMC009 | Colorectal cancer | 55 | Female | III | Adenocarcinoma moderately differentiation of colon, infiltration | |  | |
| ZMC016 | Colorectal cancer | 52 | Male | I | Adenocarcinoma moderately differentiation | |  | |
| ZMC029 | Colorectal cancer | 48 | Male | II | Adenocarcinoma moderately differentiation infiltration | |  | |
| ZMC037 | Colorectal cancer | 66 | Male | III | Adenocarcinoma moderately differentiationinfiltration | |  | |
| ZMC040 | Colorectal cancer | 73 | Female | III | Adenocarcinoma moderately differentiation infiltration | |  | |
| ZMC053 | Colorectal cancer | 72 | Female | II | Adenocarcinoma moderately differentiation infiltration | |  | |
| ZMC056 | Colorectal cancer | 80 | Male | II | Adenocarcinoma moderately differentiationinfiltration | |  | |
| ZMC060 | Colorectal cancer | 63 | Male | III | Adenocarcinoma moderately differentiation infiltration | |  | |
| ZMC065 | Colorectal cancer | 73 | Female | I | Adenocarcinoma moderately differentiation infiltration | |  | |
| ZMC069 | Colorectal cancer | 83 | Female | III | Adenocarcinoma moderately differentiation infiltration | |  | |
| ZMC074 | Colorectal cancer | 50 | Female | I | Adenocarcinoma moderately differentiation infiltration, tubular adenoma Haggitt 2 | |  | |
| ZMC075 | Colorectal cancer | 32 | Female | III | Mucinous adenocarcinoma, moderately differentiation infiltration | |  | |
| ZMC076 | Colorectal cancer | 56 | Male | II | Adenocarcinoma moderately differentiationinfiltration | |  | |
| ZMC119 | Colorectal cancer | 52 | Female | II | Adenocarcinoma, infiltration | |  | |
| ZMC124 | Colorectal cancer | 51 | Male | Nonmetastatic with unknow staging | Mucinous adenocarcinoma | |  | |
| ZMC082 | Colorectal cancer | 66 | Male | II | Adenocarcinoma, moderately differentiation | |  | |
| ZMC125 | Colorectal cancer | 63 | Female | III | Adenocarcinoma | |  | |
| YCAA33 | Colorectal cancer | 76 | Male | Nonmetastatic with unknow staging | Unknown | |  | |
| YCAA35 | Colorectal cancer | 75 | Male | Nonmetastatic with unknow staging | Adenocarcinoma moderately differentiation infiltration (Haggitt grade 3) | |  | |
| YCAB60 | Colorectal cancer | 67 | Male | Nonmetastatic with unknow staging | Unknown | |  | |
| ZMC072 | Colorectal cancer | 52 | Female | II | Adenocarcinoma moderately differentiation infiltration | |  | |
| ZMC077 | Colorectal cancer | 44 | Male | III | Adenocarcinoma, infiltration | |  | |
| ZMC123 | Colorectal cancer | 56 | Male | II | Adenocarcinoma, infiltration | |  | |
| ZMH002 | Liver cancer | 53 | Male | III | Trabecular hepatocellular carcinoma, moderately differentiation | |  | |
| ZMH004 | Liver cancer | 51 | Male | III | Trabecular hepatocellular carcinoma, poorly differentiation | |  | |
| ZMH005 | Liver cancer | 68 | Female | I | Clear cell hepatocellular carcinoma | |  | |
| ZMH006 | Liver cancer | 35 | Female | II | Trabecular hepatocellular carcinoma, poorly differentiation | |  | |
| ZMH008 | Liver cancer | 49 | Male | III | Trabecular hepatocellular carcinoma, poorly differentiation | |  | |
| ZMH010 | Liver cancer | 50 | Male | II | Hepatocellular carcinoma | |  | |
| ZMH011 | Liver cancer | 61 | Male | III | Sarcomatoid hepatocellular carcinoma | |  | |
| ZMH012 | Liver cancer | 59 | Male | III | Trabecular hepatocellular carcinoma, poorly differentiation | |  | |
| ZMH014 | Liver cancer | 64 | Male | I | Trabecular and pseudoacinar hepatocellular carcinoma, moderately differentiation | |  | |
| ZMH015 | Liver cancer | 59 | Male | I | Trabecular and pseudoacinar hepatocellular carcinoma, moderately differentiation | |  | |
| ZMH018 | Liver cancer | 71 | Male | II | Trabecular hepatocellular carcinoma | |  | |
| ZMH019 | Liver cancer | 62 | Male | I | Trabecular hepatocellular carcinoma, moderately differentiation | |  | |
| ZMH023 | Liver cancer | 52 | Male | II | Trabecular hepatocellular carcinoma, poorly differentiation | |  | |
| ZMH025 | Liver cancer | 68 | Male | I | Trabecular hepatocellular carcinoma, moderately differentiation | |  | |
| ZMH032 | Liver cancer | 63 | Male | III | Trabecular hepatocellular carcinoma, moderately differentiation | |  | |
| ZMH034 | Liver cancer | 46 | Male | I | Trabecular hepatocellular carcinoma, moderately differentiation | |  | |
| ZMH036 | Liver cancer | 39 | Female | II | Trabecular and clear cell hepatocellular carcinoma, moderately differentiation | |  | |
| ZMH039 | Liver cancer | 46 | Male | II | Trabecular hepatocellular carcinoma, poorly differentiation | |  | |
| ZMH043 | Liver cancer | 65 | Male | II | Trabecular and pseudoacinar hepatocellular carcinoma, moderately differentiation | |  | |
| ZMH044 | Liver cancer | 58 | Male | I | Trabecular hepatocellular carcinoma, poorly differentiation | |  | |
| ZMH048 | Liver cancer | 67 | Male | II | Trabecular hepatocellular carcinoma, moderately differentiation | |  | |
| ZMH050 | Liver cancer | 44 | Male | III | Trabecular hepatocellular carcinoma, poorly differentiation | |  | |
| ZMH052 | Liver cancer | 70 | Male | I | Trabecular hepatocellular carcinoma, moderately differentiation | |  | |
| ZMH053 | Liver cancer | 59 | Female | I | Trabecular hepatocellular carcinoma, moderately differentiation | |  | |
| ZMH054 | Liver cancer | 47 | Male | II | Trabecular hepatocellular carcinoma, poorly differentiation | |  | |
| ZMH059 | Liver cancer | 70 | Male | II | Trabecular hepatocellular carcinoma, moderately differentiation | |  | |
| ZMH060 | Liver cancer | 59 | Male | II | Hepatocellular carcinoma, poorly differentiation | |  | |
| ZMH062 | Liver cancer | 32 | Male | III | Trabecular and clear cell hepatocellular carcinoma, moderately differentiation | |  | |
| ZMG086 | Gastric cancer | 48 | Male | Nonmetastatic with unknow staging | Unknown | |  | |
| ZMG005 | Gastric cancer | 78 | Male | III | Poorly differentiation | |  | |
| ZMG010 | Gastric cancer | 76 | Female | Nonmetastatic with unknow staging | Unknown | |  | |
| ZMG011 | Gastric cancer | 74 | Female | II | Moderately differentiation | |  | |
| ZMG013 | Gastric cancer | 58 | Female | II | Signet ring cell | |  | |
| ZMG019 | Gastric cancer | 59 | Male | III | Moderately differentiation | |  | |
| ZMG021 | Gastric cancer | 78 | Male | Nonmetastatic with unknow staging | Unknown | |  | |
| ZMG024 | Gastric cancer | 69 | Male | I | Moderately differentiation | |  | |
| ZMG027 | Gastric cancer | 55 | Female | III | Moderately differentiation | |  | |
| ZMG028 | Gastric cancer | 67 | Male | III | Poorly differentiation | |  | |
| ZMG029 | Gastric cancer | 58 | Female | II | Poorly differentiation | |  | |
| ZMG032 | Gastric cancer | 47 | Male | II | Poorly differentiation | |  | |
| ZMG038 | Gastric cancer | 65 | Male | II | Poorly differentiation | |  | |
| ZMG042 | Gastric cancer | 50 | Male | II | Poorly differentiation | |  | |
| ZMG045 | Gastric cancer | 48 | Male | III | Poorly differentiation | |  | |
| ZMG046 | Gastric cancer | 58 | Male | III | Moderately differentiation | |  | |
| ZMG047 | Gastric cancer | 60 | Male | II | Moderately differentiation | |  | |
| ZMG048 | Gastric cancer | 75 | Male | II | Moderately differentiation | |  | |
| ZMG049 | Gastric cancer | 57 | Female | Nonmetastatic with unknow staging | Unknown | |  | |
| ZMG054 | Gastric cancer | 61 | Male | III | Moderately differentiation | |  | |
| ZMG061 | Gastric cancer | 53 | Female | Nonmetastatic with unknow staging | Unknown | |  | |
| ZMG062 | Gastric cancer | 67 | Female | Nonmetastatic with unknow staging | Unknown | |  | |
| ZMG069 | Gastric cancer | 65 | Female | III | Poorly differentiation | |  | |
| ZMG076 | Gastric cancer | 67 | Male | Nonmetastatic with unknow staging | Unknown | |  | |
| ZMG088 | Gastric cancer | 60 | Female | III | Poorly differentiation | |  | |
| ZMG097 | Gastric cancer | 54 | Female | II | Signet ring cell | |  | |
| ZMG099 | Gastric cancer | 61 | Female | III | Undifferentiated differentiation | |  | |
| ZMG115 | Gastric cancer | 68 | Male | III | Moderately differentiation | |  | |
| ZMG117 | Gastric cancer | 65 | Male | Nonmetastatic with unknow staging | Unknown | |  | |
| ZMG119 | Gastric cancer | 78 | Male | II | Poorly differentiation | |  | |
| ZMG123 | Gastric cancer | 76 | Female | I | Moderately differentiation | |  | |
| ZMG124 | Gastric cancer | 67 | Female | I | Signet ring cell | |  | |
| ZMG126 | Gastric cancer | 70 | Male | III | Undifferentiated differentiation | |  | |
| ZMG127 | Gastric cancer | 73 | Male | II | Moderately differentiation | |  | |
| ZMG089 | Gastric cancer | 84 | Female | Nonmetastatic with unknow staging | Unknown | |  | |
| ZMG091 | Gastric cancer | 51 | Male | III | Moderately differentiation | |  | |
| ZMG131 | Gastric cancer | 77 | Female | III | Moderately differentiation | |  | |
| ZMG132 | Gastric cancer | 66 | Male | II | Unknown | |  | |
| ZMG129 | Gastric cancer | 56 | Female | I | Unknown | |  | |
| ZMG142 | Gastric cancer | 53 | Male | II | Poorly differentiation | |  | |
| ZMG146 | Gastric cancer | 64 | Female | Nonmetastatic with unknow staging | Unknown | |  | |
| ZMG149 | Gastric cancer | 59 | Male | III | Moderately differentiation | |  | |
| ZMG151 | Gastric cancer | 61 | Female | Nonmetastatic with unknow staging | Unknown | |  | |
| ZMG154 | Gastric cancer | 59 | Male | III | Poorly differentiation | |  | |
| ZMG156 | Gastric cancer | 59 | Male | III | Moderately differentiation | |  | |
| ZMG157 | Gastric cancer | 38 | Male | III | Signet ring cell | |  | |
| ZMG159 | Gastric cancer | 55 | Male | III | Moderately differentiation | |  | |
| ZMG160 | Gastric cancer | 68 | Male | III | Undifferentiated differentiation | |  | |
| ZMB501 | Breast cancer | 59 | Female | II | Infiltrating ductal carcinoma, no-special type, grade 2 -Her-2 (+) | |  | |
| ZMB526 | Breast cancer | 54 | Female | II | Infiltrating ductal carcinoma, no-special type, grade 1, mucinous carcinoma -Luminal B | |  | |
| ZMB028 | Breast cancer | 64 | Female | II | Infiltrating ductal carcinoma, no-special type, grade 2, mucinous carcinoma -Luminal B | |  | |
| ZMB040 | Breast cancer | 50 | Female | II | Infiltrating ductal carcinoma, no-special type, grade 2, mucinous carcinoma -Triple Negative | |  | |
| ZMB134 | Breast cancer | 32 | Female | III | Infiltrating ductal carcinoma, no-special type, grade 2, mucinous carcinoma -Luminal B | |  | |
| ZMB135 | Breast cancer | 28 | Female | II | Infiltrating ductal carcinoma, no-special type, grade 2, mucinous carcinoma -Luminal B | |  | |
| ZMB138 | Breast cancer | 52 | Female | II | Infiltrating ductal carcinoma, no-special type, grade 3, mucinous carcinoma -Luminal B-Her2 | |  | |
| ZMB139 | Breast cancer | 43 | Female | II | Invasive lobular carcinoma, infiltration  -Luminal B-Her2 | |  | |
| ZMB145 | Breast cancer | 40 | Female | Nonmetastatic with unknow staging | Infiltrating ductal carcinoma, no-special type, grade 2 -Luminal A | |  | |
| ZMB146 | Breast cancer | 37 | Female | III | Infiltrating ductal carcinoma, no-special type, grade 2 -Luminal A | |  | |
| ZMB148 | Breast cancer | 67 | Female | I | Infiltrating ductal carcinoma, grade 1, mucinous carcinoma -Luminal B | |  | |
| ZMB149 | Breast cancer | 60 | Female | Nonmetastatic with unknow staging | Infiltrating ductal carcinoma, no-special type, grade 3, mucinous carcinoma -Triple Negative | |  | |
| ZMB152 | Breast cancer | 41 | Female | II | Intraductal Papillary carcinoma -Luminal A | |  | |
| ZMB153 | Breast cancer | 70 | Female | Nonmetastatic with unknow staging | Infiltrating ductal carcinoma, no-special type, grade 2 with Paget's disease -Her-2 (+) | |  | |
| ZMB154 | Breast cancer | 61 | Female | II | Infiltrating ductal carcinoma, no-special type, grade 2, mucinous carcinoma -Luminal B-Her2 | |  | |
| ZMB168 | Breast cancer | 67 | Female | I | Infiltrating ductal carcinoma, no-special type, grade 2, mucinous carcinoma -Luminal B | |  | |
| ZMB170 | Breast cancer | 42 | Female | II | Infiltrating ductal carcinoma, no-special type, grade 3, mucinous carcinoma -Luminal B | |  | |
| ZMB171 | Breast cancer | 48 | Female | I | Infiltrating ductal carcinoma, no-special type, grade 3 -Luminal B | |  | |
| ZMB178 | Breast cancer | 65 | Female | I | Adenocarcinoma -Luminal A | |  | |
| ZMB185 | Breast cancer | 56 | Female | III | Infiltrating ductal carcinoma, no-special type, grade 2 -Her-2 (+) | |  | |
| ZMB210 | Breast cancer | 38 | Female | III | Infiltrating ductal carcinoma, no-special type, grade 2 -Her-2 (+) | |  | |
| ZMB510 | Breast cancer | 72 | Female | II | Adenocarcinoma -Luminal B | |  | |
| ZMB512 | Breast cancer | 45 | Female | I | Infiltrating ductal carcinoma, no-special type, grade 3 -Triple Negative | |  | |
| ZMB522 | Breast cancer | 46 | Female | I | Infiltrating ductal carcinoma, no-special type, grade 1 -Luminal A | |  | |
| ZMB524 | Breast cancer | 75 | Female | II | Infiltrating ductal carcinoma, no-special type, grade 2 -Triple Negative | |  | |
| ZMB527 | Breast cancer | 59 | Female | I | Infiltrating ductal carcinoma, no-special type, grade 2 -Luminal B | |  | |
| ZMB528 | Breast cancer | 38 | Female | II | Infiltrating ductal carcinoma, no-special type, grade 2 -Luminal B | |  | |
| ZMB142 | Breast cancer | 43 | Female | II | Infiltrating ductal carcinoma, no-special type, grade 3 -Luminal B | |  | |
| ZMB163 | Breast cancer | 32 | Female | I | Infiltrating ductal carcinoma, no-special type, grade 3 -Luminal B | |  | |
| ZMB167 | Breast cancer | 68 | Female | Nonmetastatic with unknow staging | Infiltrating ductal carcinoma, no-special type, grade 3 | |  | |
| ZMB181 | Breast cancer | 48 | Female | II | Infiltrating ductal carcinoma, no-special type, grade 3 -Her-2 (+) | |  | |
| ZMB183 | Breast cancer | 45 | Female | II | Infiltrating ductal carcinoma, no-special type, grade 3 -Her-2 (+) | |  | |
| ZMB201 | Breast cancer | 51 | Female | II | Infiltrating ductal carcinoma, no-special type, grade 2 -Luminal B | |  | |
| ZMB536 | Breast cancer | 31 | Female | II | Infiltrating ductal carcinoma, no-special type, grade 2 -Triple Negative | |  | |
| ZMB537 | Breast cancer | 67 | Female | III | Infiltrating ductal carcinoma, no-special type, grade 2 -Luminal B | |  | |
| ZMB538 | Breast cancer | 63 | Female | II | Infiltrating ductal carcinoma, no-special type, grade 3 -Luminal B-Her2 | |  | |
| ZMB540 | Breast cancer | 46 | Female | II | Infiltrating ductal carcinoma, no-special type, grade 2 -Luminal B-Her2 | |  | |
| ZMB544 | Breast cancer | 52 | Female | II | Infiltrating ductal carcinoma, no-special type, grade 2 -Luminal B-Her2 | |  | |
| ZMB548 | Breast cancer | 49 | Female | I | Infiltrating ductal carcinoma, no-special type, grade 2 -Triple Negative | |  | |
| ZMB549 | Breast cancer | 56 | Female | II | Infiltrating ductal carcinoma, no-special type, grade 2 -Luminal B | |  | |
| ZMB551 | Breast cancer | 53 | Female | I | Adenocarcinoma infiltration -Luminal B | |  | |
| ZMB557 | Breast cancer | 43 | Female | II | Infiltrating ductal carcinoma, no-special type, grade 2 -Luminal A | |  | |
| ZMB559 | Breast cancer | 51 | Female | II | Infiltrating ductal carcinoma, no-special type, grade 1 -Luminal A | |  | |
| ZMB560 | Breast cancer | 45 | Female | II | Infiltrating ductal carcinoma, no-special type, grade 2 -Luminal A | |  | |
| ZMB561 | Breast cancer | 63 | Female | III | Invasive lobular carcinoma, infiltration  -Luminal B | |  | |
| ZMB567 | Breast cancer | 63 | Female | II | Infiltrating ductal carcinoma, no-special type, grade 2 -Triple Negative | |  | |
| ZMB569 | Breast cancer | 44 | Female | II | Infiltrating ductal carcinoma, no-special type, grade 2 -Luminal A | |  | |
| ZMB570 | Breast cancer | 55 | Female | II | Infiltrating ductal carcinoma, no-special type, grade 3 -Triple Negative | |  | |
| ZMB572 | Breast cancer | 50 | Female | II | Infiltrating ductal carcinoma, no-special type, grade 2 -Her-2 (+) | |  | |
| ZMB574 | Breast cancer | 63 | Female | II | Infiltrating ductal carcinoma, no-special type, grade 2 -Luminal B-Her2 | |  | |
| ZMB575 | Breast cancer | 61 | Female | II | Infiltrating ductal carcinoma, no-special type, grade 2 -Luminal B-Her2 | |  | |
| ZMB576 | Breast cancer | 58 | Female | II | Adenocarcinoma grade 2 -Luminal B-Her2 | |  | |
| ZMB584 | Breast cancer | 52 | Female | II | Infiltrating ductal carcinoma, no-special type, grade 2 | |  | |
| ZMB585 | Breast cancer | 42 | Female | II | Infiltrating ductal carcinoma, no-special type, grade 2 | |  | |
| ZMB590 | Breast cancer | 52 | Female | II | Infiltrating ductal carcinoma, no-special type, grade 2 -Luminal A | |  | |
| ZMB596 | Breast cancer | 63 | Female | II | Infiltrating ductal carcinoma, no-special type, grade 2 -Triple Negative | |  | |
| ZMB598 | Breast cancer | 65 | Female | II | Infiltrating ductal carcinoma, no-special type, grade 2 -Luminal A | |  | |
| ZMB601 | Breast cancer | 59 | Female | II | Infiltrating ductal carcinoma, no-special type, grade 3 -Triple Negative | |  | |
| ZMB605 | Breast cancer | 60 | Female | II | Mucinous carcinoma, no-special type, grade 2 -Luminal B | |  | |
| ZMB607 | Breast cancer | 53 | Female | II | Unknown | |  | |
| ZMB608 | Breast cancer | 36 | Female | II | Infiltrating ductal carcinoma, no-special type, grade 2 -Luminal B | |  | |
| ZMB609 | Breast cancer | 62 | Female | II | Infiltrating ductal carcinoma, no-special type, grade 2 -Luminal A | |  | |
| ZMB612 | Breast cancer | 51 | Female | II | Infiltrating ductal carcinoma, no-special type, grade 2 -Luminal B | |  | |
| ZMB007 | Breast cancer | 39 | Female | II | Infiltrating ductal carcinoma, no-special type, grade 2 -Luminal B-Her2 | |  | |
| ZMG164 | Gastric cancer | 70 | Female | II | Moderately differentiation | |  | |
| ZMG031 | Gastric cancer | 62 | Female | III | Moderately differentiation | |  | |
| ZMG150 | Gastric cancer | 63 | Female | III | Poorly differentiation | |  | |
| ZMH027 | Liver cancer | 32 | Male | II | Trabecular hepatocellular carcinoma, poorly differentiation | |  | |
| LAAF62 | Lung cancer | 66 | Male | III | Adenocarcinoma, poorly differentiation | |  | |
| LAAJ24 | Lung cancer | 51 | Male | III | Adenocarcinoma, poorly differentiation | |  | |
| LABF82 | Lung cancer | 56 | Male | III | Unknown | |  | |
| LABB48 | Lung cancer | 63 | Female | III | Adenocarcinoma | |  | |
| LABD46 | Lung cancer | 70 | Female | III | Unknown | |  | |
| LABD63 | Lung cancer | 76 | Male | III | Adenocarcinoma | |  | |
| LAAW21 | Lung cancer | 73 | Female | III | Unknown | |  | |
| YCAB67 | Lung cancer | 72 | Female | Nonmetastatic with unknow staging | Adenocarcinoma | |  | |
| LABH01 | Lung cancer | 69 | Female | III | Squamous cell carcinoma | |  | |
| LHAF44 | Lung cancer | 66 | Male | III | Unknown | |  | |
| YHAA03 | Lung cancer | 71 | Male | Nonmetastatic with unknow staging | Unknown | |  | |
| YHAA05 | Lung cancer | 69 | Female | Nonmetastatic with unknow staging | Unknown | |  | |
| L12866 | Lung cancer | 74 | Male | III | Adenocarcinoma, poorly differentiation | |  | |
| L12964 | Lung cancer | 60 | Male | III | Adenocarcinoma, well differentiation | |  | |
| NL09 | Lung cancer | 63 | Male | Nonmetastatic with unknow staging | Adenocarcinoma | |  | |
| NL11 | Lung cancer | 43 | Female | Nonmetastatic with unknow staging | Adenocarcinoma | |  | |
| NL27 | Lung cancer | 73 | Female | Nonmetastatic with unknow staging | Adenocarcinoma | |  | |
| LABA97 | Lung cancer | 65 | Female | III | Unknown | |  | |
| QHAA87 | Lung cancer | 34 | Male | III | Adenocarcinoma | |  | |
| LBG073 | Gastric cancer | NA | Female | Nonmetastatic with unknow staging | Unknown | |  | |
| LBG66 | Gastric cancer | 71 | Female | II | Moderately differentiation | |  | |
| LBG32 | Gastric cancer | 61 | Female | I | Well differentiation | |  | |
| LBG38 | Gastric cancer | 39 | Female | I | Moderately differentiation | |  | |
| LBG42 | Gastric cancer | 69 | Male | I | Moderately differentiation | |  | |
| LBG43 | Gastric cancer | 69 | Male | I | Moderately differentiation | |  | |
| LBG44 | Gastric cancer | 52 | Female | I | High grade dysplasia of the gastric mucosa | |  | |
| LBG45 | Gastric cancer | 70 | Male | I | Moderately differentiation | |  | |
| ZMG111 | Gastric cancer | 69 | Female | II | Unknown | |  | |
| LBG67 | Gastric cancer | 73 | Female | I | Well differentiation | |  | |
| LC002 | Colorectal cancer | 57 | Female | II | Unknown | |  | |
| LC007 | Colorectal cancer | 74 | Female | II | Adenocarcinoma | |  | |
| LC010 | Colorectal cancer | 71 | Female | II | Adenocarcinoma moderately differentiation | |  | |
| LC015 | Colorectal cancer | 52 | Female | Nonmetastatic with unknow staging | Unknown | |  | |
| LC018 | Colorectal cancer | 61 | Male | II | Adenocarcinoma | |  | |
| LC019 | Colorectal cancer | 32 | Female | Nonmetastatic with unknow staging | Adenocarcinoma moderately differentiation | |  | |
| LC021 | Colorectal cancer | NA | Female | Nonmetastatic with unknow staging | Adenocarcinoma moderately differentiation infiltration | |  | |
| LC023 | Colorectal cancer | 80 | Female | Nonmetastatic with unknow staging | Adenocarcinoma moderately differentiation, infiltration | |  | |
| LC029 | Colorectal cancer | 66 | Male | Nonmetastatic with unknow staging | Adenocarcinoma | |  | |
| LC034 | Colorectal cancer | 65 | Female | Nonmetastatic with unknow staging | Adenocarcinoma moderately differentiation | |  | |
| LC036 | Colorectal cancer | 66 | Male | Nonmetastatic with unknow staging | Adenocarcinoma moderately differentiation | |  | |
| LC038 | Colorectal cancer | 73 | Female | Nonmetastatic with unknow staging | Adenocarcinoma infiltration | |  | |
| LC043 | Colorectal cancer | 61 | Male | Nonmetastatic with unknow staging | Adenocarcinoma | |  | |
| LC046 | Colorectal cancer | 60 | Female | Nonmetastatic with unknow staging | Unknown | |  | |
| LC049 | Colorectal cancer | 61 | Male | Nonmetastatic with unknow staging | Unknown | |  | |
| LC069 | Colorectal cancer | 49 | Male | I | Adenocarcinoma | |  | |
| LC084 | Colorectal cancer | 70 | Male | Nonmetastatic with unknow staging | Adenocarcinoma moderately differentiation | |  | |
| LC091 | Colorectal cancer | 67 | Female | Nonmetastatic with unknow staging | Adenocarcinoma moderately differentiation | |  | |
| LC053 | Colorectal cancer | NA | Male | Nonmetastatic with unknow staging | Unknown | |  | |
| LBG30 | Gastric cancer | 42 | Male | I | Moderately differentiation | |  | |
| LC131 | Colorectal cancer | 70 | Female | III | Adenocarcinoma moderately differentiation, infiltration | |  | |
| LC133 | Colorectal cancer | 74 | Male | III | Adenocarcinoma | |  | |
| LC135 | Colorectal cancer | 68 | Female | II | Adenocarcinoma moderately differentiation, infiltration | |  | |
| LC136 | Colorectal cancer | NA | Male | Nonmetastatic with unknow staging | Unknown | |  | |
| LC139 | Colorectal cancer | 77 | Female | III | Unknown | |  | |
| LC143 | Colorectal cancer | 75 | Male | Nonmetastatic with unknow staging | Unknown | |  | |
| LABY23 | Colorectal cancer | 56 | Male | III | Adenocarcinoma | |  | |
| LABY25 | Colorectal cancer | 56 | Male | II | Adenocarcinoma | |  | |
| L12401 | Lung cancer | 62 | Female | III | Adenocarcinoma | |  | |
| L11979 | Lung cancer | 69 | Male | III | Adenocarcinoma | |  | |
| L12406 | Lung cancer | 57 | Male | III | Adenocarcinoma | |  | |
| L12174 | Lung cancer | 68 | Male | I | Adenocarcinoma, poorly differentiation | |  | |
| L12859 | Lung cancer | 79 | Male | II | Adenocarcinoma, poorly differentiation | |  | |
| L12880 | Lung cancer | 50 | Male | III | Adenocarcinoma | |  | |
| L12997 | Lung cancer | 68 | Female | III | Adenocarcinoma, poorly differentiation | |  | |
| L13200 | Lung cancer | 42 | Female | III | Adenocarcinoma | |  | |
| LAAD30 | Lung cancer | 71 | Male | III | Adenocarcinoma | |  | |
| ZMC093 | Colorectal cancer | 44 | Male | Nonmetastatic with unknow staging | Unknown | |  | |
| ZMC094 | Colorectal cancer | 60 | Male | Nonmetastatic with unknow staging | Unknown | |  | |
| ZMC211 | Colorectal cancer | 60 | Female | Nonmetastatic with unknow staging | Unknown | |  | |
| LABA02 | Lung cancer | 50 | Female | Nonmetastatic with unknow staging | Unknown | |  | |
| LABD60 | Lung cancer | 59 | Male | Nonmetastatic with unknow staging | Adenocarcinoma | |  | |
| LAAZ94 | Lung cancer | 76 | Male | Nonmetastatic with unknow staging | Unknown | |  | |
| LAAN29 | Lung cancer | 73 | Male | Nonmetastatic with unknow staging | Adenocarcinoma | |  | |
| LAAT20 | Lung cancer | 71 | Male | III | Adenocarcinoma | |  | |
| LABH01 | Lung cancer | 69 | Female | III | Squamous cell carcinoma | |  | |
| LAAL31 | Lung cancer | 65 | Male | Nonmetastatic with unknow staging | Unknown | |  | |
| LAAL98 | Lung cancer | 53 | Female | Nonmetastatic with unknow staging | Unknown | |  | |
| LAAM80 | Lung cancer | 68 | Male | Nonmetastatic with unknow staging | Unknown | |  | |
| LAAM90 | Lung cancer | 77 | Male | Nonmetastatic with unknow staging | Unknown | |  | |
| LAAK82 | Lung cancer | 54 | Male | Nonmetastatic with unknow staging | Adenocarcinoma | |  | |
| LAAM03 | Lung cancer | 63 | Male | Nonmetastatic with unknow staging | Unknown | |  | |
| LAAM12 | Lung cancer | 53 | Female | Nonmetastatic with unknow staging | Unknown | |  | |
| K0AAAN87 | Healthy-control | 58 | Male |  |  | |  | |
| K0AAAO67 | Healthy-control | 61 | Female |  |  | |  | |
| K0AAAO68 | Healthy-control | 60 | Male |  |  | |  | |
| K0AAAO69 | Healthy-control | 46 | Female |  |  | |  | |
| K0AAAO70 | Healthy-control | 47 | Female |  |  | |  | |
| K0AAAO71 | Healthy-control | 41 | Male |  |  | |  | |
| K0AAAO73 | Healthy-control | 48 | Male |  |  | |  | |
| K0AAAO76 | Healthy-control | 57 | Male |  |  | |  | |
| K0AAAO78 | Healthy-control | 49 | Female |  |  | |  | |
| K0AAAO79 | Healthy-control | 58 | Male |  |  | |  | |
| K0AAAO80 | Healthy-control | 52 | Male |  |  | |  | |
| K0AAAO81 | Healthy-control | 62 | Female |  |  | |  | |
| K0AAAO82 | Healthy-control | 43 | Female |  |  | |  | |
| K0AAAO84 | Healthy-control | 51 | Male |  |  | |  | |
| K0AAAO85 | Healthy-control | 45 | Female |  |  | |  | |
| K0AAAO86 | Healthy-control | 47 | Female |  |  | |  | |
| K0AAAO87 | Healthy-control | 48 | Male |  |  | |  | |
| K0AAAO91 | Healthy-control | 40 | Male |  |  | |  | |
| K0AAAO92 | Healthy-control | 46 | Male |  |  | |  | |
| K0AAAO93 | Healthy-control | 64 | Male |  |  | |  | |
| K0AAAO95 | Healthy-control | 50 | Female |  |  | |  | |
| K0AAAO97 | Healthy-control | 68 | Male |  |  | |  | |
| K0AAAP02 | Healthy-control | 44 | Male |  |  | |  | |
| K0AAAP03 | Healthy-control | 60 | Female |  |  | |  | |
| K0AAAP07 | Healthy-control | 62 | Male |  |  | |  | |
| K0AAAP09 | Healthy-control | 43 | Female |  |  | |  | |
| K0AAAP11 | Healthy-control | 61 | Female |  |  | |  | |
| K0AAAP13 | Healthy-control | 40 | Male |  |  | |  | |
| K0AAAP15 | Healthy-control | 49 | Male |  |  | |  | |
| K0AAAP16 | Healthy-control | 50 | Male |  |  | |  | |
| K0AAAP20 | Healthy-control | 42 | Male |  |  | |  | |
| K0AAAP27 | Healthy-control | 51 | Male |  |  | |  | |
| K0AAAP29 | Healthy-control | 58 | Female |  |  | |  | |
| K0AAAP31 | Healthy-control | 44 | Female |  |  | |  | |
| K0AAAP32 | Healthy-control | 56 | Female |  |  | |  | |
| K0AAAP33 | Healthy-control | 44 | Male |  |  | |  | |
| K0AAAP35 | Healthy-control | 53 | Male |  |  | |  | |
| K0AAAP36 | Healthy-control | 52 | Female |  |  | |  | |
| K0AAAP37 | Healthy-control | 50 | Female |  |  | |  | |
| K0AAAP39 | Healthy-control | 65 | Male |  |  | |  | |
| K0AAAP43 | Healthy-control | 46 | Female |  |  | |  | |
| K0AAAP44 | Healthy-control | 79 | Female |  |  | |  | |
| K0AAAP46 | Healthy-control | 48 | Male |  |  | |  | |
| K0AAAP48 | Healthy-control | 51 | Female |  |  | |  | |
| K0AAAP49 | Healthy-control | 43 | Female |  |  | |  | |
| K0AAAP50 | Healthy-control | 45 | Female |  |  | |  | |
| K0AAAP53 | Healthy-control | 52 | Male |  |  | |  | |
| K0AAAP58 | Healthy-control | 52 | Female |  |  | |  | |
| K0AAAP64 | Healthy-control | 44 | Male |  |  | |  | |
| K0AAAP67 | Healthy-control | 66 | Male |  |  | |  | |
| K0AAAP68 | Healthy-control | 57 | Female |  |  | |  | |
| K0AAAP69 | Healthy-control | 74 | Female |  |  | |  | |
| K0AAAP70 | Healthy-control | 42 | Male |  |  | |  | |
| K0AAAP71 | Healthy-control | 60 | Female |  |  | |  | |
| K0AAAP72 | Healthy-control | 45 | Male |  |  | |  | |
| K0AAAP73 | Healthy-control | 41 | Female |  |  | |  | |
| K0AAAP78 | Healthy-control | 58 | Male |  |  | |  | |
| K0AAAP79 | Healthy-control | 42 | Male |  |  | |  | |
| K0AAAP81 | Healthy-control | 42 | Male |  |  | |  | |
| K0AAAP82 | Healthy-control | 53 | Male |  |  | |  | |
| K0AAAP83 | Healthy-control | 47 | Male |  |  | |  | |
| K0AAAP85 | Healthy-control | 60 | Male |  |  | |  | |
| K0AAAP91 | Healthy-control | 44 | Female |  |  | |  | |
| K0AAAP92 | Healthy-control | 51 | Female |  |  | |  | |
| K0AAAP93 | Healthy-control | 45 | Female |  |  | |  | |
| K0AAAP96 | Healthy-control | 57 | Female |  |  | |  | |
| K0AAAP97 | Healthy-control | 42 | Female |  |  | |  | |
| K0AAAP98 | Healthy-control | 48 | Male |  |  | |  | |
| K0AAAP99 | Healthy-control | 45 | Female |  |  | |  | |
| K0AAAQ01 | Healthy-control | 53 | Male |  |  | |  | |
| K0AAAQ03 | Healthy-control | 46 | Female |  |  | |  | |
| K0AAAQ04 | Healthy-control | 42 | Female |  |  | |  | |
| K0AAAQ07 | Healthy-control | 51 | Male |  |  | |  | |
| K0AAAQ10 | Healthy-control | 41 | Male |  |  | |  | |
| K0AAAQ12 | Healthy-control | 41 | Male |  |  | |  | |
| K0AAAQ16 | Healthy-control | 46 | Female |  |  | |  | |
| K0AAAQ20 | Healthy-control | 40 | Male |  |  | |  | |
| K0AAAQ22 | Healthy-control | 53 | Male |  |  | |  | |
| K0AAAQ24 | Healthy-control | 40 | Female |  |  | |  | |
| K0AAAQ26 | Healthy-control | 44 | Female |  |  | |  | |
| K0AAAQ27 | Healthy-control | 42 | Female |  |  | |  | |
| K0AAAQ29 | Healthy-control | 42 | Female |  |  | |  | |
| K0AAAQ32 | Healthy-control | 40 | Female |  |  | |  | |
| K0AAAQ33 | Healthy-control | 51 | Male |  |  | |  | |
| K0AAAQ34 | Healthy-control | 52 | Male |  |  | |  | |
| K0AAAQ37 | Healthy-control | 41 | Female |  |  | |  | |
| K0AAAQ45 | Healthy-control | 47 | Female |  |  | |  | |
| K0AAAQ46 | Healthy-control | 41 | Female |  |  | |  | |
| K0AAAQ47 | Healthy-control | 49 | Male |  |  | |  | |
| K0AAAQ56 | Healthy-control | 47 | Male |  |  | |  | |
| K0AAAQ57 | Healthy-control | 47 | Female |  |  | |  | |
| K0AAAQ59 | Healthy-control | 67 | Male |  |  | |  | |
| K0AAAQ64 | Healthy-control | 45 | Female |  |  | |  | |
| K0AAAQ68 | Healthy-control | 49 | Female |  |  | |  | |
| K0AAAQ70 | Healthy-control | 45 | Female |  |  | |  | |
| K0AAAQ72 | Healthy-control | 60 | Male |  |  | |  | |
| K0AAAQ73 | Healthy-control | 50 | Male |  |  | |  | |
| K0AAAQ74 | Healthy-control | 60 | Male |  |  | |  | |
| K0AAAQ75 | Healthy-control | 55 | Female |  |  | |  | |
| K0AAAQ76 | Healthy-control | 51 | Male |  |  | |  | |
| K0AAAQ79 | Healthy-control | 55 | Female |  |  | |  | |
| K0AAAQ80 | Healthy-control | 44 | Female |  |  | |  | |
| K0AAAQ82 | Healthy-control | 59 | Female |  |  | |  | |
| K0AAAQ83 | Healthy-control | 43 | Female |  |  | |  | |
| K0AAAQ90 | Healthy-control | 58 | Female |  |  | |  | |
| K0AAAQ95 | Healthy-control | 43 | Male |  |  | |  | |
| K0AAAQ96 | Healthy-control | 48 | Female |  |  | |  | |
| K0AAAQ97 | Healthy-control | 40 | Male |  |  | |  | |
| K0AAAQ98 | Healthy-control | 40 | Female |  |  | |  | |
| K0AAAQ99 | Healthy-control | 42 | Female |  |  | |  | |
| K0AAAR02 | Healthy-control | 50 | Female |  |  | |  | |
| K0AAAR03 | Healthy-control | 52 | Male |  |  | |  | |
| K0AAAR06 | Healthy-control | 54 | Female |  |  | |  | |
| K0AAAR08 | Healthy-control | 46 | Male |  |  | |  | |
| K0AAAR10 | Healthy-control | 52 | Female |  |  | |  | |
| K0AAAR13 | Healthy-control | 50 | Female |  |  | |  | |
| K0AAAR14 | Healthy-control | 43 | Female |  |  | |  | |
| K0AAAR15 | Healthy-control | 62 | Female |  |  | |  | |
| K0AAAR24 | Healthy-control | 59 | Female |  |  | |  | |
| K0AAAR26 | Healthy-control | 56 | Male |  |  | |  | |
| K0AAAR27 | Healthy-control | 57 | Male |  |  | |  | |
| K0AAAR29 | Healthy-control | 63 | Male |  |  | |  | |
| K0AAAR30 | Healthy-control | 46 | Male |  |  | |  | |
| K0AAAR34 | Healthy-control | 46 | Male |  |  | |  | |
| K0AAAR35 | Healthy-control | 47 | Male |  |  | |  | |
| K0AAAR36 | Healthy-control | 48 | Male |  |  | |  | |
| K0AAAR37 | Healthy-control | 43 | Female |  |  | |  | |
| K0AAAR41 | Healthy-control | 45 | Female |  |  | |  | |
| K0AAAR42 | Healthy-control | 53 | Male |  |  | |  | |
| K0AAAR46 | Healthy-control | 59 | Female |  |  | |  | |
| K0AAAR48 | Healthy-control | 71 | Female |  |  | |  | |
| K0AAAR50 | Healthy-control | 71 | Female |  |  | |  | |
| K0AAAR52 | Healthy-control | 44 | Female |  |  | |  | |
| K0CAAF38 | Healthy-control | 46 | Female |  |  | |  | |
| K0CAAF39 | Healthy-control | 42 | Female |  |  | |  | |
| K0CAAF42 | Healthy-control | 62 | Female |  |  | |  | |
| K0CAAF43 | Healthy-control | 56 | Female |  |  | |  | |
| K0CAAF44 | Healthy-control | 60 | Female |  |  | |  | |
| K0CAAF45 | Healthy-control | 58 | Male |  |  | |  | |
| K0CAAF47 | Healthy-control | 63 | Male |  |  | |  | |
| K0CAAF48 | Healthy-control | 43 | Female |  |  | |  | |
| K0CAAF49 | Healthy-control | 58 | Female |  |  | |  | |
| K0CAAF50 | Healthy-control | 76 | Male |  |  | |  | |
| K0CAAF51 | Healthy-control | 45 | Female |  |  | |  | |
| K0CAAF53 | Healthy-control | 50 | Female |  |  | |  | |
| K0CAAF58 | Healthy-control | 40 | Male |  |  | |  | |
| K0CAAF62 | Healthy-control | 44 | Female |  |  | |  | |
| K0CAAF63 | Healthy-control | 43 | Female |  |  | |  | |
| K0CAAF64 | Healthy-control | 46 | Male |  |  | |  | |
| K0CAAF76 | Healthy-control | 49 | Female |  |  | |  | |
| K0DAAC92 | Healthy-control | 62 | Female |  |  | |  | |
| K0DAAC98 | Healthy-control | 65 | Female |  |  | |  | |
| K0DAAD02 | Healthy-control | 61 | Female |  |  | |  | |
| K0DAAD07 | Healthy-control | 53 | Female |  |  | |  | |
| K0DAAD08 | Healthy-control | 46 | Female |  |  | |  | |
| K0DAAD10 | Healthy-control | 58 | Female |  |  | |  | |
| K0DAAD13 | Healthy-control | 64 | Female |  |  | |  | |
| K0DAAD14 | Healthy-control | 54 | Female |  |  | |  | |
| K0DAAD15 | Healthy-control | 51 | Female |  |  | |  | |
| K0DAAD19 | Healthy-control | 61 | Female |  |  | |  | |
| K0DAAD20 | Healthy-control | 60 | Female |  |  | |  | |
| K0DAAD22 | Healthy-control | 62 | Female |  |  | |  | |
| K0DAAD24 | Healthy-control | 67 | Female |  |  | |  | |
| K0DAAD25 | Healthy-control | 44 | Female |  |  | |  | |
| K0DAAD26 | Healthy-control | 46 | Female |  |  | |  | |
| K0DAAD27 | Healthy-control | 54 | Female |  |  | |  | |
| K0DAAD28 | Healthy-control | 45 | Female |  |  | |  | |
| K0DAAD30 | Healthy-control | 43 | Female |  |  | |  | |
| K0DAAD31 | Healthy-control | 52 | Female |  |  | |  | |
| K0DAAD33 | Healthy-control | 49 | Female |  |  | |  | |
| K0DAAD34 | Healthy-control | 66 | Female |  |  | |  | |
| K0DAAD35 | Healthy-control | 47 | Female |  |  | |  | |
| K0DAAD36 | Healthy-control | 64 | Female |  |  | |  | |
| K0DAAD40 | Healthy-control | 51 | Female |  |  | |  | |
| K0DAAD41 | Healthy-control | 59 | Female |  |  | |  | |
| K0DAAD42 | Healthy-control | 44 | Female |  |  | |  | |
| K0DAAD44 | Healthy-control | 57 | Female |  |  | |  | |
| K0DAAD45 | Healthy-control | 47 | Female |  |  | |  | |
| K0DAAD47 | Healthy-control | 50 | Female |  |  | |  | |
| K0DAAD48 | Healthy-control | 41 | Female |  |  | |  | |
| K0DAAD50 | Healthy-control | 51 | Female |  |  | |  | |
| K0DAAD53 | Healthy-control | 67 | Male |  |  | |  | |
| K0DAAD56 | Healthy-control | 42 | Female |  |  | |  | |
| K0DAAD58 | Healthy-control | 55 | Male |  |  | |  | |
| K0DAAD59 | Healthy-control | 40 | Female |  |  | |  | |
| K0DAAD60 | Healthy-control | 55 | Female |  |  | |  | |
| K0GAAF13 | Healthy-control | 59 | Male |  |  | |  | |
| K0GAAF32 | Healthy-control | 63 | Male |  |  | |  | |
| K0GAAF59 | Healthy-control | 57 | Male |  |  | |  | |
| K0GAAF85 | Healthy-control | 48 | Male |  |  | |  | |
| K0GAAF86 | Healthy-control | 64 | Male |  |  | |  | |
| K0GAAF89 | Healthy-control | 43 | Male |  |  | |  | |
| K0GAAF90 | Healthy-control | 63 | Male |  |  | |  | |
| K0GAAF91 | Healthy-control | 48 | Female |  |  | |  | |
| K0GAAF94 | Healthy-control | 56 | Female |  |  | |  | |
| K0GAAG04 | Healthy-control | 63 | Female |  |  | |  | |
| K0GAAG09 | Healthy-control | 41 | Female |  |  | |  | |
| K0GAAG11 | Healthy-control | 41 | Male |  |  | |  | |
| K0GAAG14 | Healthy-control | 48 | Female |  |  | |  | |
| K0GAAG37 | Healthy-control | 55 | Male |  |  | |  | |
| K0GAAG41 | Healthy-control | 49 | Male |  |  | |  | |
| K0GAAG42 | Healthy-control | 66 | Male |  |  | |  | |
| K0GAAG49 | Healthy-control | 64 | Female |  |  | |  | |
| K0GAAG53 | Healthy-control | 40 | Female |  |  | |  | |
| K0GAAG61 | Healthy-control | 41 | Male |  |  | |  | |
| K0GAAG64 | Healthy-control | 45 | Male |  |  | |  | |
| K0GAAG65 | Healthy-control | 55 | Male |  |  | |  | |
| K0GAAG68 | Healthy-control | 54 | Female |  |  | |  | |
| K0GAAG69 | Healthy-control | 63 | Female |  |  | |  | |
| K0GAAG70 | Healthy-control | 40 | Male |  |  | |  | |
| K0GAAG73 | Healthy-control | 51 | Female |  |  | |  | |
| K0GAAG81 | Healthy-control | 67 | Male |  |  | |  | |
| K0GAAG93 | Healthy-control | 64 | Male |  |  | |  | |
| K0GAAH06 | Healthy-control | 46 | Male |  |  | |  | |
| K0GAAH07 | Healthy-control | 60 | Female |  |  | |  | |
| K0GAAH10 | Healthy-control | 43 | Male |  |  | |  | |
| K0GAAH18 | Healthy-control | 45 | Female |  |  | |  | |
| K0GAAH21 | Healthy-control | 64 | Female |  |  | |  | |
| K0GAAH25 | Healthy-control | 54 | Female |  |  | |  | |
| K0GAAH32 | Healthy-control | 40 | Male |  |  | |  | |
| K0GAAH39 | Healthy-control | 57 | Male |  |  | |  | |
| K0GAAH46 | Healthy-control | 40 | Female |  |  | |  | |
| K0GAAH48 | Healthy-control | 42 | Male |  |  | |  | |
| K0GAAH49 | Healthy-control | 64 | Male |  |  | |  | |
| K0GAAH52 | Healthy-control | 44 | Male |  |  | |  | |
| K0GAAH58 | Healthy-control | 54 | Female |  |  | |  | |
| K0GAAH59 | Healthy-control | 63 | Male |  |  | |  | |
| K0GAAH60 | Healthy-control | 42 | Male |  |  | |  | |
| K0GAAH61 | Healthy-control | 41 | Female |  |  | |  | |
| K0GAAH62 | Healthy-control | 48 | Male |  |  | |  | |
| K0GAAH63 | Healthy-control | 48 | Male |  |  | |  | |
| K0GAAH64 | Healthy-control | 48 | Female |  |  | |  | |
| K0GAAH65 | Healthy-control | 50 | Male |  |  | |  | |
| K0GAAH66 | Healthy-control | 51 | Female |  |  | |  | |
| K0GAAH67 | Healthy-control | 41 | Female |  |  | |  | |
| K0GAAH68 | Healthy-control | 42 | Male |  |  | |  | |
| K0GAAH69 | Healthy-control | 45 | Male |  |  | |  | |
| K0GAAH72 | Healthy-control | 48 | Female |  |  | |  | |
| K0GAAH73 | Healthy-control | 51 | Male |  |  | |  | |
| K0GAAH74 | Healthy-control | 50 | Female |  |  | |  | |
| K0GAAH75 | Healthy-control | 56 | Female |  |  | |  | |
| K0GAAH76 | Healthy-control | 44 | Female |  |  | |  | |
| K0GAAH77 | Healthy-control | 60 | Female |  |  | |  | |
| K0GAAH80 | Healthy-control | 48 | Male |  |  | |  | |
| K0GAAH81 | Healthy-control | 55 | Female |  |  | |  | |
| K0GAAH82 | Healthy-control | 60 | Male |  |  | |  | |
| K0GAAH83 | Healthy-control | 44 | Female |  |  | |  | |
| K0GAAH87 | Healthy-control | 43 | Male |  |  | |  | |
| K0GAAH90 | Healthy-control | 61 | Male |  |  | |  | |
| K0GAAH91 | Healthy-control | 61 | Male |  |  | |  | |
| K0GAAH92 | Healthy-control | 58 | Female |  |  | |  | |
| K0GAAH93 | Healthy-control | 57 | Female |  |  | |  | |
| K0GAAH94 | Healthy-control | 46 | Male |  |  | |  | |
| K0GAAH95 | Healthy-control | 63 | Male |  |  | |  | |
| K0GAAH96 | Healthy-control | 49 | Female |  |  | |  | |
| K0GAAH97 | Healthy-control | 48 | Male |  |  | |  | |
| K0GAAH98 | Healthy-control | 49 | Female |  |  | |  | |
| K0GAAI01 | Healthy-control | 73 | Male |  |  | |  | |
| K0GAAI03 | Healthy-control | 50 | Male |  |  | |  | |
| K0GAAI04 | Healthy-control | 44 | Female |  |  | |  | |
| K0GAAI05 | Healthy-control | 49 | Male |  |  | |  | |
| K0GAAI06 | Healthy-control | 42 | Female |  |  | |  | |
| K0GAAI08 | Healthy-control | 63 | Male |  |  | |  | |
| K0GAAI10 | Healthy-control | 49 | Female |  |  | |  | |
| K0GAAI12 | Healthy-control | 43 | Male |  |  | |  | |
| K0GAAI13 | Healthy-control | 57 | Male |  |  | |  | |
| K0GAAI15 | Healthy-control | 49 | Female |  |  | |  | |
| K0GAAI18 | Healthy-control | 61 | Female |  |  | |  | |
| K0GAAI20 | Healthy-control | 50 | Female |  |  | |  | |
| K0GAAI21 | Healthy-control | 48 | Female |  |  | |  | |
| K0GAAI22 | Healthy-control | 44 | Female |  |  | |  | |
| K0GAAI23 | Healthy-control | 51 | Male |  |  | |  | |
| K0GAAI25 | Healthy-control | 48 | Male |  |  | |  | |
| K0GAAI43 | Healthy-control | 47 | Male |  |  | |  | |
| K0GAAI44 | Healthy-control | 43 | Male |  |  | |  | |
| K0GAAI45 | Healthy-control | 63 | Male |  |  | |  | |
| K0GAAI46 | Healthy-control | 64 | Female |  |  | |  | |
| K0GAAI47 | Healthy-control | 43 | Female |  |  | |  | |
| K0GAAI49 | Healthy-control | 53 | Female |  |  | |  | |
| K0GAAI50 | Healthy-control | 54 | Male |  |  | |  | |
| K0GAAI51 | Healthy-control | 46 | Female |  |  | |  | |
| K0GAAI53 | Healthy-control | 51 | Male |  |  | |  | |
| K0GAAI54 | Healthy-control | 59 | Male |  |  | |  | |
| K0GAAI56 | Healthy-control | 47 | Female |  |  | |  | |
| K0GAAI57 | Healthy-control | 52 | Male |  |  | |  | |
| K0GAAI60 | Healthy-control | 40 | Female |  |  | |  | |
| K0GAAI61 | Healthy-control | 61 | Female |  |  | |  | |
| K0GAAI62 | Healthy-control | 51 | Male |  |  | |  | |
| K0GAAI63 | Healthy-control | 47 | Male |  |  | |  | |
| K0GAAI64 | Healthy-control | 54 | Male |  |  | |  | |
| K0GAAI68 | Healthy-control | 49 | Female |  |  | |  | |
| K6AAAB38 | Healthy-control | 42 | Male |  |  | |  | |
| K6AAAB56 | Healthy-control | 57 | Male |  |  | |  | |
| K0AAAQ50 | Healthy-control | 60 | Male |  |  | |  | |
| K0AAAR89 | Healthy-control | 46 | Female |  |  | |  | |
| K0AAAR90 | Healthy-control | 47 | Male |  |  | |  | |
| K0CAAG24 | Healthy-control | 76 | Male |  |  | |  | |
| K0DAAE01 | Healthy-control | 48 | Female |  |  | |  | |
| K0DAAE57 | Healthy-control | 44 | Female |  |  | |  | |
| K0DAAE69 | Healthy-control | 44 | Male |  |  | |  | |
| K0GAAI93 | Healthy-control | 40 | Male |  |  | |  | |
| K0GAAJ08 | Healthy-control | 50 | Female |  |  | |  | |
| K0GAAJ32 | Healthy-control | 50 | Male |  |  | |  | |
| K0GAAJ33 | Healthy-control | 48 | Female |  |  | |  | |
