# Supplementary material for: Combination of Hotspot Mutations With Methylation and Fragmentomic Profiles to Enhance Multi‐Cancer Early Detection
Source: Cancer Med. 2025 Jan 3;14(1):e70575. doi: 10.1002/cam4.70575 (PMC11695824; doi:10.1002/cam4.70575)
Supplement: Supplementary file 3 — Table S2. Details of panel 700 hotspot mutations in 23 genes. [file CAM4-14-e70575-s002.docx]

| **Table S2: Details of panel 700 hotspot mutations in 23 genes** | | | | | | |  | |  | |  | |  | |  | |  | |  | |  |  |  | |  | |  | |  | |  | |  | |  | |  | |  | |  | |  | |  | |  | |  | |  | |  | |  |  |  | |  | |  | |  | |  | |  | |  | |  | |  |  |  |  |
| --- | --- | --- | --- | --- | --- | --- | --- | --- | --- | --- | --- | --- | --- | --- | --- | --- | --- | --- | --- | --- | --- | --- | --- | --- | --- | --- | --- | --- | --- | --- | --- | --- | --- | --- | --- | --- | --- | --- | --- | --- | --- | --- | --- | --- | --- | --- | --- | --- | --- | --- | --- | --- | --- | --- | --- | --- | --- | --- | --- | --- | --- | --- | --- | --- | --- | --- | --- | --- | --- | --- | --- | --- | --- | --- | --- | --- |
| **Gene** | **Position** | **AA mutation** | **CDS mutation** | **Variant Classification** | **Class** | **Actionable mutation FDA approved drugs and NCCN 2024 recommended)** | |  | |  | |  | |  | |  | |  | |  | | | |  | |  | |  | |  | |  | |  | |  | |  | |  | |  | |  | |  | |  | |  | |  | |  | |  | |  | |  | |  | |  | |  | |  | |  | |  | |  |  |  |
| ACVR2A | chr2:147926123-147926123 | K437Rfs*5 | AA>A | Deletion | COSMIC(CRC) | No | |  | |  | |  | |  | |  | |  | |  | | | |  | |  | |  | |  | |  | |  | |  | |  | |  | |  | |  | |  | |  | |  | |  | |  | |  | |  | |  | |  | |  | |  | |  | |  | |  | |  |  |  |
| AKT1 | chr14:104780214-104780214 | E17K | C>T | Single Nucleotide Variants (SNVs) | COSMIC(Breast)-Tissue(Breast) | Yes | |  | |  | |  | |  | |  | |  | |  | | | |  | |  | |  | |  | |  | |  | |  | |  | |  | |  | |  | |  | |  | |  | |  | |  | |  | |  | |  | |  | |  | |  | |  | |  | |  | |  |  |  |
| ALK | chr2:29220725-29220725 | R1209Q | C>T | Single Nucleotide Variants (SNVs) | Tissue(Gastric) | No | |  | |  | |  | |  | |  | |  | |  | | | |  | |  | |  | |  | |  | |  | |  | |  | |  | |  | |  | |  | |  | |  | |  | |  | |  | |  | |  | |  | |  | |  | |  | |  | |  | |  |  |  |
| ALK | chr2:29220734-29220734 | S1206C | G>C | Single Nucleotide Variants (SNVs) | COSMIC(Lung) | No | |  | |  | |  | |  | |  | |  | |  | | | |  | |  | |  | |  | |  | |  | |  | |  | |  | |  | |  | |  | |  | |  | |  | |  | |  | |  | |  | |  | |  | |  | |  | |  | |  | |  |  |  |
| ALK | chr2:29220734-29220734 | S1206F | G>A | Single Nucleotide Variants (SNVs) | COSMIC(Lung) | No | |  | |  | |  | |  | |  | |  | |  | | | |  | |  | |  | |  | |  | |  | |  | |  | |  | |  | |  | |  | |  | |  | |  | |  | |  | |  | |  | |  | |  | |  | |  | |  | |  | |  |  |  |
| ALK | chr2:29220734-29220734 | S1206Y | G>T | Single Nucleotide Variants (SNVs) | COSMIC(Lung) | No | |  | |  | |  | |  | |  | |  | |  | | | |  | |  | |  | |  | |  | |  | |  | |  | |  | |  | |  | |  | |  | |  | |  | |  | |  | |  | |  | |  | |  | |  | |  | |  | |  | |  |  |  |
| ALK | chr2:29220743-29220743 | G1202Del | TCTC>T | Deletion | COSMIC(Lung) | No | |  | |  | |  | |  | |  | |  | |  | | | |  | |  | |  | |  | |  | |  | |  | |  | |  | |  | |  | |  | |  | |  | |  | |  | |  | |  | |  | |  | |  | |  | |  | |  | |  | |  |  |  |
| ALK | chr2:29220747-29220747 | G1202R | C>G | Single Nucleotide Variants (SNVs) | COSMIC(Lung) | Yes | |  | |  | |  | |  | |  | |  | |  | | | |  | |  | |  | |  | |  | |  | |  | |  | |  | |  | |  | |  | |  | |  | |  | |  | |  | |  | |  | |  | |  | |  | |  | |  | |  | |  |  |  |
| ALK | chr2:29220747-29220747 | G1202R | C>T | Single Nucleotide Variants (SNVs) | COSMIC(Lung) | Yes | |  | |  | |  | |  | |  | |  | |  | | | |  | |  | |  | |  | |  | |  | |  | |  | |  | |  | |  | |  | |  | |  | |  | |  | |  | |  | |  | |  | |  | |  | |  | |  | |  | |  |  |  |
| AMER1 | chrX:64191396-64191396 | R631* | G>A | Single Nucleotide Variants (SNVs) | Tissue(CRC)-COSMIC(CRC) | No | |  | |  | |  | |  | |  | |  | |  | | | |  | |  | |  | |  | |  | |  | |  | |  | |  | |  | |  | |  | |  | |  | |  | |  | |  | |  | |  | |  | |  | |  | |  | |  | |  | |  |  |  |
| AMER1 | chrX:64192215-64192215 | R358* | G>A | Single Nucleotide Variants (SNVs) | Tissue(CRC)-COSMIC(CRC) | No | |  | |  | |  | |  | |  | |  | |  | | | |  | |  | |  | |  | |  | |  | |  | |  | |  | |  | |  | |  | |  | |  | |  | |  | |  | |  | |  | |  | |  | |  | |  | |  | |  | |  |  |  |
| AMER1 | chrX:64192238-64192238 | R350Kfs*28 | C>CT | Insertion | Tissue(CRC) | No | |  | |  | |  | |  | |  | |  | |  | | | |  | |  | |  | |  | |  | |  | |  | |  | |  | |  | |  | |  | |  | |  | |  | |  | |  | |  | |  | |  | |  | |  | |  | |  | |  | |  |  |  |
| APC | chr5:112780895-112780895 | R213* | C>T | Single Nucleotide Variants (SNVs) | Tissue(CRC)-COSMIC(CRC) | No | |  | |  | |  | |  | |  | |  | |  | | | |  | |  | |  | |  | |  | |  | |  | |  | |  | |  | |  | |  | |  | |  | |  | |  | |  | |  | |  | |  | |  | |  | |  | |  | |  | |  |  |  |
| APC | chr5:112780901-112780901 | Q215* | C>T | Single Nucleotide Variants (SNVs) | Tissue(CRC) | No | |  | |  | |  | |  | |  | |  | |  | | | |  | |  | |  | |  | |  | |  | |  | |  | |  | |  | |  | |  | |  | |  | |  | |  | |  | |  | |  | |  | |  | |  | |  | |  | |  | |  |  |  |
| APC | chr5:112792494-112792494 | R232* | C>T | Single Nucleotide Variants (SNVs) | Tissue(CRC)-COSMIC(CRC) | No | |  | |  | |  | |  | |  | |  | |  | | | |  | |  | |  | |  | |  | |  | |  | |  | |  | |  | |  | |  | |  | |  | |  | |  | |  | |  | |  | |  | |  | |  | |  | |  | |  | |  |  |  |
| APC | chr5:112815507-112815507 | R283* | C>T | Single Nucleotide Variants (SNVs) | COSMIC(CRC) | No | |  | |  | |  | |  | |  | |  | |  | | | |  | |  | |  | |  | |  | |  | |  | |  | |  | |  | |  | |  | |  | |  | |  | |  | |  | |  | |  | |  | |  | |  | |  | |  | |  | |  |  |  |
| APC | chr5:112815564-112815564 | R302* | C>T | Single Nucleotide Variants (SNVs) | Tissue(CRC)-COSMIC(CRC) | No | |  | |  | |  | |  | |  | |  | |  | | | |  | |  | |  | |  | |  | |  | |  | |  | |  | |  | |  | |  | |  | |  | |  | |  | |  | |  | |  | |  | |  | |  | |  | |  | |  | |  |  |  |
| APC | chr5:112828889-112828889 | R554* | C>T | Single Nucleotide Variants (SNVs) | Tissue(CRC)-COSMIC(CRC) | No | |  | |  | |  | |  | |  | |  | |  | | | |  | |  | |  | |  | |  | |  | |  | |  | |  | |  | |  | |  | |  | |  | |  | |  | |  | |  | |  | |  | |  | |  | |  | |  | |  | |  |  |  |
| APC | chr5:112828919-112828919 | R564* | C>T | Single Nucleotide Variants (SNVs) | COSMIC(CRC) | No | |  | |  | |  | |  | |  | |  | |  | | | |  | |  | |  | |  | |  | |  | |  | |  | |  | |  | |  | |  | |  | |  | |  | |  | |  | |  | |  | |  | |  | |  | |  | |  | |  | |  |  |  |
| APC | chr5:112838007-112838007 | R805* | C>T | Single Nucleotide Variants (SNVs) | Tissue(CRC)-COSMIC(CRC) | No | |  | |  | |  | |  | |  | |  | |  | | | |  | |  | |  | |  | |  | |  | |  | |  | |  | |  | |  | |  | |  | |  | |  | |  | |  | |  | |  | |  | |  | |  | |  | |  | |  | |  |  |  |
| APC | chr5:112838220-112838220 | R876* | C>T | Single Nucleotide Variants (SNVs) | Tissue(CRC)-COSMIC(CRC) | No | |  | |  | |  | |  | |  | |  | |  | | | |  | |  | |  | |  | |  | |  | |  | |  | |  | |  | |  | |  | |  | |  | |  | |  | |  | |  | |  | |  | |  | |  | |  | |  | |  | |  |  |  |
| APC | chr5:112838934-112838934 | R1114* | C>T | Single Nucleotide Variants (SNVs) | Tissue(CRC)-COSMIC(CRC) | No | |  | |  | |  | |  | |  | |  | |  | | | |  | |  | |  | |  | |  | |  | |  | |  | |  | |  | |  | |  | |  | |  | |  | |  | |  | |  | |  | |  | |  | |  | |  | |  | |  | |  |  |  |
| APC | chr5:112839514-112839514 | E1309Dfs*4 | TAAAAG>T | Deletion | Tissue(CRC) | No | |  | |  | |  | |  | |  | |  | |  | | | |  | |  | |  | |  | |  | |  | |  | |  | |  | |  | |  | |  | |  | |  | |  | |  | |  | |  | |  | |  | |  | |  | |  | |  | |  | |  |  |  |
| APC | chr5:112839519-112839519 | E1309* | G>T | Single Nucleotide Variants (SNVs) | Tissue(CRC) | No | |  | |  | |  | |  | |  | |  | |  | | | |  | |  | |  | |  | |  | |  | |  | |  | |  | |  | |  | |  | |  | |  | |  | |  | |  | |  | |  | |  | |  | |  | |  | |  | |  | |  |  |  |
| APC | chr5:112839520-112839520 | E1309Dfs*4 | AAAAGA>A | Deletion | Tissue(CRC)-COSMIC(CRC) | No | |  | |  | |  | |  | |  | |  | |  | | | |  | |  | |  | |  | |  | |  | |  | |  | |  | |  | |  | |  | |  | |  | |  | |  | |  | |  | |  | |  | |  | |  | |  | |  | |  | |  |  |  |
| APC | chr5:112839528-112839528 | G1312* | G>T | Single Nucleotide Variants (SNVs) | COSMIC(CRC) | No | |  | |  | |  | |  | |  | |  | |  | | | |  | |  | |  | |  | |  | |  | |  | |  | |  | |  | |  | |  | |  | |  | |  | |  | |  | |  | |  | |  | |  | |  | |  | |  | |  | |  |  |  |
| APC | chr5:112839538-112839538 | S1315* | C>A | Single Nucleotide Variants (SNVs) | Tissue(CRC) | No | |  | |  | |  | |  | |  | |  | |  | | | |  | |  | |  | |  | |  | |  | |  | |  | |  | |  | |  | |  | |  | |  | |  | |  | |  | |  | |  | |  | |  | |  | |  | |  | |  | |  |  |  |
| APC | chr5:112839538-112839538 | S1315* | C>G | Single Nucleotide Variants (SNVs) | Tissue(CRC)-COSMIC(CRC) | No | |  | |  | |  | |  | |  | |  | |  | | | |  | |  | |  | |  | |  | |  | |  | |  | |  | |  | |  | |  | |  | |  | |  | |  | |  | |  | |  | |  | |  | |  | |  | |  | |  | |  |  |  |
| APC | chr5:112839548-112839548 | P1319Lfs*2 | TC>T | Deletion | Tissue(CRC) | No | |  | |  | |  | |  | |  | |  | |  | | | |  | |  | |  | |  | |  | |  | |  | |  | |  | |  | |  | |  | |  | |  | |  | |  | |  | |  | |  | |  | |  | |  | |  | |  | |  | |  |  |  |
| APC | chr5:112839549-112839549 | P1319Lfs*2 | CC>C | Deletion | Tissue(CRC)-COSMIC(CRC) | No | |  | |  | |  | |  | |  | |  | |  | | | |  | |  | |  | |  | |  | |  | |  | |  | |  | |  | |  | |  | |  | |  | |  | |  | |  | |  | |  | |  | |  | |  | |  | |  | |  | |  |  |  |
| APC | chr5:112839558-112839558 | E1322* | G>T | Single Nucleotide Variants (SNVs) | COSMIC(CRC) | No | |  | |  | |  | |  | |  | |  | |  | | | |  | |  | |  | |  | |  | |  | |  | |  | |  | |  | |  | |  | |  | |  | |  | |  | |  | |  | |  | |  | |  | |  | |  | |  | |  | |  |  |  |
| APC | chr5:112839606-112839606 | Q1338* | C>T | Single Nucleotide Variants (SNVs) | Tissue(CRC)-COSMIC(CRC) | No | |  | |  | |  | |  | |  | |  | |  | | | |  | |  | |  | |  | |  | |  | |  | |  | |  | |  | |  | |  | |  | |  | |  | |  | |  | |  | |  | |  | |  | |  | |  | |  | |  | |  |  |  |
| APC | chr5:112839651-112839651 | E1353* | G>T | Single Nucleotide Variants (SNVs) | COSMIC(CRC) | No | |  | |  | |  | |  | |  | |  | |  | | | |  | |  | |  | |  | |  | |  | |  | |  | |  | |  | |  | |  | |  | |  | |  | |  | |  | |  | |  | |  | |  | |  | |  | |  | |  | |  |  |  |
| APC | chr5:112839693-112839693 | Q1367* | C>T | Single Nucleotide Variants (SNVs) | Tissue(CRC)-COSMIC(CRC) | No | |  | |  | |  | |  | |  | |  | |  | | | |  | |  | |  | |  | |  | |  | |  | |  | |  | |  | |  | |  | |  | |  | |  | |  | |  | |  | |  | |  | |  | |  | |  | |  | |  | |  |  |  |
| APC | chr5:112839714-112839714 | E1374* | G>T | Single Nucleotide Variants (SNVs) | Tissue(CRC) | No | |  | |  | |  | |  | |  | |  | |  | | | |  | |  | |  | |  | |  | |  | |  | |  | |  | |  | |  | |  | |  | |  | |  | |  | |  | |  | |  | |  | |  | |  | |  | |  | |  | |  |  |  |
| APC | chr5:112839726-112839726 | Q1378* | C>T | Single Nucleotide Variants (SNVs) | COSMIC(CRC) | No | |  | |  | |  | |  | |  | |  | |  | | | |  | |  | |  | |  | |  | |  | |  | |  | |  | |  | |  | |  | |  | |  | |  | |  | |  | |  | |  | |  | |  | |  | |  | |  | |  | |  |  |  |
| APC | chr5:112839729-112839729 | E1379* | G>T | Single Nucleotide Variants (SNVs) | COSMIC(CRC) | No | |  | |  | |  | |  | |  | |  | |  | | | |  | |  | |  | |  | |  | |  | |  | |  | |  | |  | |  | |  | |  | |  | |  | |  | |  | |  | |  | |  | |  | |  | |  | |  | |  | |  |  |  |
| APC | chr5:112839782-112839782 | R1399Ffs*9 | TGA>T | Deletion | Tissue(CRC) | No | |  | |  | |  | |  | |  | |  | |  | | | |  | |  | |  | |  | |  | |  | |  | |  | |  | |  | |  | |  | |  | |  | |  | |  | |  | |  | |  | |  | |  | |  | |  | |  | |  | |  |  |  |
| APC | chr5:112839783-112839783 | E1397* | G>T | Single Nucleotide Variants (SNVs) | Tissue(CRC)-COSMIC(CRC) | No | |  | |  | |  | |  | |  | |  | |  | | | |  | |  | |  | |  | |  | |  | |  | |  | |  | |  | |  | |  | |  | |  | |  | |  | |  | |  | |  | |  | |  | |  | |  | |  | |  | |  |  |  |
| APC | chr5:112839793-112839793 | S1400* | C>A | Single Nucleotide Variants (SNVs) | Tissue(CRC) | No | |  | |  | |  | |  | |  | |  | |  | | | |  | |  | |  | |  | |  | |  | |  | |  | |  | |  | |  | |  | |  | |  | |  | |  | |  | |  | |  | |  | |  | |  | |  | |  | |  | |  |  |  |
| APC | chr5:112839795-112839795 | I1401Mfs*14 | AT>A | Deletion | Tissue(CRC) | No | |  | |  | |  | |  | |  | |  | |  | | | |  | |  | |  | |  | |  | |  | |  | |  | |  | |  | |  | |  | |  | |  | |  | |  | |  | |  | |  | |  | |  | |  | |  | |  | |  | |  |  |  |
| APC | chr5:112839807-112839807 | Q1406Rfs*9 | GT>G | Deletion | Tissue(CRC) | No | |  | |  | |  | |  | |  | |  | |  | | | |  | |  | |  | |  | |  | |  | |  | |  | |  | |  | |  | |  | |  | |  | |  | |  | |  | |  | |  | |  | |  | |  | |  | |  | |  | |  |  |  |
| APC | chr5:112839810-112839810 | Q1406* | C>T | Single Nucleotide Variants (SNVs) | Tissue(CRC)-COSMIC(CRC) | No | |  | |  | |  | |  | |  | |  | |  | | | |  | |  | |  | |  | |  | |  | |  | |  | |  | |  | |  | |  | |  | |  | |  | |  | |  | |  | |  | |  | |  | |  | |  | |  | |  | |  |  |  |
| APC | chr5:112839816-112839816 | E1408* | G>T | Single Nucleotide Variants (SNVs) | COSMIC(CRC) | No | |  | |  | |  | |  | |  | |  | |  | | | |  | |  | |  | |  | |  | |  | |  | |  | |  | |  | |  | |  | |  | |  | |  | |  | |  | |  | |  | |  | |  | |  | |  | |  | |  | |  |  |  |
| APC | chr5:112839879-112839879 | Q1429* | C>T | Single Nucleotide Variants (SNVs) | COSMIC(CRC) | No | |  | |  | |  | |  | |  | |  | |  | | | |  | |  | |  | |  | |  | |  | |  | |  | |  | |  | |  | |  | |  | |  | |  | |  | |  | |  | |  | |  | |  | |  | |  | |  | |  | |  |  |  |
| APC | chr5:112839942-112839942 | R1450* | C>T | Single Nucleotide Variants (SNVs) | Tissue(CRC)-COSMIC(CRC,Gastric) | No | |  | |  | |  | |  | |  | |  | |  | | | |  | |  | |  | |  | |  | |  | |  | |  | |  | |  | |  | |  | |  | |  | |  | |  | |  | |  | |  | |  | |  | |  | |  | |  | |  | |  |  |  |
| APC | chr5:112839986-112839986 | S1465Wfs*3 | GAG>G | Deletion | COSMIC(CRC) | No | |  | |  | |  | |  | |  | |  | |  | | | |  | |  | |  | |  | |  | |  | |  | |  | |  | |  | |  | |  | |  | |  | |  | |  | |  | |  | |  | |  | |  | |  | |  | |  | |  | |  |  |  |
| APC | chr5:112840254-112840254 | E1554* | G>T | Single Nucleotide Variants (SNVs) | Tissue(CRC) | No | |  | |  | |  | |  | |  | |  | |  | | | |  | |  | |  | |  | |  | |  | |  | |  | |  | |  | |  | |  | |  | |  | |  | |  | |  | |  | |  | |  | |  | |  | |  | |  | |  | |  |  |  |
| APC | chr5:112840260-112840260 | T1556Nfs*3 | A>AA | Insertion | COSMIC(CRC,Gastric) | No | |  | |  | |  | |  | |  | |  | |  | | | |  | |  | |  | |  | |  | |  | |  | |  | |  | |  | |  | |  | |  | |  | |  | |  | |  | |  | |  | |  | |  | |  | |  | |  | |  | |  |  |  |
| ARID1A | chr1:26780591-26780591 | R2232Gfs*35 | GC>G | Deletion | Tissue(HCC) | No | |  | |  | |  | |  | |  | |  | |  | | | |  | |  | |  | |  | |  | |  | |  | |  | |  | |  | |  | |  | |  | |  | |  | |  | |  | |  | |  | |  | |  | |  | |  | |  | |  | |  |  |  |
| ARID1A | chr1:26780597-26780597 | A2235Rfs*30 | GGCTGCCC>G | Deletion | Tissue(HCC) | No | |  | |  | |  | |  | |  | |  | |  | | | |  | |  | |  | |  | |  | |  | |  | |  | |  | |  | |  | |  | |  | |  | |  | |  | |  | |  | |  | |  | |  | |  | |  | |  | |  | |  |  |  |
| ARID1A | chr1:26780608-26780608 | L2238Afs*40 | C>CG | Insertion | Tissue(Gastric) | No | |  | |  | |  | |  | |  | |  | |  | | | |  | |  | |  | |  | |  | |  | |  | |  | |  | |  | |  | |  | |  | |  | |  | |  | |  | |  | |  | |  | |  | |  | |  | |  | |  | |  |  |  |
| BRAF | chr7:140753332-140753332 | K601N | T>A | Single Nucleotide Variants (SNVs) | COSMIC(Lung) | No | |  | |  | |  | |  | |  | |  | |  | | | |  | |  | |  | |  | |  | |  | |  | |  | |  | |  | |  | |  | |  | |  | |  | |  | |  | |  | |  | |  | |  | |  | |  | |  | |  | |  |  |  |
| BRAF | chr7:140753332-140753332 | K601N | T>G | Single Nucleotide Variants (SNVs) | COSMIC(Lung) | No | |  | |  | |  | |  | |  | |  | |  | | | |  | |  | |  | |  | |  | |  | |  | |  | |  | |  | |  | |  | |  | |  | |  | |  | |  | |  | |  | |  | |  | |  | |  | |  | |  | |  |  |  |
| BRAF | chr7:140753333-140753333 | K601R | T>C | Single Nucleotide Variants (SNVs) | COSMIC(Lung) | No | |  | |  | |  | |  | |  | |  | |  | | | |  | |  | |  | |  | |  | |  | |  | |  | |  | |  | |  | |  | |  | |  | |  | |  | |  | |  | |  | |  | |  | |  | |  | |  | |  | |  |  |  |
| BRAF | chr7:140753334-140753334 | K601E | T>C | Single Nucleotide Variants (SNVs) | COSMIC(Lung)-Tissue(CRC) | No | |  | |  | |  | |  | |  | |  | |  | | | |  | |  | |  | |  | |  | |  | |  | |  | |  | |  | |  | |  | |  | |  | |  | |  | |  | |  | |  | |  | |  | |  | |  | |  | |  | |  |  |  |
| BRAF | chr7:140753334-140753334 | K601Q | T>G | Single Nucleotide Variants (SNVs) | COSMIC(Lung) | No | |  | |  | |  | |  | |  | |  | |  | | | |  | |  | |  | |  | |  | |  | |  | |  | |  | |  | |  | |  | |  | |  | |  | |  | |  | |  | |  | |  | |  | |  | |  | |  | |  | |  |  |  |
| BRAF | chr7:140753335-140753335 | V600D | CA>AT | Multi- nucleotide variants (MNVs) | COSMIC(Lung,CRC) | No | |  | |  | |  | |  | |  | |  | |  | | | |  | |  | |  | |  | |  | |  | |  | |  | |  | |  | |  | |  | |  | |  | |  | |  | |  | |  | |  | |  | |  | |  | |  | |  | |  | |  |  |  |
| BRAF | chr7:140753335-140753335 | V600D | CA>GT | Multi- nucleotide variants (MNVs) | COSMIC(Lung,CRC) | No | |  | |  | |  | |  | |  | |  | |  | | | |  | |  | |  | |  | |  | |  | |  | |  | |  | |  | |  | |  | |  | |  | |  | |  | |  | |  | |  | |  | |  | |  | |  | |  | |  | |  |  |  |
| BRAF | chr7:140753336-140753336 | V600A | A>G | Single Nucleotide Variants (SNVs) | COSMIC(Lung,CRC) | No | |  | |  | |  | |  | |  | |  | |  | | | |  | |  | |  | |  | |  | |  | |  | |  | |  | |  | |  | |  | |  | |  | |  | |  | |  | |  | |  | |  | |  | |  | |  | |  | |  | |  |  |  |
| BRAF | chr7:140753336-140753336 | V600E | A>T | Single Nucleotide Variants (SNVs) | COSMIC(Lung,CRC,Breast,Gastric,HCC)-Tissue(CRC) | Yes | |  | |  | |  | |  | |  | |  | |  | | | |  | |  | |  | |  | |  | |  | |  | |  | |  | |  | |  | |  | |  | |  | |  | |  | |  | |  | |  | |  | |  | |  | |  | |  | |  | |  |  |  |
| BRAF | chr7:140753336-140753336 | V600G | A>C | Single Nucleotide Variants (SNVs) | COSMIC(Lung,CRC) | No | |  | |  | |  | |  | |  | |  | |  | | | |  | |  | |  | |  | |  | |  | |  | |  | |  | |  | |  | |  | |  | |  | |  | |  | |  | |  | |  | |  | |  | |  | |  | |  | |  | |  |  |  |
| BRAF | chr7:140753336-140753336 | V600K | AC>TT | Multi- nucleotide variants (MNVs) | COSMIC(Lung,CRC) | No | |  | |  | |  | |  | |  | |  | |  | | | |  | |  | |  | |  | |  | |  | |  | |  | |  | |  | |  | |  | |  | |  | |  | |  | |  | |  | |  | |  | |  | |  | |  | |  | |  | |  |  |  |
| BRAF | chr7:140753336-140753336 | V600R | AC>CG | Multi- nucleotide variants (MNVs) | COSMIC(Lung,CRC) | No | |  | |  | |  | |  | |  | |  | |  | | | |  | |  | |  | |  | |  | |  | |  | |  | |  | |  | |  | |  | |  | |  | |  | |  | |  | |  | |  | |  | |  | |  | |  | |  | |  | |  |  |  |
| BRAF | chr7:140753336-140753336 | V600R | AC>CT | Multi- nucleotide variants (MNVs) | COSMIC(Lung,CRC) | No | |  | |  | |  | |  | |  | |  | |  | | | |  | |  | |  | |  | |  | |  | |  | |  | |  | |  | |  | |  | |  | |  | |  | |  | |  | |  | |  | |  | |  | |  | |  | |  | |  | |  |  |  |
| BRAF | chr7:140753337-140753337 | V600L | C>A | Single Nucleotide Variants (SNVs) | COSMIC(Lung,CRC) | No | |  | |  | |  | |  | |  | |  | |  | | | |  | |  | |  | |  | |  | |  | |  | |  | |  | |  | |  | |  | |  | |  | |  | |  | |  | |  | |  | |  | |  | |  | |  | |  | |  | |  |  |  |
| BRAF | chr7:140753337-140753337 | V600L | C>G | Single Nucleotide Variants (SNVs) | COSMIC(Lung,CRC) | No | |  | |  | |  | |  | |  | |  | |  | | | |  | |  | |  | |  | |  | |  | |  | |  | |  | |  | |  | |  | |  | |  | |  | |  | |  | |  | |  | |  | |  | |  | |  | |  | |  | |  |  |  |
| BRAF | chr7:140753337-140753337 | V600M | C>T | Single Nucleotide Variants (SNVs) | COSMIC(Lung,CRC) | No | |  | |  | |  | |  | |  | |  | |  | | | |  | |  | |  | |  | |  | |  | |  | |  | |  | |  | |  | |  | |  | |  | |  | |  | |  | |  | |  | |  | |  | |  | |  | |  | |  | |  |  |  |
| BRAF | chr7:140753339-140753339 | T599R | G>C | Single Nucleotide Variants (SNVs) | COSMIC(Lung) | No | |  | |  | |  | |  | |  | |  | |  | | | |  | |  | |  | |  | |  | |  | |  | |  | |  | |  | |  | |  | |  | |  | |  | |  | |  | |  | |  | |  | |  | |  | |  | |  | |  | |  |  |  |
| BRAF | chr7:140753344-140753344 | L597S | TAG>ACT | Multi- nucleotide variants (MNVs) | COSMIC(Lung) | No | |  | |  | |  | |  | |  | |  | |  | | | |  | |  | |  | |  | |  | |  | |  | |  | |  | |  | |  | |  | |  | |  | |  | |  | |  | |  | |  | |  | |  | |  | |  | |  | |  | |  |  |  |
| BRAF | chr7:140753344-140753344 | L597S | TAG>GCT | Multi- nucleotide variants (MNVs) | COSMIC(Lung) | No | |  | |  | |  | |  | |  | |  | |  | | | |  | |  | |  | |  | |  | |  | |  | |  | |  | |  | |  | |  | |  | |  | |  | |  | |  | |  | |  | |  | |  | |  | |  | |  | |  | |  |  |  |
| BRAF | chr7:140753345-140753345 | L597Q | A>T | Single Nucleotide Variants (SNVs) | COSMIC(Lung) | No | |  | |  | |  | |  | |  | |  | |  | | | |  | |  | |  | |  | |  | |  | |  | |  | |  | |  | |  | |  | |  | |  | |  | |  | |  | |  | |  | |  | |  | |  | |  | |  | |  | |  |  |  |
| BRAF | chr7:140753345-140753345 | L597R | A>C | Single Nucleotide Variants (SNVs) | COSMIC(Lung) | No | |  | |  | |  | |  | |  | |  | |  | | | |  | |  | |  | |  | |  | |  | |  | |  | |  | |  | |  | |  | |  | |  | |  | |  | |  | |  | |  | |  | |  | |  | |  | |  | |  | |  |  |  |
| BRAF | chr7:140753345-140753345 | L597S | AG>GA | Multi- nucleotide variants (MNVs) | COSMIC(Lung) | No | |  | |  | |  | |  | |  | |  | |  | | | |  | |  | |  | |  | |  | |  | |  | |  | |  | |  | |  | |  | |  | |  | |  | |  | |  | |  | |  | |  | |  | |  | |  | |  | |  | |  |  |  |
| BRAF | chr7:140753346-140753346 | L597V | G>C | Single Nucleotide Variants (SNVs) | COSMIC(Lung) | No | |  | |  | |  | |  | |  | |  | |  | | | |  | |  | |  | |  | |  | |  | |  | |  | |  | |  | |  | |  | |  | |  | |  | |  | |  | |  | |  | |  | |  | |  | |  | |  | |  | |  |  |  |
| BRAF | chr7:140753353-140753353 | D594E | A>C | Single Nucleotide Variants (SNVs) | COSMIC(Lung) | No | |  | |  | |  | |  | |  | |  | |  | | | |  | |  | |  | |  | |  | |  | |  | |  | |  | |  | |  | |  | |  | |  | |  | |  | |  | |  | |  | |  | |  | |  | |  | |  | |  | |  |  |  |
| BRAF | chr7:140753353-140753353 | D594E | A>T | Single Nucleotide Variants (SNVs) | COSMIC(Lung) | No | |  | |  | |  | |  | |  | |  | |  | | | |  | |  | |  | |  | |  | |  | |  | |  | |  | |  | |  | |  | |  | |  | |  | |  | |  | |  | |  | |  | |  | |  | |  | |  | |  | |  |  |  |
| BRAF | chr7:140753354-140753354 | D594A | T>G | Single Nucleotide Variants (SNVs) | COSMIC(Lung) | No | |  | |  | |  | |  | |  | |  | |  | | | |  | |  | |  | |  | |  | |  | |  | |  | |  | |  | |  | |  | |  | |  | |  | |  | |  | |  | |  | |  | |  | |  | |  | |  | |  | |  |  |  |
| BRAF | chr7:140753354-140753354 | D594G | T>C | Single Nucleotide Variants (SNVs) | COSMIC(Lung)-Tissue(CRC) | No | |  | |  | |  | |  | |  | |  | |  | | | |  | |  | |  | |  | |  | |  | |  | |  | |  | |  | |  | |  | |  | |  | |  | |  | |  | |  | |  | |  | |  | |  | |  | |  | |  | |  |  |  |
| BRAF | chr7:140753354-140753354 | D594V | T>A | Single Nucleotide Variants (SNVs) | COSMIC(Lung) | No | |  | |  | |  | |  | |  | |  | |  | | | |  | |  | |  | |  | |  | |  | |  | |  | |  | |  | |  | |  | |  | |  | |  | |  | |  | |  | |  | |  | |  | |  | |  | |  | |  | |  |  |  |
| BRAF | chr7:140753355-140753355 | D594H | C>G | Single Nucleotide Variants (SNVs) | COSMIC(Lung) | No | |  | |  | |  | |  | |  | |  | |  | | | |  | |  | |  | |  | |  | |  | |  | |  | |  | |  | |  | |  | |  | |  | |  | |  | |  | |  | |  | |  | |  | |  | |  | |  | |  | |  |  |  |
| BRAF | chr7:140753355-140753355 | D594N | C>T | Single Nucleotide Variants (SNVs) | COSMIC(Lung) | No | |  | |  | |  | |  | |  | |  | |  | | | |  | |  | |  | |  | |  | |  | |  | |  | |  | |  | |  | |  | |  | |  | |  | |  | |  | |  | |  | |  | |  | |  | |  | |  | |  | |  |  |  |
| BRAF | chr7:140753355-140753355 | D594Y | C>A | Single Nucleotide Variants (SNVs) | COSMIC(Lung) | No | |  | |  | |  | |  | |  | |  | |  | | | |  | |  | |  | |  | |  | |  | |  | |  | |  | |  | |  | |  | |  | |  | |  | |  | |  | |  | |  | |  | |  | |  | |  | |  | |  | |  |  |  |
| BRAF | chr7:140781601-140781601 | G469S | TCC>ACT | Multi- nucleotide variants (MNVs) | COSMIC(Lung,CRC) | No | |  | |  | |  | |  | |  | |  | |  | | | |  | |  | |  | |  | |  | |  | |  | |  | |  | |  | |  | |  | |  | |  | |  | |  | |  | |  | |  | |  | |  | |  | |  | |  | |  | |  |  |  |
| BRAF | chr7:140781601-140781601 | G469S | TCC>GCT | Multi- nucleotide variants (MNVs) | COSMIC(Lung,CRC) | No | |  | |  | |  | |  | |  | |  | |  | | | |  | |  | |  | |  | |  | |  | |  | |  | |  | |  | |  | |  | |  | |  | |  | |  | |  | |  | |  | |  | |  | |  | |  | |  | |  | |  |  |  |
| BRAF | chr7:140781602-140781602 | G469A | C>G | Single Nucleotide Variants (SNVs) | COSMIC(Lung,CRC,Gastric) | No | |  | |  | |  | |  | |  | |  | |  | | | |  | |  | |  | |  | |  | |  | |  | |  | |  | |  | |  | |  | |  | |  | |  | |  | |  | |  | |  | |  | |  | |  | |  | |  | |  | |  |  |  |
| BRAF | chr7:140781602-140781602 | G469E | C>T | Single Nucleotide Variants (SNVs) | COSMIC(Lung,CRC) | No | |  | |  | |  | |  | |  | |  | |  | | | |  | |  | |  | |  | |  | |  | |  | |  | |  | |  | |  | |  | |  | |  | |  | |  | |  | |  | |  | |  | |  | |  | |  | |  | |  | |  |  |  |
| BRAF | chr7:140781602-140781602 | G469L | CC>AA | Multi- nucleotide variants (MNVs) | COSMIC(Lung,CRC) | No | |  | |  | |  | |  | |  | |  | |  | | | |  | |  | |  | |  | |  | |  | |  | |  | |  | |  | |  | |  | |  | |  | |  | |  | |  | |  | |  | |  | |  | |  | |  | |  | |  | |  |  |  |
| BRAF | chr7:140781602-140781602 | G469L | CC>AG | Multi- nucleotide variants (MNVs) | COSMIC(Lung,CRC) | No | |  | |  | |  | |  | |  | |  | |  | | | |  | |  | |  | |  | |  | |  | |  | |  | |  | |  | |  | |  | |  | |  | |  | |  | |  | |  | |  | |  | |  | |  | |  | |  | |  | |  |  |  |
| BRAF | chr7:140781602-140781602 | G469S | CC>GA | Multi- nucleotide variants (MNVs) | COSMIC(Lung,CRC) | No | |  | |  | |  | |  | |  | |  | |  | | | |  | |  | |  | |  | |  | |  | |  | |  | |  | |  | |  | |  | |  | |  | |  | |  | |  | |  | |  | |  | |  | |  | |  | |  | |  | |  |  |  |
| BRAF | chr7:140781602-140781602 | G469V | C>A | Single Nucleotide Variants (SNVs) | COSMIC(Lung,CRC,Gastric) | No | |  | |  | |  | |  | |  | |  | |  | | | |  | |  | |  | |  | |  | |  | |  | |  | |  | |  | |  | |  | |  | |  | |  | |  | |  | |  | |  | |  | |  | |  | |  | |  | |  | |  |  |  |
| BRAF | chr7:140781603-140781603 | G469R | C>G | Single Nucleotide Variants (SNVs) | COSMIC(Lung,CRC,Gastric) | No | |  | |  | |  | |  | |  | |  | |  | | | |  | |  | |  | |  | |  | |  | |  | |  | |  | |  | |  | |  | |  | |  | |  | |  | |  | |  | |  | |  | |  | |  | |  | |  | |  | |  |  |  |
| BRAF | chr7:140781603-140781603 | G469R | C>T | Single Nucleotide Variants (SNVs) | COSMIC(Lung,CRC,Gastric) | No | |  | |  | |  | |  | |  | |  | |  | | | |  | |  | |  | |  | |  | |  | |  | |  | |  | |  | |  | |  | |  | |  | |  | |  | |  | |  | |  | |  | |  | |  | |  | |  | |  | |  |  |  |
| BRAF | chr7:140781608-140781608 | S467L | G>A | Single Nucleotide Variants (SNVs) | COSMIC(Lung) | No | |  | |  | |  | |  | |  | |  | |  | | | |  | |  | |  | |  | |  | |  | |  | |  | |  | |  | |  | |  | |  | |  | |  | |  | |  | |  | |  | |  | |  | |  | |  | |  | |  | |  |  |  |
| BRAF | chr7:140781609-140781609 | G466Del | ATCC>A | Deletion | COSMIC(HCC) | No | |  | |  | |  | |  | |  | |  | |  | | | |  | |  | |  | |  | |  | |  | |  | |  | |  | |  | |  | |  | |  | |  | |  | |  | |  | |  | |  | |  | |  | |  | |  | |  | |  | |  |  |  |
| BRAF | chr7:140781610-140781610 | G466D | TC>AT | Multi- nucleotide variants (MNVs) | COSMIC(HCC) | No | |  | |  | |  | |  | |  | |  | |  | | | |  | |  | |  | |  | |  | |  | |  | |  | |  | |  | |  | |  | |  | |  | |  | |  | |  | |  | |  | |  | |  | |  | |  | |  | |  | |  |  |  |
| BRAF | chr7:140781610-140781610 | G466D | TC>GT | Multi- nucleotide variants (MNVs) | COSMIC(HCC) | No | |  | |  | |  | |  | |  | |  | |  | | | |  | |  | |  | |  | |  | |  | |  | |  | |  | |  | |  | |  | |  | |  | |  | |  | |  | |  | |  | |  | |  | |  | |  | |  | |  | |  |  |  |
| BRAF | chr7:140781610-140781610 | G466dup | T>TACC | Insertion | COSMIC(HCC) | No | |  | |  | |  | |  | |  | |  | |  | | | |  | |  | |  | |  | |  | |  | |  | |  | |  | |  | |  | |  | |  | |  | |  | |  | |  | |  | |  | |  | |  | |  | |  | |  | |  | |  |  |  |
| BRAF | chr7:140781610-140781610 | G466dup | T>TCCC | Insertion | COSMIC(HCC) | No | |  | |  | |  | |  | |  | |  | |  | | | |  | |  | |  | |  | |  | |  | |  | |  | |  | |  | |  | |  | |  | |  | |  | |  | |  | |  | |  | |  | |  | |  | |  | |  | |  | |  |  |  |
| BRAF | chr7:140781610-140781610 | G466dup | T>TGCC | Insertion | COSMIC(HCC) | No | |  | |  | |  | |  | |  | |  | |  | | | |  | |  | |  | |  | |  | |  | |  | |  | |  | |  | |  | |  | |  | |  | |  | |  | |  | |  | |  | |  | |  | |  | |  | |  | |  | |  |  |  |
| BRAF | chr7:140781610-140781610 | G466dup | T>TTCC | Insertion | COSMIC(HCC) | No | |  | |  | |  | |  | |  | |  | |  | | | |  | |  | |  | |  | |  | |  | |  | |  | |  | |  | |  | |  | |  | |  | |  | |  | |  | |  | |  | |  | |  | |  | |  | |  | |  | |  |  |  |
| BRAF | chr7:140781611-140781611 | G466A | C>G | Single Nucleotide Variants (SNVs) | COSMIC(Lung,CRC,HCC) | No | |  | |  | |  | |  | |  | |  | |  | | | |  | |  | |  | |  | |  | |  | |  | |  | |  | |  | |  | |  | |  | |  | |  | |  | |  | |  | |  | |  | |  | |  | |  | |  | |  | |  |  |  |
| BRAF | chr7:140781611-140781611 | G466E | C>T | Single Nucleotide Variants (SNVs) | COSMIC(Lung,CRC,HCC) | No | |  | |  | |  | |  | |  | |  | |  | | | |  | |  | |  | |  | |  | |  | |  | |  | |  | |  | |  | |  | |  | |  | |  | |  | |  | |  | |  | |  | |  | |  | |  | |  | |  | |  |  |  |
| BRAF | chr7:140781611-140781611 | G466V | C>A | Single Nucleotide Variants (SNVs) | COSMIC(Lung,CRC,HCC) | No | |  | |  | |  | |  | |  | |  | |  | | | |  | |  | |  | |  | |  | |  | |  | |  | |  | |  | |  | |  | |  | |  | |  | |  | |  | |  | |  | |  | |  | |  | |  | |  | |  | |  |  |  |
| BRAF | chr7:140781612-140781612 | G466R | C>G | Single Nucleotide Variants (SNVs) | COSMIC(Lung,CRC,HCC) | No | |  | |  | |  | |  | |  | |  | |  | | | |  | |  | |  | |  | |  | |  | |  | |  | |  | |  | |  | |  | |  | |  | |  | |  | |  | |  | |  | |  | |  | |  | |  | |  | |  | |  |  |  |
| BRAF | chr7:140781612-140781612 | G466R | C>T | Single Nucleotide Variants (SNVs) | COSMIC(Lung,CRC,HCC) | No | |  | |  | |  | |  | |  | |  | |  | | | |  | |  | |  | |  | |  | |  | |  | |  | |  | |  | |  | |  | |  | |  | |  | |  | |  | |  | |  | |  | |  | |  | |  | |  | |  | |  |  |  |
| BRAF | chr7:140781615-140781615 | G464Del | ATCC>A | Deletion | COSMIC(Gastric) | No | |  | |  | |  | |  | |  | |  | |  | | | |  | |  | |  | |  | |  | |  | |  | |  | |  | |  | |  | |  | |  | |  | |  | |  | |  | |  | |  | |  | |  | |  | |  | |  | |  | |  |  |  |
| BRAF | chr7:140781616-140781616 | G464dup | T>TACC | Insertion | COSMIC(Gastric) | No | |  | |  | |  | |  | |  | |  | |  | | | |  | |  | |  | |  | |  | |  | |  | |  | |  | |  | |  | |  | |  | |  | |  | |  | |  | |  | |  | |  | |  | |  | |  | |  | |  | |  |  |  |
| BRAF | chr7:140781616-140781616 | G464dup | T>TCCC | Insertion | COSMIC(Gastric) | No | |  | |  | |  | |  | |  | |  | |  | | | |  | |  | |  | |  | |  | |  | |  | |  | |  | |  | |  | |  | |  | |  | |  | |  | |  | |  | |  | |  | |  | |  | |  | |  | |  | |  |  |  |
| BRAF | chr7:140781616-140781616 | G464dup | T>TGCC | Insertion | COSMIC(Gastric) | No | |  | |  | |  | |  | |  | |  | |  | | | |  | |  | |  | |  | |  | |  | |  | |  | |  | |  | |  | |  | |  | |  | |  | |  | |  | |  | |  | |  | |  | |  | |  | |  | |  | |  |  |  |
| BRAF | chr7:140781616-140781616 | G464dup | T>TTCC | Insertion | COSMIC(Gastric) | No | |  | |  | |  | |  | |  | |  | |  | | | |  | |  | |  | |  | |  | |  | |  | |  | |  | |  | |  | |  | |  | |  | |  | |  | |  | |  | |  | |  | |  | |  | |  | |  | |  | |  |  |  |
| BRAF | chr7:140781617-140781617 | G464E | C>T | Single Nucleotide Variants (SNVs) | COSMIC(Gastric) | No | |  | |  | |  | |  | |  | |  | |  | | | |  | |  | |  | |  | |  | |  | |  | |  | |  | |  | |  | |  | |  | |  | |  | |  | |  | |  | |  | |  | |  | |  | |  | |  | |  | |  |  |  |
| BRAF | chr7:140781617-140781617 | G464V | C>A | Single Nucleotide Variants (SNVs) | COSMIC(Lung,Gastric) | No | |  | |  | |  | |  | |  | |  | |  | | | |  | |  | |  | |  | |  | |  | |  | |  | |  | |  | |  | |  | |  | |  | |  | |  | |  | |  | |  | |  | |  | |  | |  | |  | |  | |  |  |  |
| BRAF | chr7:140781618-140781618 | G464R | C>G | Single Nucleotide Variants (SNVs) | COSMIC(Gastric) | No | |  | |  | |  | |  | |  | |  | |  | | | |  | |  | |  | |  | |  | |  | |  | |  | |  | |  | |  | |  | |  | |  | |  | |  | |  | |  | |  | |  | |  | |  | |  | |  | |  | |  |  |  |
| BRAF | chr7:140781618-140781618 | G464R | C>T | Single Nucleotide Variants (SNVs) | COSMIC(Gastric) | No | |  | |  | |  | |  | |  | |  | |  | | | |  | |  | |  | |  | |  | |  | |  | |  | |  | |  | |  | |  | |  | |  | |  | |  | |  | |  | |  | |  | |  | |  | |  | |  | |  | |  |  |  |
| CTNNB1 | chr3:41224606-41224606 | D32H | G>C | Single Nucleotide Variants (SNVs) | COSMIC(Breast,Gastric,HCC) | No | |  | |  | |  | |  | |  | |  | |  | | | |  | |  | |  | |  | |  | |  | |  | |  | |  | |  | |  | |  | |  | |  | |  | |  | |  | |  | |  | |  | |  | |  | |  | |  | |  | |  |  |  |
| CTNNB1 | chr3:41224606-41224606 | D32N | G>A | Single Nucleotide Variants (SNVs) | COSMIC(Breast,HCC) | No | |  | |  | |  | |  | |  | |  | |  | | | |  | |  | |  | |  | |  | |  | |  | |  | |  | |  | |  | |  | |  | |  | |  | |  | |  | |  | |  | |  | |  | |  | |  | |  | |  | |  |  |  |
| CTNNB1 | chr3:41224606-41224606 | D32Y | G>T | Single Nucleotide Variants (SNVs) | COSMIC(Breast,HCC) | No | |  | |  | |  | |  | |  | |  | |  | | | |  | |  | |  | |  | |  | |  | |  | |  | |  | |  | |  | |  | |  | |  | |  | |  | |  | |  | |  | |  | |  | |  | |  | |  | |  | |  |  |  |
| CTNNB1 | chr3:41224607-41224607 | D32A | A>C | Single Nucleotide Variants (SNVs) | COSMIC(Breast,HCC) | No | |  | |  | |  | |  | |  | |  | |  | | | |  | |  | |  | |  | |  | |  | |  | |  | |  | |  | |  | |  | |  | |  | |  | |  | |  | |  | |  | |  | |  | |  | |  | |  | |  | |  |  |  |
| CTNNB1 | chr3:41224607-41224607 | D32G | A>G | Single Nucleotide Variants (SNVs) | Tissue(HCC)-COSMIC(Breast,HCC) | No | |  | |  | |  | |  | |  | |  | |  | | | |  | |  | |  | |  | |  | |  | |  | |  | |  | |  | |  | |  | |  | |  | |  | |  | |  | |  | |  | |  | |  | |  | |  | |  | |  | |  |  |  |
| CTNNB1 | chr3:41224607-41224607 | D32V | A>T | Single Nucleotide Variants (SNVs) | COSMIC(Breast,HCC) | No | |  | |  | |  | |  | |  | |  | |  | | | |  | |  | |  | |  | |  | |  | |  | |  | |  | |  | |  | |  | |  | |  | |  | |  | |  | |  | |  | |  | |  | |  | |  | |  | |  | |  |  |  |
| CTNNB1 | chr3:41224609-41224609 | S33A | T>G | Single Nucleotide Variants (SNVs) | COSMIC(Breast,HCC) | No | |  | |  | |  | |  | |  | |  | |  | | | |  | |  | |  | |  | |  | |  | |  | |  | |  | |  | |  | |  | |  | |  | |  | |  | |  | |  | |  | |  | |  | |  | |  | |  | |  | |  |  |  |
| CTNNB1 | chr3:41224609-41224609 | S33P | T>C | Single Nucleotide Variants (SNVs) | Tissue(HCC)-COSMIC(Breast,HCC) | No | |  | |  | |  | |  | |  | |  | |  | | | |  | |  | |  | |  | |  | |  | |  | |  | |  | |  | |  | |  | |  | |  | |  | |  | |  | |  | |  | |  | |  | |  | |  | |  | |  | |  |  |  |
| CTNNB1 | chr3:41224610-41224610 | S33C | C>G | Single Nucleotide Variants (SNVs) | COSMIC(Breast,HCC) | No | |  | |  | |  | |  | |  | |  | |  | | | |  | |  | |  | |  | |  | |  | |  | |  | |  | |  | |  | |  | |  | |  | |  | |  | |  | |  | |  | |  | |  | |  | |  | |  | |  | |  |  |  |
| CTNNB1 | chr3:41224610-41224610 | S33F | C>T | Single Nucleotide Variants (SNVs) | Tissue(HCC)-COSMIC(Breast,HCC) | No | |  | |  | |  | |  | |  | |  | |  | | | |  | |  | |  | |  | |  | |  | |  | |  | |  | |  | |  | |  | |  | |  | |  | |  | |  | |  | |  | |  | |  | |  | |  | |  | |  | |  |  |  |
| CTNNB1 | chr3:41224610-41224610 | S33Y | C>A | Single Nucleotide Variants (SNVs) | COSMIC(Breast,HCC) | No | |  | |  | |  | |  | |  | |  | |  | | | |  | |  | |  | |  | |  | |  | |  | |  | |  | |  | |  | |  | |  | |  | |  | |  | |  | |  | |  | |  | |  | |  | |  | |  | |  | |  |  |  |
| CTNNB1 | chr3:41224612-41224612 | G34R | G>A | Single Nucleotide Variants (SNVs) | COSMIC(Breast,HCC) | No | |  | |  | |  | |  | |  | |  | |  | | | |  | |  | |  | |  | |  | |  | |  | |  | |  | |  | |  | |  | |  | |  | |  | |  | |  | |  | |  | |  | |  | |  | |  | |  | |  | |  |  |  |
| CTNNB1 | chr3:41224612-41224612 | G34R | G>C | Single Nucleotide Variants (SNVs) | COSMIC(Breast,HCC) | No | |  | |  | |  | |  | |  | |  | |  | | | |  | |  | |  | |  | |  | |  | |  | |  | |  | |  | |  | |  | |  | |  | |  | |  | |  | |  | |  | |  | |  | |  | |  | |  | |  | |  |  |  |
| CTNNB1 | chr3:41224613-41224613 | G34E | G>A | Single Nucleotide Variants (SNVs) | COSMIC(Breast,HCC) | No | |  | |  | |  | |  | |  | |  | |  | | | |  | |  | |  | |  | |  | |  | |  | |  | |  | |  | |  | |  | |  | |  | |  | |  | |  | |  | |  | |  | |  | |  | |  | |  | |  | |  |  |  |
| CTNNB1 | chr3:41224613-41224613 | G34V | G>T | Single Nucleotide Variants (SNVs) | Tissue(HCC)-COSMIC(Breast,HCC) | No | |  | |  | |  | |  | |  | |  | |  | | | |  | |  | |  | |  | |  | |  | |  | |  | |  | |  | |  | |  | |  | |  | |  | |  | |  | |  | |  | |  | |  | |  | |  | |  | |  | |  |  |  |
| CTNNB1 | chr3:41224616-41224616 | I35S | T>G | Single Nucleotide Variants (SNVs) | COSMIC(Breast,HCC) | No | |  | |  | |  | |  | |  | |  | |  | | | |  | |  | |  | |  | |  | |  | |  | |  | |  | |  | |  | |  | |  | |  | |  | |  | |  | |  | |  | |  | |  | |  | |  | |  | |  | |  |  |  |
| CTNNB1 | chr3:41224619-41224619 | H36P | A>C | Single Nucleotide Variants (SNVs) | COSMIC(HCC) | No | |  | |  | |  | |  | |  | |  | |  | | | |  | |  | |  | |  | |  | |  | |  | |  | |  | |  | |  | |  | |  | |  | |  | |  | |  | |  | |  | |  | |  | |  | |  | |  | |  | |  |  |  |
| CTNNB1 | chr3:41224621-41224621 | S37A | T>G | Single Nucleotide Variants (SNVs) | COSMIC(Breast,HCC) | No | |  | |  | |  | |  | |  | |  | |  | | | |  | |  | |  | |  | |  | |  | |  | |  | |  | |  | |  | |  | |  | |  | |  | |  | |  | |  | |  | |  | |  | |  | |  | |  | |  | |  |  |  |
| CTNNB1 | chr3:41224621-41224621 | S37P | T>C | Single Nucleotide Variants (SNVs) | COSMIC(Breast,HCC) | No | |  | |  | |  | |  | |  | |  | |  | | | |  | |  | |  | |  | |  | |  | |  | |  | |  | |  | |  | |  | |  | |  | |  | |  | |  | |  | |  | |  | |  | |  | |  | |  | |  | |  |  |  |
| CTNNB1 | chr3:41224622-41224622 | S37C | C>G | Single Nucleotide Variants (SNVs) | COSMIC(Breast,HCC) | No | |  | |  | |  | |  | |  | |  | |  | | | |  | |  | |  | |  | |  | |  | |  | |  | |  | |  | |  | |  | |  | |  | |  | |  | |  | |  | |  | |  | |  | |  | |  | |  | |  | |  |  |  |
| CTNNB1 | chr3:41224622-41224622 | S37F | C>T | Single Nucleotide Variants (SNVs) | Tissue(Breast)-COSMIC(Breast,Gastric,HCC) | No | |  | |  | |  | |  | |  | |  | |  | | | |  | |  | |  | |  | |  | |  | |  | |  | |  | |  | |  | |  | |  | |  | |  | |  | |  | |  | |  | |  | |  | |  | |  | |  | |  | |  |  |  |
| CTNNB1 | chr3:41224622-41224622 | S37Y | C>A | Single Nucleotide Variants (SNVs) | COSMIC(Breast,HCC) | No | |  | |  | |  | |  | |  | |  | |  | | | |  | |  | |  | |  | |  | |  | |  | |  | |  | |  | |  | |  | |  | |  | |  | |  | |  | |  | |  | |  | |  | |  | |  | |  | |  | |  |  |  |
| CTNNB1 | chr3:41224633-41224633 | T41A | A>G | Single Nucleotide Variants (SNVs) | Tissue(CRC)-COSMIC(CRC,Breast,HCC) | No | |  | |  | |  | |  | |  | |  | |  | | | |  | |  | |  | |  | |  | |  | |  | |  | |  | |  | |  | |  | |  | |  | |  | |  | |  | |  | |  | |  | |  | |  | |  | |  | |  | |  |  |  |
| CTNNB1 | chr3:41224634-41224634 | T41I | C>T | Single Nucleotide Variants (SNVs) | Tissue(HCC)-COSMIC(CRC,Breast,HCC) | No | |  | |  | |  | |  | |  | |  | |  | | | |  | |  | |  | |  | |  | |  | |  | |  | |  | |  | |  | |  | |  | |  | |  | |  | |  | |  | |  | |  | |  | |  | |  | |  | |  | |  |  |  |
| CTNNB1 | chr3:41224644-41224644 | S45Del | TTCT>T | Deletion | COSMIC(CRC) | No | |  | |  | |  | |  | |  | |  | |  | | | |  | |  | |  | |  | |  | |  | |  | |  | |  | |  | |  | |  | |  | |  | |  | |  | |  | |  | |  | |  | |  | |  | |  | |  | |  | |  |  |  |
| CTNNB1 | chr3:41224645-41224645 | S45A | T>G | Single Nucleotide Variants (SNVs) | Tissue(CRC)-COSMIC(HCC) | No | |  | |  | |  | |  | |  | |  | |  | | | |  | |  | |  | |  | |  | |  | |  | |  | |  | |  | |  | |  | |  | |  | |  | |  | |  | |  | |  | |  | |  | |  | |  | |  | |  | |  |  |  |
| CTNNB1 | chr3:41224645-41224645 | S45P | T>C | Single Nucleotide Variants (SNVs) | COSMIC(HCC,CRC)-Tissue(CRC,HCC) | No | |  | |  | |  | |  | |  | |  | |  | | | |  | |  | |  | |  | |  | |  | |  | |  | |  | |  | |  | |  | |  | |  | |  | |  | |  | |  | |  | |  | |  | |  | |  | |  | |  | |  |  |  |
| CTNNB1 | chr3:41224646-41224646 | S45C | C>G | Single Nucleotide Variants (SNVs) | COSMIC(Breast,HCC) | No | |  | |  | |  | |  | |  | |  | |  | | | |  | |  | |  | |  | |  | |  | |  | |  | |  | |  | |  | |  | |  | |  | |  | |  | |  | |  | |  | |  | |  | |  | |  | |  | |  | |  |  |  |
| CTNNB1 | chr3:41224646-41224646 | S45F | C>T | Single Nucleotide Variants (SNVs) | Tissue(HCC)-COSMIC(CRC,HCC) | No | |  | |  | |  | |  | |  | |  | |  | | | |  | |  | |  | |  | |  | |  | |  | |  | |  | |  | |  | |  | |  | |  | |  | |  | |  | |  | |  | |  | |  | |  | |  | |  | |  | |  |  |  |
| CTNNB1 | chr3:41224646-41224646 | S45Y | C>A | Single Nucleotide Variants (SNVs) | Tissue(HCC)-COSMIC(HCC) | No | |  | |  | |  | |  | |  | |  | |  | | | |  | |  | |  | |  | |  | |  | |  | |  | |  | |  | |  | |  | |  | |  | |  | |  | |  | |  | |  | |  | |  | |  | |  | |  | |  | |  |  |  |
| EGFR | chr7:55173984-55173984 | E709K | G>A | Single Nucleotide Variants (SNVs) | COSMIC(Lung) | No | |  | |  | |  | |  | |  | |  | |  | | | |  | |  | |  | |  | |  | |  | |  | |  | |  | |  | |  | |  | |  | |  | |  | |  | |  | |  | |  | |  | |  | |  | |  | |  | |  | |  |  |  |
| EGFR | chr7:55173984-55173984 | E709Q | G>C | Single Nucleotide Variants (SNVs) | COSMIC(Lung) | No | |  | |  | |  | |  | |  | |  | |  | | | |  | |  | |  | |  | |  | |  | |  | |  | |  | |  | |  | |  | |  | |  | |  | |  | |  | |  | |  | |  | |  | |  | |  | |  | |  | |  |  |  |
| EGFR | chr7:55173985-55173985 | E709A | A>C | Single Nucleotide Variants (SNVs) | COSMIC(Lung) | No | |  | |  | |  | |  | |  | |  | |  | | | |  | |  | |  | |  | |  | |  | |  | |  | |  | |  | |  | |  | |  | |  | |  | |  | |  | |  | |  | |  | |  | |  | |  | |  | |  | |  |  |  |
| EGFR | chr7:55173985-55173985 | E709G | A>G | Single Nucleotide Variants (SNVs) | COSMIC(Lung) | No | |  | |  | |  | |  | |  | |  | |  | | | |  | |  | |  | |  | |  | |  | |  | |  | |  | |  | |  | |  | |  | |  | |  | |  | |  | |  | |  | |  | |  | |  | |  | |  | |  | |  |  |  |
| EGFR | chr7:55173985-55173985 | E709V | A>T | Single Nucleotide Variants (SNVs) | COSMIC(Lung) | No | |  | |  | |  | |  | |  | |  | |  | | | |  | |  | |  | |  | |  | |  | |  | |  | |  | |  | |  | |  | |  | |  | |  | |  | |  | |  | |  | |  | |  | |  | |  | |  | |  | |  |  |  |
| EGFR | chr7:55174011-55174011 | L718V | C>G | Single Nucleotide Variants (SNVs) | COSMIC(Lung) | No | |  | |  | |  | |  | |  | |  | |  | | | |  | |  | |  | |  | |  | |  | |  | |  | |  | |  | |  | |  | |  | |  | |  | |  | |  | |  | |  | |  | |  | |  | |  | |  | |  | |  |  |  |
| EGFR | chr7:55174012-55174012 | L718Q | T>A | Single Nucleotide Variants (SNVs) | COSMIC(Lung) | No | |  | |  | |  | |  | |  | |  | |  | | | |  | |  | |  | |  | |  | |  | |  | |  | |  | |  | |  | |  | |  | |  | |  | |  | |  | |  | |  | |  | |  | |  | |  | |  | |  | |  |  |  |
| EGFR | chr7:55174014-55174014 | G719C | G>T | Single Nucleotide Variants (SNVs) | COSMIC(Lung)-Tissue(Lung) | No | |  | |  | |  | |  | |  | |  | |  | | | |  | |  | |  | |  | |  | |  | |  | |  | |  | |  | |  | |  | |  | |  | |  | |  | |  | |  | |  | |  | |  | |  | |  | |  | |  | |  |  |  |
| EGFR | chr7:55174014-55174014 | G719R | G>C | Single Nucleotide Variants (SNVs) | COSMIC(Lung) | No | |  | |  | |  | |  | |  | |  | |  | | | |  | |  | |  | |  | |  | |  | |  | |  | |  | |  | |  | |  | |  | |  | |  | |  | |  | |  | |  | |  | |  | |  | |  | |  | |  | |  |  |  |
| EGFR | chr7:55174014-55174014 | G719R | GGC>AGA | Multi- nucleotide variants (MNVs) | COSMIC(Lung) | No | |  | |  | |  | |  | |  | |  | |  | | | |  | |  | |  | |  | |  | |  | |  | |  | |  | |  | |  | |  | |  | |  | |  | |  | |  | |  | |  | |  | |  | |  | |  | |  | |  | |  |  |  |
| EGFR | chr7:55174014-55174014 | G719R | GGC>AGG | Multi- nucleotide variants (MNVs) | COSMIC(Lung) | No | |  | |  | |  | |  | |  | |  | |  | | | |  | |  | |  | |  | |  | |  | |  | |  | |  | |  | |  | |  | |  | |  | |  | |  | |  | |  | |  | |  | |  | |  | |  | |  | |  | |  |  |  |
| EGFR | chr7:55174014-55174014 | G719S | G>A | Single Nucleotide Variants (SNVs) | COSMIC(Lung)-Tissue(Lung) | No | |  | |  | |  | |  | |  | |  | |  | | | |  | |  | |  | |  | |  | |  | |  | |  | |  | |  | |  | |  | |  | |  | |  | |  | |  | |  | |  | |  | |  | |  | |  | |  | |  | |  |  |  |
| EGFR | chr7:55174014-55174014 | G719S | GG>TC | Multi- nucleotide variants (MNVs) | COSMIC(Lung) | No | |  | |  | |  | |  | |  | |  | |  | | | |  | |  | |  | |  | |  | |  | |  | |  | |  | |  | |  | |  | |  | |  | |  | |  | |  | |  | |  | |  | |  | |  | |  | |  | |  | |  |  |  |
| EGFR | chr7:55174015-55174015 | G719A | G>C | Single Nucleotide Variants (SNVs) | COSMIC(Lung) | No | |  | |  | |  | |  | |  | |  | |  | | | |  | |  | |  | |  | |  | |  | |  | |  | |  | |  | |  | |  | |  | |  | |  | |  | |  | |  | |  | |  | |  | |  | |  | |  | |  | |  |  |  |
| EGFR | chr7:55174015-55174015 | G719D | G>A | Single Nucleotide Variants (SNVs) | COSMIC(Lung) | No | |  | |  | |  | |  | |  | |  | |  | | | |  | |  | |  | |  | |  | |  | |  | |  | |  | |  | |  | |  | |  | |  | |  | |  | |  | |  | |  | |  | |  | |  | |  | |  | |  | |  |  |  |
| EGFR | chr7:55174029-55174029 | G724S | G>A | Single Nucleotide Variants (SNVs) | COSMIC(Lung) | No | |  | |  | |  | |  | |  | |  | |  | | | |  | |  | |  | |  | |  | |  | |  | |  | |  | |  | |  | |  | |  | |  | |  | |  | |  | |  | |  | |  | |  | |  | |  | |  | |  | |  |  |  |
| EGFR | chr7:55174029-55174029 | G724S | GG>TC | Multi- nucleotide variants (MNVs) | COSMIC(Lung) | No | |  | |  | |  | |  | |  | |  | |  | | | |  | |  | |  | |  | |  | |  | |  | |  | |  | |  | |  | |  | |  | |  | |  | |  | |  | |  | |  | |  | |  | |  | |  | |  | |  | |  |  |  |
| EGFR | chr7:55174033-55174033 | T725M | C>T | Single Nucleotide Variants (SNVs) | COSMIC(Lung) | No | |  | |  | |  | |  | |  | |  | |  | | | |  | |  | |  | |  | |  | |  | |  | |  | |  | |  | |  | |  | |  | |  | |  | |  | |  | |  | |  | |  | |  | |  | |  | |  | |  | |  |  |  |
| EGFR | chr7:55174035-55174035 | V726M | G>A | Single Nucleotide Variants (SNVs) | COSMIC(Lung) | No | |  | |  | |  | |  | |  | |  | |  | | | |  | |  | |  | |  | |  | |  | |  | |  | |  | |  | |  | |  | |  | |  | |  | |  | |  | |  | |  | |  | |  | |  | |  | |  | |  | |  |  |  |
| EGFR | chr7:55174771-55174771 | E746_A750Del | AGGAATTAAGAGAAGC>A | Deletion | COSMIC(Lung)-Tissue(Lung) | Yes | |  | |  | |  | |  | |  | |  | |  | | | |  | |  | |  | |  | |  | |  | |  | |  | |  | |  | |  | |  | |  | |  | |  | |  | |  | |  | |  | |  | |  | |  | |  | |  | |  | |  |  |  |
| EGFR | chr7:55174771-55174771 | K745R | A>G | Single Nucleotide Variants (SNVs) | COSMIC(Lung) | No | |  | |  | |  | |  | |  | |  | |  | | | |  | |  | |  | |  | |  | |  | |  | |  | |  | |  | |  | |  | |  | |  | |  | |  | |  | |  | |  | |  | |  | |  | |  | |  | |  | |  |  |  |
| EGFR | chr7:55174772-55174772 | E746_A750Del | GGAATTAAGAGAAGCA>G | Deletion | COSMIC(Lung) | Yes | |  | |  | |  | |  | |  | |  | |  | | | |  | |  | |  | |  | |  | |  | |  | |  | |  | |  | |  | |  | |  | |  | |  | |  | |  | |  | |  | |  | |  | |  | |  | |  | |  | |  |  |  |
| EGFR | chr7:55174772-55174772 | L747_E749Del | GGAATTAAGA>G | Deletion | COSMIC(Lung) | No | |  | |  | |  | |  | |  | |  | |  | | | |  | |  | |  | |  | |  | |  | |  | |  | |  | |  | |  | |  | |  | |  | |  | |  | |  | |  | |  | |  | |  | |  | |  | |  | |  | |  |  |  |
| EGFR | chr7:55174773-55174773 | E746Q | G>C | Single Nucleotide Variants (SNVs) | COSMIC(Lung) | No | |  | |  | |  | |  | |  | |  | |  | | | |  | |  | |  | |  | |  | |  | |  | |  | |  | |  | |  | |  | |  | |  | |  | |  | |  | |  | |  | |  | |  | |  | |  | |  | |  | |  |  |  |
| EGFR | chr7:55174773-55174773 | E746Vfs*16 | GAA>G | Deletion | COSMIC(Lung) | No | |  | |  | |  | |  | |  | |  | |  | | | |  | |  | |  | |  | |  | |  | |  | |  | |  | |  | |  | |  | |  | |  | |  | |  | |  | |  | |  | |  | |  | |  | |  | |  | |  | |  |  |  |
| EGFR | chr7:55174774-55174774 | E746G | A>G | Single Nucleotide Variants (SNVs) | COSMIC(Lung) | No | |  | |  | |  | |  | |  | |  | |  | | | |  | |  | |  | |  | |  | |  | |  | |  | |  | |  | |  | |  | |  | |  | |  | |  | |  | |  | |  | |  | |  | |  | |  | |  | |  | |  |  |  |
| EGFR | chr7:55174774-55174774 | L747_T751Del | AATTAAGAGAAGCAAC>A | Deletion | Tissue(Lung) | No | |  | |  | |  | |  | |  | |  | |  | | | |  | |  | |  | |  | |  | |  | |  | |  | |  | |  | |  | |  | |  | |  | |  | |  | |  | |  | |  | |  | |  | |  | |  | |  | |  | |  |  |  |
| EGFR | chr7:55174775-55174775 | L747_S752Del | ATTAAGAGAAGCAACATCT>A | Deletion | COSMIC(Lung) | No | |  | |  | |  | |  | |  | |  | |  | | | |  | |  | |  | |  | |  | |  | |  | |  | |  | |  | |  | |  | |  | |  | |  | |  | |  | |  | |  | |  | |  | |  | |  | |  | |  | |  |  |  |
| EGFR | chr7:55174775-55174775 | L747Qfs*16 | ATTAAGAGAAG>A | Deletion | COSMIC(Lung) | No | |  | |  | |  | |  | |  | |  | |  | | | |  | |  | |  | |  | |  | |  | |  | |  | |  | |  | |  | |  | |  | |  | |  | |  | |  | |  | |  | |  | |  | |  | |  | |  | |  | |  |  |  |
| EGFR | chr7:55174776-55174776 | L747Ffs*14 | TTAAGAGAAGCAACATC>T | Deletion | COSMIC(Lung) | No | |  | |  | |  | |  | |  | |  | |  | | | |  | |  | |  | |  | |  | |  | |  | |  | |  | |  | |  | |  | |  | |  | |  | |  | |  | |  | |  | |  | |  | |  | |  | |  | |  | |  |  |  |
| EGFR | chr7:55174776-55174776 | L747P | TT>CC | Multi- nucleotide variants (MNVs) | COSMIC(Lung) | No | |  | |  | |  | |  | |  | |  | |  | | | |  | |  | |  | |  | |  | |  | |  | |  | |  | |  | |  | |  | |  | |  | |  | |  | |  | |  | |  | |  | |  | |  | |  | |  | |  | |  |  |  |
| EGFR | chr7:55174776-55174776 | L747S | TTA>AGC | Multi- nucleotide variants (MNVs) | COSMIC(Lung) | No | |  | |  | |  | |  | |  | |  | |  | | | |  | |  | |  | |  | |  | |  | |  | |  | |  | |  | |  | |  | |  | |  | |  | |  | |  | |  | |  | |  | |  | |  | |  | |  | |  | |  |  |  |
| EGFR | chr7:55174776-55174776 | L747S | TTA>AGT | Multi- nucleotide variants (MNVs) | COSMIC(Lung) | No | |  | |  | |  | |  | |  | |  | |  | | | |  | |  | |  | |  | |  | |  | |  | |  | |  | |  | |  | |  | |  | |  | |  | |  | |  | |  | |  | |  | |  | |  | |  | |  | |  | |  |  |  |
| EGFR | chr7:55174776-55174776 | L747V | T>G | Single Nucleotide Variants (SNVs) | COSMIC(Lung) | No | |  | |  | |  | |  | |  | |  | |  | | | |  | |  | |  | |  | |  | |  | |  | |  | |  | |  | |  | |  | |  | |  | |  | |  | |  | |  | |  | |  | |  | |  | |  | |  | |  | |  |  |  |
| EGFR | chr7:55174777-55174777 | L747S | T>C | Single Nucleotide Variants (SNVs) | COSMIC(Lung) | No | |  | |  | |  | |  | |  | |  | |  | | | |  | |  | |  | |  | |  | |  | |  | |  | |  | |  | |  | |  | |  | |  | |  | |  | |  | |  | |  | |  | |  | |  | |  | |  | |  | |  |  |  |
| EGFR | chr7:55174785-55174785 | A750P | G>C | Single Nucleotide Variants (SNVs) | COSMIC(Lung) | No | |  | |  | |  | |  | |  | |  | |  | | | |  | |  | |  | |  | |  | |  | |  | |  | |  | |  | |  | |  | |  | |  | |  | |  | |  | |  | |  | |  | |  | |  | |  | |  | |  | |  |  |  |
| EGFR | chr7:55174790-55174790 | P753Efs*9 | ATC>A | Deletion | COSMIC(Lung) | No | |  | |  | |  | |  | |  | |  | |  | | | |  | |  | |  | |  | |  | |  | |  | |  | |  | |  | |  | |  | |  | |  | |  | |  | |  | |  | |  | |  | |  | |  | |  | |  | |  | |  |  |  |
| EGFR | chr7:55174794-55174794 | P753S | C>T | Single Nucleotide Variants (SNVs) | COSMIC(Lung) | No | |  | |  | |  | |  | |  | |  | |  | | | |  | |  | |  | |  | |  | |  | |  | |  | |  | |  | |  | |  | |  | |  | |  | |  | |  | |  | |  | |  | |  | |  | |  | |  | |  | |  |  |  |
| EGFR | chr7:55174794-55174794 | P753S | CCG>AGC | Multi- nucleotide variants (MNVs) | COSMIC(Lung) | No | |  | |  | |  | |  | |  | |  | |  | | | |  | |  | |  | |  | |  | |  | |  | |  | |  | |  | |  | |  | |  | |  | |  | |  | |  | |  | |  | |  | |  | |  | |  | |  | |  | |  |  |  |
| EGFR | chr7:55174794-55174794 | P753S | CCG>AGT | Multi- nucleotide variants (MNVs) | COSMIC(Lung) | No | |  | |  | |  | |  | |  | |  | |  | | | |  | |  | |  | |  | |  | |  | |  | |  | |  | |  | |  | |  | |  | |  | |  | |  | |  | |  | |  | |  | |  | |  | |  | |  | |  | |  |  |  |
| EGFR | chr7:55181324-55181324 | H773dup | C>CCCA | Insertion | Tissue(Lung) | No | |  | |  | |  | |  | |  | |  | |  | | | |  | |  | |  | |  | |  | |  | |  | |  | |  | |  | |  | |  | |  | |  | |  | |  | |  | |  | |  | |  | |  | |  | |  | |  | |  | |  |  |  |
| EGFR | chr7:55181326-55181326 | H773Y | C>T | Single Nucleotide Variants (SNVs) | COSMIC(Lung) | No | |  | |  | |  | |  | |  | |  | |  | | | |  | |  | |  | |  | |  | |  | |  | |  | |  | |  | |  | |  | |  | |  | |  | |  | |  | |  | |  | |  | |  | |  | |  | |  | |  | |  |  |  |
| EGFR | chr7:55181327-55181327 | H773L | A>T | Single Nucleotide Variants (SNVs) | COSMIC(Lung)-Tissue(Lung) | No | |  | |  | |  | |  | |  | |  | |  | | | |  | |  | |  | |  | |  | |  | |  | |  | |  | |  | |  | |  | |  | |  | |  | |  | |  | |  | |  | |  | |  | |  | |  | |  | |  | |  |  |  |
| EGFR | chr7:55181329-55181329 | V774M | G>A | Single Nucleotide Variants (SNVs) | COSMIC(Lung)-Tissue(Lung) | No | |  | |  | |  | |  | |  | |  | |  | | | |  | |  | |  | |  | |  | |  | |  | |  | |  | |  | |  | |  | |  | |  | |  | |  | |  | |  | |  | |  | |  | |  | |  | |  | |  | |  |  |  |
| EGFR | chr7:55181330-55181330 | V774A | T>C | Single Nucleotide Variants (SNVs) | COSMIC(Lung) | No | |  | |  | |  | |  | |  | |  | |  | | | |  | |  | |  | |  | |  | |  | |  | |  | |  | |  | |  | |  | |  | |  | |  | |  | |  | |  | |  | |  | |  | |  | |  | |  | |  | |  |  |  |
| EGFR | chr7:55181335-55181335 | R776C | C>T | Single Nucleotide Variants (SNVs) | COSMIC(Lung) | No | |  | |  | |  | |  | |  | |  | |  | | | |  | |  | |  | |  | |  | |  | |  | |  | |  | |  | |  | |  | |  | |  | |  | |  | |  | |  | |  | |  | |  | |  | |  | |  | |  | |  |  |  |
| EGFR | chr7:55181335-55181335 | R776G | C>G | Single Nucleotide Variants (SNVs) | COSMIC(Lung) | No | |  | |  | |  | |  | |  | |  | |  | | | |  | |  | |  | |  | |  | |  | |  | |  | |  | |  | |  | |  | |  | |  | |  | |  | |  | |  | |  | |  | |  | |  | |  | |  | |  | |  |  |  |
| EGFR | chr7:55181336-55181336 | R776H | G>A | Single Nucleotide Variants (SNVs) | COSMIC(Lung) | No | |  | |  | |  | |  | |  | |  | |  | | | |  | |  | |  | |  | |  | |  | |  | |  | |  | |  | |  | |  | |  | |  | |  | |  | |  | |  | |  | |  | |  | |  | |  | |  | |  | |  |  |  |
| EGFR | chr7:55181378-55181378 | T790M | C>T | Single Nucleotide Variants (SNVs) | COSMIC(Lung)-Tissue(Lung) | Yes | |  | |  | |  | |  | |  | |  | |  | | | |  | |  | |  | |  | |  | |  | |  | |  | |  | |  | |  | |  | |  | |  | |  | |  | |  | |  | |  | |  | |  | |  | |  | |  | |  | |  |  |  |
| EGFR | chr7:55181383-55181383 | L792* | CTC>TAA | Multi- nucleotide variants (MNVs) | COSMIC(Lung) | No | |  | |  | |  | |  | |  | |  | |  | | | |  | |  | |  | |  | |  | |  | |  | |  | |  | |  | |  | |  | |  | |  | |  | |  | |  | |  | |  | |  | |  | |  | |  | |  | |  | |  |  |  |
| EGFR | chr7:55181383-55181383 | L792* | CTC>TAG | Multi- nucleotide variants (MNVs) | COSMIC(Lung) | No | |  | |  | |  | |  | |  | |  | |  | | | |  | |  | |  | |  | |  | |  | |  | |  | |  | |  | |  | |  | |  | |  | |  | |  | |  | |  | |  | |  | |  | |  | |  | |  | |  | |  |  |  |
| EGFR | chr7:55181383-55181383 | L792* | CTC>TGA | Multi- nucleotide variants (MNVs) | COSMIC(Lung) | No | |  | |  | |  | |  | |  | |  | |  | | | |  | |  | |  | |  | |  | |  | |  | |  | |  | |  | |  | |  | |  | |  | |  | |  | |  | |  | |  | |  | |  | |  | |  | |  | |  | |  |  |  |
| EGFR | chr7:55181383-55181383 | L792F | C>T | Single Nucleotide Variants (SNVs) | COSMIC(Lung) | No | |  | |  | |  | |  | |  | |  | |  | | | |  | |  | |  | |  | |  | |  | |  | |  | |  | |  | |  | |  | |  | |  | |  | |  | |  | |  | |  | |  | |  | |  | |  | |  | |  | |  |  |  |
| EGFR | chr7:55181383-55181383 | L792V | C>G | Single Nucleotide Variants (SNVs) | COSMIC(Lung) | No | |  | |  | |  | |  | |  | |  | |  | | | |  | |  | |  | |  | |  | |  | |  | |  | |  | |  | |  | |  | |  | |  | |  | |  | |  | |  | |  | |  | |  | |  | |  | |  | |  | |  |  |  |
| EGFR | chr7:55181384-55181384 | L792H | T>A | Single Nucleotide Variants (SNVs) | COSMIC(Lung) | No | |  | |  | |  | |  | |  | |  | |  | | | |  | |  | |  | |  | |  | |  | |  | |  | |  | |  | |  | |  | |  | |  | |  | |  | |  | |  | |  | |  | |  | |  | |  | |  | |  | |  |  |  |
| EGFR | chr7:55181384-55181384 | L792R | T>G | Single Nucleotide Variants (SNVs) | COSMIC(Lung) | No | |  | |  | |  | |  | |  | |  | |  | | | |  | |  | |  | |  | |  | |  | |  | |  | |  | |  | |  | |  | |  | |  | |  | |  | |  | |  | |  | |  | |  | |  | |  | |  | |  | |  |  |  |
| EGFR | chr7:55181395-55181395 | G796R | G>C | Single Nucleotide Variants (SNVs) | COSMIC(Lung) | No | |  | |  | |  | |  | |  | |  | |  | | | |  | |  | |  | |  | |  | |  | |  | |  | |  | |  | |  | |  | |  | |  | |  | |  | |  | |  | |  | |  | |  | |  | |  | |  | |  | |  |  |  |
| EGFR | chr7:55181395-55181395 | G796R | GGC>AGA | Multi- nucleotide variants (MNVs) | COSMIC(Lung) | No | |  | |  | |  | |  | |  | |  | |  | | | |  | |  | |  | |  | |  | |  | |  | |  | |  | |  | |  | |  | |  | |  | |  | |  | |  | |  | |  | |  | |  | |  | |  | |  | |  | |  |  |  |
| EGFR | chr7:55181395-55181395 | G796R | GGC>AGG | Multi- nucleotide variants (MNVs) | COSMIC(Lung) | No | |  | |  | |  | |  | |  | |  | |  | | | |  | |  | |  | |  | |  | |  | |  | |  | |  | |  | |  | |  | |  | |  | |  | |  | |  | |  | |  | |  | |  | |  | |  | |  | |  | |  |  |  |
| EGFR | chr7:55181395-55181395 | G796S | G>A | Single Nucleotide Variants (SNVs) | COSMIC(Lung) | No | |  | |  | |  | |  | |  | |  | |  | | | |  | |  | |  | |  | |  | |  | |  | |  | |  | |  | |  | |  | |  | |  | |  | |  | |  | |  | |  | |  | |  | |  | |  | |  | |  | |  |  |  |
| EGFR | chr7:55181395-55181395 | G796S | GG>TC | Multi- nucleotide variants (MNVs) | COSMIC(Lung) | No | |  | |  | |  | |  | |  | |  | |  | | | |  | |  | |  | |  | |  | |  | |  | |  | |  | |  | |  | |  | |  | |  | |  | |  | |  | |  | |  | |  | |  | |  | |  | |  | |  | |  |  |  |
| EGFR | chr7:55181396-55181396 | G796A | G>C | Single Nucleotide Variants (SNVs) | COSMIC(Lung) | No | |  | |  | |  | |  | |  | |  | |  | | | |  | |  | |  | |  | |  | |  | |  | |  | |  | |  | |  | |  | |  | |  | |  | |  | |  | |  | |  | |  | |  | |  | |  | |  | |  | |  |  |  |
| EGFR | chr7:55181396-55181396 | G796D | G>A | Single Nucleotide Variants (SNVs) | COSMIC(Lung) | No | |  | |  | |  | |  | |  | |  | |  | | | |  | |  | |  | |  | |  | |  | |  | |  | |  | |  | |  | |  | |  | |  | |  | |  | |  | |  | |  | |  | |  | |  | |  | |  | |  | |  |  |  |
| EGFR | chr7:55181397-55181397 | C797S | CT>AA | Multi- nucleotide variants (MNVs) | COSMIC(Lung) | No | |  | |  | |  | |  | |  | |  | |  | | | |  | |  | |  | |  | |  | |  | |  | |  | |  | |  | |  | |  | |  | |  | |  | |  | |  | |  | |  | |  | |  | |  | |  | |  | |  | |  |  |  |
| EGFR | chr7:55181398-55181398 | C797D | TG>GA | Multi- nucleotide variants (MNVs) | COSMIC(Lung) | No | |  | |  | |  | |  | |  | |  | |  | | | |  | |  | |  | |  | |  | |  | |  | |  | |  | |  | |  | |  | |  | |  | |  | |  | |  | |  | |  | |  | |  | |  | |  | |  | |  | |  |  |  |
| EGFR | chr7:55181398-55181398 | C797G | T>G | Single Nucleotide Variants (SNVs) | COSMIC(Lung) | No | |  | |  | |  | |  | |  | |  | |  | | | |  | |  | |  | |  | |  | |  | |  | |  | |  | |  | |  | |  | |  | |  | |  | |  | |  | |  | |  | |  | |  | |  | |  | |  | |  | |  |  |  |
| EGFR | chr7:55181398-55181398 | C797S | T>A | Single Nucleotide Variants (SNVs) | COSMIC(Lung) | No | |  | |  | |  | |  | |  | |  | |  | | | |  | |  | |  | |  | |  | |  | |  | |  | |  | |  | |  | |  | |  | |  | |  | |  | |  | |  | |  | |  | |  | |  | |  | |  | |  | |  |  |  |
| EGFR | chr7:55181399-55181399 | C797S | G>C | Single Nucleotide Variants (SNVs) | COSMIC(Lung) | No | |  | |  | |  | |  | |  | |  | |  | | | |  | |  | |  | |  | |  | |  | |  | |  | |  | |  | |  | |  | |  | |  | |  | |  | |  | |  | |  | |  | |  | |  | |  | |  | |  | |  |  |  |
| EGFR | chr7:55181399-55181399 | C797Y | G>A | Single Nucleotide Variants (SNVs) | COSMIC(Lung) | No | |  | |  | |  | |  | |  | |  | |  | | | |  | |  | |  | |  | |  | |  | |  | |  | |  | |  | |  | |  | |  | |  | |  | |  | |  | |  | |  | |  | |  | |  | |  | |  | |  | |  |  |  |
| EGFR | chr7:55181400-55181400 | C797W | C>G | Single Nucleotide Variants (SNVs) | COSMIC(Lung) | No | |  | |  | |  | |  | |  | |  | |  | | | |  | |  | |  | |  | |  | |  | |  | |  | |  | |  | |  | |  | |  | |  | |  | |  | |  | |  | |  | |  | |  | |  | |  | |  | |  | |  |  |  |
| EGFR | chr7:55181401-55181401 | L798I | C>A | Single Nucleotide Variants (SNVs) | COSMIC(Lung) | No | |  | |  | |  | |  | |  | |  | |  | | | |  | |  | |  | |  | |  | |  | |  | |  | |  | |  | |  | |  | |  | |  | |  | |  | |  | |  | |  | |  | |  | |  | |  | |  | |  | |  |  |  |
| EGFR | chr7:55191741-55191741 | R831H | G>A | Single Nucleotide Variants (SNVs) | COSMIC(Lung) | No | |  | |  | |  | |  | |  | |  | |  | | | |  | |  | |  | |  | |  | |  | |  | |  | |  | |  | |  | |  | |  | |  | |  | |  | |  | |  | |  | |  | |  | |  | |  | |  | |  | |  |  |  |
| EGFR | chr7:55191746-55191746 | L833V | T>G | Single Nucleotide Variants (SNVs) | COSMIC(Lung) | No | |  | |  | |  | |  | |  | |  | |  | | | |  | |  | |  | |  | |  | |  | |  | |  | |  | |  | |  | |  | |  | |  | |  | |  | |  | |  | |  | |  | |  | |  | |  | |  | |  | |  |  |  |
| EGFR | chr7:55191749-55191749 | V834L | G>C | Single Nucleotide Variants (SNVs) | COSMIC(Lung) | No | |  | |  | |  | |  | |  | |  | |  | | | |  | |  | |  | |  | |  | |  | |  | |  | |  | |  | |  | |  | |  | |  | |  | |  | |  | |  | |  | |  | |  | |  | |  | |  | |  | |  |  |  |
| EGFR | chr7:55191749-55191749 | V834L | G>T | Single Nucleotide Variants (SNVs) | COSMIC(Lung) | No | |  | |  | |  | |  | |  | |  | |  | | | |  | |  | |  | |  | |  | |  | |  | |  | |  | |  | |  | |  | |  | |  | |  | |  | |  | |  | |  | |  | |  | |  | |  | |  | |  | |  |  |  |
| EGFR | chr7:55191753-55191753 | H835L | A>T | Single Nucleotide Variants (SNVs) | COSMIC(Lung) | No | |  | |  | |  | |  | |  | |  | |  | | | |  | |  | |  | |  | |  | |  | |  | |  | |  | |  | |  | |  | |  | |  | |  | |  | |  | |  | |  | |  | |  | |  | |  | |  | |  | |  |  |  |
| EGFR | chr7:55191755-55191755 | R836C | C>T | Single Nucleotide Variants (SNVs) | COSMIC(Lung) | No | |  | |  | |  | |  | |  | |  | |  | | | |  | |  | |  | |  | |  | |  | |  | |  | |  | |  | |  | |  | |  | |  | |  | |  | |  | |  | |  | |  | |  | |  | |  | |  | |  | |  |  |  |
| EGFR | chr7:55191758-55191758 | D837N | G>A | Single Nucleotide Variants (SNVs) | COSMIC(Lung) | No | |  | |  | |  | |  | |  | |  | |  | | | |  | |  | |  | |  | |  | |  | |  | |  | |  | |  | |  | |  | |  | |  | |  | |  | |  | |  | |  | |  | |  | |  | |  | |  | |  | |  |  |  |
| EGFR | chr7:55191761-55191761 | L838V | C>G | Single Nucleotide Variants (SNVs) | COSMIC(Lung) | No | |  | |  | |  | |  | |  | |  | |  | | | |  | |  | |  | |  | |  | |  | |  | |  | |  | |  | |  | |  | |  | |  | |  | |  | |  | |  | |  | |  | |  | |  | |  | |  | |  | |  |  |  |
| EGFR | chr7:55191762-55191762 | L838P | T>C | Single Nucleotide Variants (SNVs) | COSMIC(Lung) | No | |  | |  | |  | |  | |  | |  | |  | | | |  | |  | |  | |  | |  | |  | |  | |  | |  | |  | |  | |  | |  | |  | |  | |  | |  | |  | |  | |  | |  | |  | |  | |  | |  | |  |  |  |
| EGFR | chr7:55191809-55191809 | T854A | A>G | Single Nucleotide Variants (SNVs) | COSMIC(Lung) | No | |  | |  | |  | |  | |  | |  | |  | | | |  | |  | |  | |  | |  | |  | |  | |  | |  | |  | |  | |  | |  | |  | |  | |  | |  | |  | |  | |  | |  | |  | |  | |  | |  | |  |  |  |
| EGFR | chr7:55191809-55191809 | T854S | A>T | Single Nucleotide Variants (SNVs) | COSMIC(Lung) | No | |  | |  | |  | |  | |  | |  | |  | | | |  | |  | |  | |  | |  | |  | |  | |  | |  | |  | |  | |  | |  | |  | |  | |  | |  | |  | |  | |  | |  | |  | |  | |  | |  | |  |  |  |
| EGFR | chr7:55191810-55191810 | T854I | C>T | Single Nucleotide Variants (SNVs) | COSMIC(Lung) | No | |  | |  | |  | |  | |  | |  | |  | | | |  | |  | |  | |  | |  | |  | |  | |  | |  | |  | |  | |  | |  | |  | |  | |  | |  | |  | |  | |  | |  | |  | |  | |  | |  | |  |  |  |
| EGFR | chr7:55191810-55191810 | T854S | CA>GC | Multi- nucleotide variants (MNVs) | COSMIC(Lung) | No | |  | |  | |  | |  | |  | |  | |  | | | |  | |  | |  | |  | |  | |  | |  | |  | |  | |  | |  | |  | |  | |  | |  | |  | |  | |  | |  | |  | |  | |  | |  | |  | |  | |  |  |  |
| EGFR | chr7:55191810-55191810 | T854S | CA>GT | Multi- nucleotide variants (MNVs) | COSMIC(Lung) | No | |  | |  | |  | |  | |  | |  | |  | | | |  | |  | |  | |  | |  | |  | |  | |  | |  | |  | |  | |  | |  | |  | |  | |  | |  | |  | |  | |  | |  | |  | |  | |  | |  | |  |  |  |
| EGFR | chr7:55191822-55191822 | L858R | T>G | Single Nucleotide Variants (SNVs) | COSMIC(Lung)-Tissue(Lung) | Yes | |  | |  | |  | |  | |  | |  | |  | | | |  | |  | |  | |  | |  | |  | |  | |  | |  | |  | |  | |  | |  | |  | |  | |  | |  | |  | |  | |  | |  | |  | |  | |  | |  | |  |  |  |
| EGFR | chr7:55191824-55191824 | A859T | G>A | Single Nucleotide Variants (SNVs) | COSMIC(Lung) | No | |  | |  | |  | |  | |  | |  | |  | | | |  | |  | |  | |  | |  | |  | |  | |  | |  | |  | |  | |  | |  | |  | |  | |  | |  | |  | |  | |  | |  | |  | |  | |  | |  | |  |  |  |
| EGFR | chr7:55191831-55191831 | L861Q | T>A | Single Nucleotide Variants (SNVs) | COSMIC(Lung) | Yes | |  | |  | |  | |  | |  | |  | |  | | | |  | |  | |  | |  | |  | |  | |  | |  | |  | |  | |  | |  | |  | |  | |  | |  | |  | |  | |  | |  | |  | |  | |  | |  | |  | |  |  |  |
| EGFR | chr7:55191839-55191839 | A864T | G>A | Single Nucleotide Variants (SNVs) | COSMIC(Lung) | No | |  | |  | |  | |  | |  | |  | |  | | | |  | |  | |  | |  | |  | |  | |  | |  | |  | |  | |  | |  | |  | |  | |  | |  | |  | |  | |  | |  | |  | |  | |  | |  | |  | |  |  |  |
| EGFR | chr7:55191840-55191840 | A864V | C>T | Single Nucleotide Variants (SNVs) | COSMIC(Lung) | No | |  | |  | |  | |  | |  | |  | |  | | | |  | |  | |  | |  | |  | |  | |  | |  | |  | |  | |  | |  | |  | |  | |  | |  | |  | |  | |  | |  | |  | |  | |  | |  | |  | |  |  |  |
| EGFR | chr7:55191852-55191852 | E868G | A>G | Single Nucleotide Variants (SNVs) | COSMIC(Lung) | No | |  | |  | |  | |  | |  | |  | |  | | | |  | |  | |  | |  | |  | |  | |  | |  | |  | |  | |  | |  | |  | |  | |  | |  | |  | |  | |  | |  | |  | |  | |  | |  | |  | |  |  |  |
| ERBB2 | chr17:39723966-39723966 | L755A | TT>GC | Multi- nucleotide variants (MNVs) | COSMIC(Lung) | No | |  | |  | |  | |  | |  | |  | |  | | | |  | |  | |  | |  | |  | |  | |  | |  | |  | |  | |  | |  | |  | |  | |  | |  | |  | |  | |  | |  | |  | |  | |  | |  | |  | |  |  |  |
| ERBB2 | chr17:39723966-39723966 | L755M | T>A | Single Nucleotide Variants (SNVs) | COSMIC(Lung,Breast) | No | |  | |  | |  | |  | |  | |  | |  | | | |  | |  | |  | |  | |  | |  | |  | |  | |  | |  | |  | |  | |  | |  | |  | |  | |  | |  | |  | |  | |  | |  | |  | |  | |  | |  |  |  |
| ERBB2 | chr17:39723966-39723966 | L755P | TT>CC | Multi- nucleotide variants (MNVs) | COSMIC(Lung,Breast) | No | |  | |  | |  | |  | |  | |  | |  | | | |  | |  | |  | |  | |  | |  | |  | |  | |  | |  | |  | |  | |  | |  | |  | |  | |  | |  | |  | |  | |  | |  | |  | |  | |  | |  |  |  |
| ERBB2 | chr17:39723966-39723966 | L755R | TT>AG | Multi- nucleotide variants (MNVs) | COSMIC(Lung,Breast) | No | |  | |  | |  | |  | |  | |  | |  | | | |  | |  | |  | |  | |  | |  | |  | |  | |  | |  | |  | |  | |  | |  | |  | |  | |  | |  | |  | |  | |  | |  | |  | |  | |  | |  |  |  |
| ERBB2 | chr17:39723966-39723966 | L755R | TT>CG | Multi- nucleotide variants (MNVs) | COSMIC(Lung,Breast) | No | |  | |  | |  | |  | |  | |  | |  | | | |  | |  | |  | |  | |  | |  | |  | |  | |  | |  | |  | |  | |  | |  | |  | |  | |  | |  | |  | |  | |  | |  | |  | |  | |  | |  |  |  |
| ERBB2 | chr17:39723966-39723966 | L755S | TTG>AGC | Multi- nucleotide variants (MNVs) | COSMIC(Lung,Breast,Gastric,HCC) | Yes | |  | |  | |  | |  | |  | |  | |  | | | |  | |  | |  | |  | |  | |  | |  | |  | |  | |  | |  | |  | |  | |  | |  | |  | |  | |  | |  | |  | |  | |  | |  | |  | |  | |  |  |  |
| ERBB2 | chr17:39723966-39723966 | L755S | TTG>AGT | Multi- nucleotide variants (MNVs) | COSMIC(Lung,Breast,Gastric,HCC) | Yes | |  | |  | |  | |  | |  | |  | |  | | | |  | |  | |  | |  | |  | |  | |  | |  | |  | |  | |  | |  | |  | |  | |  | |  | |  | |  | |  | |  | |  | |  | |  | |  | |  | |  |  |  |
| ERBB2 | chr17:39723967-39723967 | L755S | T>C | Single Nucleotide Variants (SNVs) | COSMIC(Lung,CRC,Breast,Gastric,HCC) | Yes | |  | |  | |  | |  | |  | |  | |  | | | |  | |  | |  | |  | |  | |  | |  | |  | |  | |  | |  | |  | |  | |  | |  | |  | |  | |  | |  | |  | |  | |  | |  | |  | |  | |  |  |  |
| ERBB2 | chr17:39723967-39723967 | L755W | T>G | Single Nucleotide Variants (SNVs) | COSMIC(Lung,Breast) | No | |  | |  | |  | |  | |  | |  | |  | | | |  | |  | |  | |  | |  | |  | |  | |  | |  | |  | |  | |  | |  | |  | |  | |  | |  | |  | |  | |  | |  | |  | |  | |  | |  | |  |  |  |
| ERBB2 | chr17:39723968-39723968 | L755F | G>C | Single Nucleotide Variants (SNVs) | COSMIC(Lung,Breast) | No | |  | |  | |  | |  | |  | |  | |  | | | |  | |  | |  | |  | |  | |  | |  | |  | |  | |  | |  | |  | |  | |  | |  | |  | |  | |  | |  | |  | |  | |  | |  | |  | |  | |  |  |  |
| ERBB2 | chr17:39723968-39723968 | L755F | G>T | Single Nucleotide Variants (SNVs) | COSMIC(Lung,Breast) | No | |  | |  | |  | |  | |  | |  | |  | | | |  | |  | |  | |  | |  | |  | |  | |  | |  | |  | |  | |  | |  | |  | |  | |  | |  | |  | |  | |  | |  | |  | |  | |  | |  | |  |  |  |
| ERBB2 | chr17:39724004-39724004 | I767M | C>G | Single Nucleotide Variants (SNVs) | COSMIC(Lung)-Tissue(Gastric) | No | |  | |  | |  | |  | |  | |  | |  | | | |  | |  | |  | |  | |  | |  | |  | |  | |  | |  | |  | |  | |  | |  | |  | |  | |  | |  | |  | |  | |  | |  | |  | |  | |  | |  |  |  |
| ERBB2 | chr17:39724008-39724008 | D769H | G>C | Single Nucleotide Variants (SNVs) | COSMIC(Lung) | No | |  | |  | |  | |  | |  | |  | |  | | | |  | |  | |  | |  | |  | |  | |  | |  | |  | |  | |  | |  | |  | |  | |  | |  | |  | |  | |  | |  | |  | |  | |  | |  | |  | |  |  |  |
| ERBB2 | chr17:39724008-39724008 | D769N | G>A | Single Nucleotide Variants (SNVs) | COSMIC(Lung) | No | |  | |  | |  | |  | |  | |  | |  | | | |  | |  | |  | |  | |  | |  | |  | |  | |  | |  | |  | |  | |  | |  | |  | |  | |  | |  | |  | |  | |  | |  | |  | |  | |  | |  |  |  |
| ERBB2 | chr17:39724008-39724008 | D769Y | G>T | Single Nucleotide Variants (SNVs) | COSMIC(Lung,HCC) | No | |  | |  | |  | |  | |  | |  | |  | | | |  | |  | |  | |  | |  | |  | |  | |  | |  | |  | |  | |  | |  | |  | |  | |  | |  | |  | |  | |  | |  | |  | |  | |  | |  | |  |  |  |
| ERBB2 | chr17:39724742-39724742 | Y772_A775dup | C>CATACGTGATGGC | Insertion | COSMIC(Lung) | No | |  | |  | |  | |  | |  | |  | |  | | | |  | |  | |  | |  | |  | |  | |  | |  | |  | |  | |  | |  | |  | |  | |  | |  | |  | |  | |  | |  | |  | |  | |  | |  | |  | |  |  |  |
| ERBB2 | chr17:39724743-39724743 | Y772_A775dup | T>TTACGTGATGGCT | Insertion | COSMIC(Lung) | No | |  | |  | |  | |  | |  | |  | |  | | | |  | |  | |  | |  | |  | |  | |  | |  | |  | |  | |  | |  | |  | |  | |  | |  | |  | |  | |  | |  | |  | |  | |  | |  | |  | |  |  |  |
| ERBB2 | chr17:39724744-39724744 | G776C | G>T | Single Nucleotide Variants (SNVs) | COSMIC(Lung) | No | |  | |  | |  | |  | |  | |  | |  | | | |  | |  | |  | |  | |  | |  | |  | |  | |  | |  | |  | |  | |  | |  | |  | |  | |  | |  | |  | |  | |  | |  | |  | |  | |  | |  |  |  |
| ERBB2 | chr17:39724744-39724744 | G776DelInsLC | G>GCTTT | Insertion | COSMIC(Lung) | No | |  | |  | |  | |  | |  | |  | |  | | | |  | |  | |  | |  | |  | |  | |  | |  | |  | |  | |  | |  | |  | |  | |  | |  | |  | |  | |  | |  | |  | |  | |  | |  | |  | |  |  |  |
| ERBB2 | chr17:39724744-39724744 | G776DelInsLC | G>GTTAT | Insertion | COSMIC(Lung) | No | |  | |  | |  | |  | |  | |  | |  | | | |  | |  | |  | |  | |  | |  | |  | |  | |  | |  | |  | |  | |  | |  | |  | |  | |  | |  | |  | |  | |  | |  | |  | |  | |  | |  |  |  |
| ERBB2 | chr17:39724744-39724744 | G776DelInsLC | G>GTTGT | Insertion | COSMIC(Lung) | No | |  | |  | |  | |  | |  | |  | |  | | | |  | |  | |  | |  | |  | |  | |  | |  | |  | |  | |  | |  | |  | |  | |  | |  | |  | |  | |  | |  | |  | |  | |  | |  | |  | |  |  |  |
| ERBB2 | chr17:39724744-39724744 | G776DelInsVC | G>GTAT | Insertion | COSMIC(Lung) | No | |  | |  | |  | |  | |  | |  | |  | | | |  | |  | |  | |  | |  | |  | |  | |  | |  | |  | |  | |  | |  | |  | |  | |  | |  | |  | |  | |  | |  | |  | |  | |  | |  | |  |  |  |
| ERBB2 | chr17:39724744-39724744 | G776DelInsVC | G>GTCT | Insertion | COSMIC(Lung) | No | |  | |  | |  | |  | |  | |  | |  | | | |  | |  | |  | |  | |  | |  | |  | |  | |  | |  | |  | |  | |  | |  | |  | |  | |  | |  | |  | |  | |  | |  | |  | |  | |  | |  |  |  |
| ERBB2 | chr17:39724744-39724744 | G776DelInsVC | G>GTGT | Insertion | COSMIC(Lung) | No | |  | |  | |  | |  | |  | |  | |  | | | |  | |  | |  | |  | |  | |  | |  | |  | |  | |  | |  | |  | |  | |  | |  | |  | |  | |  | |  | |  | |  | |  | |  | |  | |  | |  |  |  |
| ERBB2 | chr17:39724744-39724744 | G776DelInsVC | G>GTTT | Insertion | COSMIC(Lung) | No | |  | |  | |  | |  | |  | |  | |  | | | |  | |  | |  | |  | |  | |  | |  | |  | |  | |  | |  | |  | |  | |  | |  | |  | |  | |  | |  | |  | |  | |  | |  | |  | |  | |  |  |  |
| ERBB2 | chr17:39724744-39724744 | G776S | G>A | Single Nucleotide Variants (SNVs) | COSMIC(Lung) | No | |  | |  | |  | |  | |  | |  | |  | | | |  | |  | |  | |  | |  | |  | |  | |  | |  | |  | |  | |  | |  | |  | |  | |  | |  | |  | |  | |  | |  | |  | |  | |  | |  | |  |  |  |
| ERBB2 | chr17:39724744-39724744 | G776S | GG>TC | Multi- nucleotide variants (MNVs) | COSMIC(Lung) | No | |  | |  | |  | |  | |  | |  | |  | | | |  | |  | |  | |  | |  | |  | |  | |  | |  | |  | |  | |  | |  | |  | |  | |  | |  | |  | |  | |  | |  | |  | |  | |  | |  | |  |  |  |
| ERBB2 | chr17:39724745-39724745 | G776V | G>T | Single Nucleotide Variants (SNVs) | COSMIC(Lung) | No | |  | |  | |  | |  | |  | |  | |  | | | |  | |  | |  | |  | |  | |  | |  | |  | |  | |  | |  | |  | |  | |  | |  | |  | |  | |  | |  | |  | |  | |  | |  | |  | |  | |  |  |  |
| ERBB2 | chr17:39724747-39724747 | V777L | G>C | Single Nucleotide Variants (SNVs) | COSMIC(Lung) | No | |  | |  | |  | |  | |  | |  | |  | | | |  | |  | |  | |  | |  | |  | |  | |  | |  | |  | |  | |  | |  | |  | |  | |  | |  | |  | |  | |  | |  | |  | |  | |  | |  | |  |  |  |
| ERBB2 | chr17:39724747-39724747 | V777L | G>T | Single Nucleotide Variants (SNVs) | COSMIC(Lung) | No | |  | |  | |  | |  | |  | |  | |  | | | |  | |  | |  | |  | |  | |  | |  | |  | |  | |  | |  | |  | |  | |  | |  | |  | |  | |  | |  | |  | |  | |  | |  | |  | |  | |  |  |  |
| ERBB2 | chr17:39724747-39724747 | V777M | G>A | Single Nucleotide Variants (SNVs) | COSMIC(Lung,Gastric,HCC) | No | |  | |  | |  | |  | |  | |  | |  | | | |  | |  | |  | |  | |  | |  | |  | |  | |  | |  | |  | |  | |  | |  | |  | |  | |  | |  | |  | |  | |  | |  | |  | |  | |  | |  |  |  |
| ERBB2 | chr17:39724750-39724750 | G778dup | G>GGGA | Insertion | COSMIC(Lung) | No | |  | |  | |  | |  | |  | |  | |  | | | |  | |  | |  | |  | |  | |  | |  | |  | |  | |  | |  | |  | |  | |  | |  | |  | |  | |  | |  | |  | |  | |  | |  | |  | |  | |  |  |  |
| ERBB2 | chr17:39724750-39724750 | G778dup | G>GGGC | Insertion | COSMIC(Lung) | No | |  | |  | |  | |  | |  | |  | |  | | | |  | |  | |  | |  | |  | |  | |  | |  | |  | |  | |  | |  | |  | |  | |  | |  | |  | |  | |  | |  | |  | |  | |  | |  | |  | |  |  |  |
| ERBB2 | chr17:39724752-39724752 | G778dup | C>CGGG | Insertion | COSMIC(Lung) | No | |  | |  | |  | |  | |  | |  | |  | | | |  | |  | |  | |  | |  | |  | |  | |  | |  | |  | |  | |  | |  | |  | |  | |  | |  | |  | |  | |  | |  | |  | |  | |  | |  | |  |  |  |
| ERBB2 | chr17:39724752-39724752 | G778dup | C>CTGG | Insertion | COSMIC(Lung) | No | |  | |  | |  | |  | |  | |  | |  | | | |  | |  | |  | |  | |  | |  | |  | |  | |  | |  | |  | |  | |  | |  | |  | |  | |  | |  | |  | |  | |  | |  | |  | |  | |  | |  |  |  |
| ERBB2 | chr17:39724757-39724757 | G778_P780dup | C>CCGGCTCCCC | Insertion | COSMIC(Lung) | No | |  | |  | |  | |  | |  | |  | |  | | | |  | |  | |  | |  | |  | |  | |  | |  | |  | |  | |  | |  | |  | |  | |  | |  | |  | |  | |  | |  | |  | |  | |  | |  | |  | |  |  |  |
| ERBB2 | chr17:39724757-39724757 | G778_P780dup | C>CGGGCTCCCC | Insertion | COSMIC(Lung) | No | |  | |  | |  | |  | |  | |  | |  | | | |  | |  | |  | |  | |  | |  | |  | |  | |  | |  | |  | |  | |  | |  | |  | |  | |  | |  | |  | |  | |  | |  | |  | |  | |  | |  |  |  |
| ERBB2 | chr17:39724757-39724757 | G778_P780dup | C>CTGGCTCCCC | Insertion | COSMIC(Lung) | No | |  | |  | |  | |  | |  | |  | |  | | | |  | |  | |  | |  | |  | |  | |  | |  | |  | |  | |  | |  | |  | |  | |  | |  | |  | |  | |  | |  | |  | |  | |  | |  | |  | |  |  |  |
| ERBB2 | chr17:39724758-39724758 | G778_P780dup | A>AGGCTCCCCA | Insertion | COSMIC(Lung) | No | |  | |  | |  | |  | |  | |  | |  | | | |  | |  | |  | |  | |  | |  | |  | |  | |  | |  | |  | |  | |  | |  | |  | |  | |  | |  | |  | |  | |  | |  | |  | |  | |  | |  |  |  |
| FBXW7 | chr4:152324294-152324294 | S582L | G>A | Single Nucleotide Variants (SNVs) | Tissue(CRC)-COSMIC(CRC) | No | |  | |  | |  | |  | |  | |  | |  | | | |  | |  | |  | |  | |  | |  | |  | |  | |  | |  | |  | |  | |  | |  | |  | |  | |  | |  | |  | |  | |  | |  | |  | |  | |  | |  |  |  |
| FBXW7 | chr4:152326136-152326136 | R505H | C>T | Single Nucleotide Variants (SNVs) | COSMIC(CRC) | No | |  | |  | |  | |  | |  | |  | |  | | | |  | |  | |  | |  | |  | |  | |  | |  | |  | |  | |  | |  | |  | |  | |  | |  | |  | |  | |  | |  | |  | |  | |  | |  | |  | |  |  |  |
| FBXW7 | chr4:152326137-152326137 | R505C | G>A | Single Nucleotide Variants (SNVs) | Tissue(CRC)-COSMIC(CRC) | No | |  | |  | |  | |  | |  | |  | |  | | | |  | |  | |  | |  | |  | |  | |  | |  | |  | |  | |  | |  | |  | |  | |  | |  | |  | |  | |  | |  | |  | |  | |  | |  | |  | |  |  |  |
| FBXW7 | chr4:152326214-152326214 | R479Q | C>T | Single Nucleotide Variants (SNVs) | Tissue(CRC) | No | |  | |  | |  | |  | |  | |  | |  | | | |  | |  | |  | |  | |  | |  | |  | |  | |  | |  | |  | |  | |  | |  | |  | |  | |  | |  | |  | |  | |  | |  | |  | |  | |  | |  |  |  |
| FBXW7 | chr4:152326215-152326215 | R479* | G>A | Single Nucleotide Variants (SNVs) | Tissue(CRC)-COSMIC(CRC) | No | |  | |  | |  | |  | |  | |  | |  | | | |  | |  | |  | |  | |  | |  | |  | |  | |  | |  | |  | |  | |  | |  | |  | |  | |  | |  | |  | |  | |  | |  | |  | |  | |  | |  |  |  |
| GATA3 | chr10:8069541-8069541 | N332Efs*21 | G>GG | Insertion | COSMIC(Breast) | No | |  | |  | |  | |  | |  | |  | |  | | | |  | |  | |  | |  | |  | |  | |  | |  | |  | |  | |  | |  | |  | |  | |  | |  | |  | |  | |  | |  | |  | |  | |  | |  | |  | |  |  |  |
| GATA3 | chr10:8069550-8069550 | D336Gfs*17 | T>TG | Insertion | Tissue(Breast) | No | |  | |  | |  | |  | |  | |  | |  | | | |  | |  | |  | |  | |  | |  | |  | |  | |  | |  | |  | |  | |  | |  | |  | |  | |  | |  | |  | |  | |  | |  | |  | |  | |  | |  |  |  |
| GATA3 | chr10:8069554-8069554 | D336Gfs*17 | G>GG | Insertion | COSMIC(Breast) | No | |  | |  | |  | |  | |  | |  | |  | | | |  | |  | |  | |  | |  | |  | |  | |  | |  | |  | |  | |  | |  | |  | |  | |  | |  | |  | |  | |  | |  | |  | |  | |  | |  | |  |  |  |
| GATA3 | chr10:8069554-8069554 | D336Gfs*21 | G>GGG | Insertion | COSMIC(Breast) | No | |  | |  | |  | |  | |  | |  | |  | | | |  | |  | |  | |  | |  | |  | |  | |  | |  | |  | |  | |  | |  | |  | |  | |  | |  | |  | |  | |  | |  | |  | |  | |  | |  | |  |  |  |
| GATA3 | chr10:8073890-8073890 | S402Vfs*106 | T>TG | Insertion | Tissue(Breast) | No | |  | |  | |  | |  | |  | |  | |  | | | |  | |  | |  | |  | |  | |  | |  | |  | |  | |  | |  | |  | |  | |  | |  | |  | |  | |  | |  | |  | |  | |  | |  | |  | |  | |  |  |  |
| GATA3 | chr10:8073895-8073895 | L404Pfs*104 | T>TC | Insertion | Tissue(Breast) | No | |  | |  | |  | |  | |  | |  | |  | | | |  | |  | |  | |  | |  | |  | |  | |  | |  | |  | |  | |  | |  | |  | |  | |  | |  | |  | |  | |  | |  | |  | |  | |  | |  | |  |  |  |
| GATA3 | chr10:8073909-8073909 | S408Afs*99 | CTC>C | Deletion | COSMIC(Breast) | No | |  | |  | |  | |  | |  | |  | |  | | | |  | |  | |  | |  | |  | |  | |  | |  | |  | |  | |  | |  | |  | |  | |  | |  | |  | |  | |  | |  | |  | |  | |  | |  | |  | |  |  |  |
| GATA3 | chr10:8073911-8073911 | P409Afs*99 | C>CA | Insertion | COSMIC(Breast) | No | |  | |  | |  | |  | |  | |  | |  | | | |  | |  | |  | |  | |  | |  | |  | |  | |  | |  | |  | |  | |  | |  | |  | |  | |  | |  | |  | |  | |  | |  | |  | |  | |  | |  |  |  |
| GATA3 | chr10:8073911-8073911 | P409Afs*99 | C>CC | Insertion | COSMIC(Breast) | No | |  | |  | |  | |  | |  | |  | |  | | | |  | |  | |  | |  | |  | |  | |  | |  | |  | |  | |  | |  | |  | |  | |  | |  | |  | |  | |  | |  | |  | |  | |  | |  | |  | |  |  |  |
| GATA3 | chr10:8073911-8073911 | P409Afs*99 | C>CT | Insertion | COSMIC(Breast) | No | |  | |  | |  | |  | |  | |  | |  | | | |  | |  | |  | |  | |  | |  | |  | |  | |  | |  | |  | |  | |  | |  | |  | |  | |  | |  | |  | |  | |  | |  | |  | |  | |  | |  |  |  |
| GATA3 | chr10:8073912-8073912 | P409Afs*99 | G>GG | Insertion | COSMIC(Breast) | No | |  | |  | |  | |  | |  | |  | |  | | | |  | |  | |  | |  | |  | |  | |  | |  | |  | |  | |  | |  | |  | |  | |  | |  | |  | |  | |  | |  | |  | |  | |  | |  | |  | |  |  |  |
| GATA3 | chr10:8073912-8073912 | P409Sfs*99 | G>GT | Insertion | COSMIC(Breast) | No | |  | |  | |  | |  | |  | |  | |  | | | |  | |  | |  | |  | |  | |  | |  | |  | |  | |  | |  | |  | |  | |  | |  | |  | |  | |  | |  | |  | |  | |  | |  | |  | |  | |  |  |  |
| GNAS | chr20:58909365-58909365 | R844C | C>T | Single Nucleotide Variants (SNVs) | COSMIC(CRC,HCC) | No | |  | |  | |  | |  | |  | |  | |  | | | |  | |  | |  | |  | |  | |  | |  | |  | |  | |  | |  | |  | |  | |  | |  | |  | |  | |  | |  | |  | |  | |  | |  | |  | |  | |  |  |  |
| GNAS | chr20:58909366-58909366 | R844H | G>A | Single Nucleotide Variants (SNVs) | Tissue(HCC)-COSMIC(CRC) | No | |  | |  | |  | |  | |  | |  | |  | | | |  | |  | |  | |  | |  | |  | |  | |  | |  | |  | |  | |  | |  | |  | |  | |  | |  | |  | |  | |  | |  | |  | |  | |  | |  | |  |  |  |
| HRAS | chr11:533873-533873 | Q61H | C>A | Single Nucleotide Variants (SNVs) | COSMIC(CRC) | No | |  | |  | |  | |  | |  | |  | |  | | | |  | |  | |  | |  | |  | |  | |  | |  | |  | |  | |  | |  | |  | |  | |  | |  | |  | |  | |  | |  | |  | |  | |  | |  | |  | |  |  |  |
| HRAS | chr11:533873-533873 | Q61H | C>G | Single Nucleotide Variants (SNVs) | COSMIC(CRC) | No | |  | |  | |  | |  | |  | |  | |  | | | |  | |  | |  | |  | |  | |  | |  | |  | |  | |  | |  | |  | |  | |  | |  | |  | |  | |  | |  | |  | |  | |  | |  | |  | |  | |  |  |  |
| HRAS | chr11:533874-533874 | Q61L | T>A | Single Nucleotide Variants (SNVs) | COSMIC(CRC) | No | |  | |  | |  | |  | |  | |  | |  | | | |  | |  | |  | |  | |  | |  | |  | |  | |  | |  | |  | |  | |  | |  | |  | |  | |  | |  | |  | |  | |  | |  | |  | |  | |  | |  |  |  |
| HRAS | chr11:533874-533874 | Q61R | T>C | Single Nucleotide Variants (SNVs) | COSMIC(CRC) | No | |  | |  | |  | |  | |  | |  | |  | | | |  | |  | |  | |  | |  | |  | |  | |  | |  | |  | |  | |  | |  | |  | |  | |  | |  | |  | |  | |  | |  | |  | |  | |  | |  | |  |  |  |
| HRAS | chr11:533875-533875 | Q61K | G>T | Single Nucleotide Variants (SNVs) | COSMIC(CRC) | No | |  | |  | |  | |  | |  | |  | |  | | | |  | |  | |  | |  | |  | |  | |  | |  | |  | |  | |  | |  | |  | |  | |  | |  | |  | |  | |  | |  | |  | |  | |  | |  | |  | |  |  |  |
| HRAS | chr11:533880-533880 | A59G | G>C | Single Nucleotide Variants (SNVs) | COSMIC(CRC) | No | |  | |  | |  | |  | |  | |  | |  | | | |  | |  | |  | |  | |  | |  | |  | |  | |  | |  | |  | |  | |  | |  | |  | |  | |  | |  | |  | |  | |  | |  | |  | |  | |  | |  |  |  |
| HRAS | chr11:533881-533881 | A59T | C>T | Single Nucleotide Variants (SNVs) | COSMIC(CRC) | No | |  | |  | |  | |  | |  | |  | |  | | | |  | |  | |  | |  | |  | |  | |  | |  | |  | |  | |  | |  | |  | |  | |  | |  | |  | |  | |  | |  | |  | |  | |  | |  | |  | |  |  |  |
| HRAS | chr11:534283-534283 | G13dup | C>CCAC | Insertion | COSMIC(CRC) | No | |  | |  | |  | |  | |  | |  | |  | | | |  | |  | |  | |  | |  | |  | |  | |  | |  | |  | |  | |  | |  | |  | |  | |  | |  | |  | |  | |  | |  | |  | |  | |  | |  | |  |  |  |
| HRAS | chr11:534283-534283 | G13dup | C>CCCC | Insertion | COSMIC(CRC) | No | |  | |  | |  | |  | |  | |  | |  | | | |  | |  | |  | |  | |  | |  | |  | |  | |  | |  | |  | |  | |  | |  | |  | |  | |  | |  | |  | |  | |  | |  | |  | |  | |  | |  |  |  |
| HRAS | chr11:534283-534283 | G13dup | C>CCGC | Insertion | COSMIC(CRC) | No | |  | |  | |  | |  | |  | |  | |  | | | |  | |  | |  | |  | |  | |  | |  | |  | |  | |  | |  | |  | |  | |  | |  | |  | |  | |  | |  | |  | |  | |  | |  | |  | |  | |  |  |  |
| HRAS | chr11:534283-534283 | G13dup | C>CCTC | Insertion | COSMIC(CRC) | No | |  | |  | |  | |  | |  | |  | |  | | | |  | |  | |  | |  | |  | |  | |  | |  | |  | |  | |  | |  | |  | |  | |  | |  | |  | |  | |  | |  | |  | |  | |  | |  | |  | |  |  |  |
| HRAS | chr11:534284-534284 | G13R | ACC>CCT | Multi- nucleotide variants (MNVs) | COSMIC(CRC) | No | |  | |  | |  | |  | |  | |  | |  | | | |  | |  | |  | |  | |  | |  | |  | |  | |  | |  | |  | |  | |  | |  | |  | |  | |  | |  | |  | |  | |  | |  | |  | |  | |  | |  |  |  |
| HRAS | chr11:534284-534284 | G13R | ACC>TCT | Multi- nucleotide variants (MNVs) | COSMIC(CRC) | No | |  | |  | |  | |  | |  | |  | |  | | | |  | |  | |  | |  | |  | |  | |  | |  | |  | |  | |  | |  | |  | |  | |  | |  | |  | |  | |  | |  | |  | |  | |  | |  | |  | |  |  |  |
| HRAS | chr11:534285-534285 | G13D | C>T | Single Nucleotide Variants (SNVs) | COSMIC(CRC) | No | |  | |  | |  | |  | |  | |  | |  | | | |  | |  | |  | |  | |  | |  | |  | |  | |  | |  | |  | |  | |  | |  | |  | |  | |  | |  | |  | |  | |  | |  | |  | |  | |  | |  |  |  |
| HRAS | chr11:534285-534285 | G13V | C>A | Single Nucleotide Variants (SNVs) | COSMIC(CRC) | No | |  | |  | |  | |  | |  | |  | |  | | | |  | |  | |  | |  | |  | |  | |  | |  | |  | |  | |  | |  | |  | |  | |  | |  | |  | |  | |  | |  | |  | |  | |  | |  | |  | |  |  |  |
| HRAS | chr11:534286-534286 | G13C | C>A | Single Nucleotide Variants (SNVs) | COSMIC(CRC) | No | |  | |  | |  | |  | |  | |  | |  | | | |  | |  | |  | |  | |  | |  | |  | |  | |  | |  | |  | |  | |  | |  | |  | |  | |  | |  | |  | |  | |  | |  | |  | |  | |  | |  |  |  |
| HRAS | chr11:534286-534286 | G13R | C>G | Single Nucleotide Variants (SNVs) | COSMIC(CRC) | No | |  | |  | |  | |  | |  | |  | |  | | | |  | |  | |  | |  | |  | |  | |  | |  | |  | |  | |  | |  | |  | |  | |  | |  | |  | |  | |  | |  | |  | |  | |  | |  | |  | |  |  |  |
| HRAS | chr11:534287-534287 | G12R | GCC>CCT | Multi- nucleotide variants (MNVs) | COSMIC(CRC) | No | |  | |  | |  | |  | |  | |  | |  | | | |  | |  | |  | |  | |  | |  | |  | |  | |  | |  | |  | |  | |  | |  | |  | |  | |  | |  | |  | |  | |  | |  | |  | |  | |  | |  |  |  |
| HRAS | chr11:534287-534287 | G12R | GCC>TCT | Multi- nucleotide variants (MNVs) | COSMIC(CRC) | No | |  | |  | |  | |  | |  | |  | |  | | | |  | |  | |  | |  | |  | |  | |  | |  | |  | |  | |  | |  | |  | |  | |  | |  | |  | |  | |  | |  | |  | |  | |  | |  | |  | |  |  |  |
| HRAS | chr11:534288-534288 | G12A | C>G | Single Nucleotide Variants (SNVs) | COSMIC(CRC) | No | |  | |  | |  | |  | |  | |  | |  | | | |  | |  | |  | |  | |  | |  | |  | |  | |  | |  | |  | |  | |  | |  | |  | |  | |  | |  | |  | |  | |  | |  | |  | |  | |  | |  |  |  |
| HRAS | chr11:534288-534288 | G12D | C>T | Single Nucleotide Variants (SNVs) | COSMIC(CRC) | No | |  | |  | |  | |  | |  | |  | |  | | | |  | |  | |  | |  | |  | |  | |  | |  | |  | |  | |  | |  | |  | |  | |  | |  | |  | |  | |  | |  | |  | |  | |  | |  | |  | |  |  |  |
| HRAS | chr11:534288-534288 | G12S | CC>GA | Multi- nucleotide variants (MNVs) | COSMIC(CRC) | No | |  | |  | |  | |  | |  | |  | |  | | | |  | |  | |  | |  | |  | |  | |  | |  | |  | |  | |  | |  | |  | |  | |  | |  | |  | |  | |  | |  | |  | |  | |  | |  | |  | |  |  |  |
| HRAS | chr11:534288-534288 | G12V | C>A | Single Nucleotide Variants (SNVs) | COSMIC(CRC) | No | |  | |  | |  | |  | |  | |  | |  | | | |  | |  | |  | |  | |  | |  | |  | |  | |  | |  | |  | |  | |  | |  | |  | |  | |  | |  | |  | |  | |  | |  | |  | |  | |  | |  |  |  |
| HRAS | chr11:534289-534289 | G12C | C>A | Single Nucleotide Variants (SNVs) | COSMIC(CRC) | No | |  | |  | |  | |  | |  | |  | |  | | | |  | |  | |  | |  | |  | |  | |  | |  | |  | |  | |  | |  | |  | |  | |  | |  | |  | |  | |  | |  | |  | |  | |  | |  | |  | |  |  |  |
| HRAS | chr11:534289-534289 | G12R | C>G | Single Nucleotide Variants (SNVs) | COSMIC(CRC) | No | |  | |  | |  | |  | |  | |  | |  | | | |  | |  | |  | |  | |  | |  | |  | |  | |  | |  | |  | |  | |  | |  | |  | |  | |  | |  | |  | |  | |  | |  | |  | |  | |  | |  |  |  |
| HRAS | chr11:534289-534289 | G12S | C>T | Single Nucleotide Variants (SNVs) | COSMIC(CRC) | No | |  | |  | |  | |  | |  | |  | |  | | | |  | |  | |  | |  | |  | |  | |  | |  | |  | |  | |  | |  | |  | |  | |  | |  | |  | |  | |  | |  | |  | |  | |  | |  | |  | |  |  |  |
| KEAP1 | chr19:10489740-10489740 | G480V | C>A | Single Nucleotide Variants (SNVs) | COSMIC(Lung) | No | |  | |  | |  | |  | |  | |  | |  | | | |  | |  | |  | |  | |  | |  | |  | |  | |  | |  | |  | |  | |  | |  | |  | |  | |  | |  | |  | |  | |  | |  | |  | |  | |  | |  |  |  |
| KEAP1 | chr19:10489741-10489741 | G480W | C>A | Single Nucleotide Variants (SNVs) | COSMIC(Lung) | No | |  | |  | |  | |  | |  | |  | |  | | | |  | |  | |  | |  | |  | |  | |  | |  | |  | |  | |  | |  | |  | |  | |  | |  | |  | |  | |  | |  | |  | |  | |  | |  | |  | |  |  |  |
| KEAP1 | chr19:10489770-10489770 | R470H | C>T | Single Nucleotide Variants (SNVs) | COSMIC(Lung) | No | |  | |  | |  | |  | |  | |  | |  | | | |  | |  | |  | |  | |  | |  | |  | |  | |  | |  | |  | |  | |  | |  | |  | |  | |  | |  | |  | |  | |  | |  | |  | |  | |  | |  |  |  |
| KEAP1 | chr19:10489771-10489771 | R470C | G>A | Single Nucleotide Variants (SNVs) | COSMIC(Lung) | No | |  | |  | |  | |  | |  | |  | |  | | | |  | |  | |  | |  | |  | |  | |  | |  | |  | |  | |  | |  | |  | |  | |  | |  | |  | |  | |  | |  | |  | |  | |  | |  | |  | |  |  |  |
| KRAS | chr12:25225626-25225626 | A146S | TGC>ACT | Multi- nucleotide variants (MNVs) | COSMIC(Lung,CRC) | No | |  | |  | |  | |  | |  | |  | |  | | | |  | |  | |  | |  | |  | |  | |  | |  | |  | |  | |  | |  | |  | |  | |  | |  | |  | |  | |  | |  | |  | |  | |  | |  | |  | |  |  |  |
| KRAS | chr12:25225626-25225626 | A146S | TGC>GCT | Multi- nucleotide variants (MNVs) | COSMIC(Lung,CRC) | No | |  | |  | |  | |  | |  | |  | |  | | | |  | |  | |  | |  | |  | |  | |  | |  | |  | |  | |  | |  | |  | |  | |  | |  | |  | |  | |  | |  | |  | |  | |  | |  | |  | |  |  |  |
| KRAS | chr12:25225627-25225627 | A146V | G>A | Single Nucleotide Variants (SNVs) | COSMIC(Lung,CRC)-Tissue(Lung) | No | |  | |  | |  | |  | |  | |  | |  | | | |  | |  | |  | |  | |  | |  | |  | |  | |  | |  | |  | |  | |  | |  | |  | |  | |  | |  | |  | |  | |  | |  | |  | |  | |  | |  |  |  |
| KRAS | chr12:25225628-25225628 | A146P | C>G | Single Nucleotide Variants (SNVs) | COSMIC(Lung,CRC) | No | |  | |  | |  | |  | |  | |  | |  | | | |  | |  | |  | |  | |  | |  | |  | |  | |  | |  | |  | |  | |  | |  | |  | |  | |  | |  | |  | |  | |  | |  | |  | |  | |  | |  |  |  |
| KRAS | chr12:25225628-25225628 | A146S | C>A | Single Nucleotide Variants (SNVs) | COSMIC(Lung,CRC) | No | |  | |  | |  | |  | |  | |  | |  | | | |  | |  | |  | |  | |  | |  | |  | |  | |  | |  | |  | |  | |  | |  | |  | |  | |  | |  | |  | |  | |  | |  | |  | |  | |  | |  |  |  |
| KRAS | chr12:25225628-25225628 | A146T | C>T | Single Nucleotide Variants (SNVs) | COSMIC(Lung,CRC)-Tissue(CRC) | No | |  | |  | |  | |  | |  | |  | |  | | | |  | |  | |  | |  | |  | |  | |  | |  | |  | |  | |  | |  | |  | |  | |  | |  | |  | |  | |  | |  | |  | |  | |  | |  | |  | |  |  |  |
| KRAS | chr12:25225709-25225709 | D119H | C>G | Single Nucleotide Variants (SNVs) | COSMIC(Lung,CRC) | No | |  | |  | |  | |  | |  | |  | |  | | | |  | |  | |  | |  | |  | |  | |  | |  | |  | |  | |  | |  | |  | |  | |  | |  | |  | |  | |  | |  | |  | |  | |  | |  | |  | |  |  |  |
| KRAS | chr12:25225709-25225709 | D119N | C>T | Single Nucleotide Variants (SNVs) | COSMIC(Lung,CRC) | No | |  | |  | |  | |  | |  | |  | |  | | | |  | |  | |  | |  | |  | |  | |  | |  | |  | |  | |  | |  | |  | |  | |  | |  | |  | |  | |  | |  | |  | |  | |  | |  | |  | |  |  |  |
| KRAS | chr12:25225713-25225713 | K117F | TTT>AAA | Multi- nucleotide variants (MNVs) | COSMIC(Lung,CRC) | No | |  | |  | |  | |  | |  | |  | |  | | | |  | |  | |  | |  | |  | |  | |  | |  | |  | |  | |  | |  | |  | |  | |  | |  | |  | |  | |  | |  | |  | |  | |  | |  | |  | |  |  |  |
| KRAS | chr12:25225713-25225713 | K117F | TTT>GAA | Multi- nucleotide variants (MNVs) | COSMIC(Lung,CRC) | No | |  | |  | |  | |  | |  | |  | |  | | | |  | |  | |  | |  | |  | |  | |  | |  | |  | |  | |  | |  | |  | |  | |  | |  | |  | |  | |  | |  | |  | |  | |  | |  | |  | |  |  |  |
| KRAS | chr12:25225713-25225713 | K117N | T>A | Single Nucleotide Variants (SNVs) | COSMIC(Lung,CRC) | No | |  | |  | |  | |  | |  | |  | |  | | | |  | |  | |  | |  | |  | |  | |  | |  | |  | |  | |  | |  | |  | |  | |  | |  | |  | |  | |  | |  | |  | |  | |  | |  | |  | |  |  |  |
| KRAS | chr12:25225713-25225713 | K117N | T>G | Single Nucleotide Variants (SNVs) | COSMIC(Lung,CRC) | No | |  | |  | |  | |  | |  | |  | |  | | | |  | |  | |  | |  | |  | |  | |  | |  | |  | |  | |  | |  | |  | |  | |  | |  | |  | |  | |  | |  | |  | |  | |  | |  | |  | |  |  |  |
| KRAS | chr12:25225714-25225714 | K117R | T>C | Single Nucleotide Variants (SNVs) | COSMIC(Lung,CRC) | No | |  | |  | |  | |  | |  | |  | |  | | | |  | |  | |  | |  | |  | |  | |  | |  | |  | |  | |  | |  | |  | |  | |  | |  | |  | |  | |  | |  | |  | |  | |  | |  | |  | |  |  |  |
| KRAS | chr12:25227341-25227341 | Q61H | T>A | Single Nucleotide Variants (SNVs) | COSMIC(Lung,CRC) | Yes | |  | |  | |  | |  | |  | |  | |  | | | |  | |  | |  | |  | |  | |  | |  | |  | |  | |  | |  | |  | |  | |  | |  | |  | |  | |  | |  | |  | |  | |  | |  | |  | |  | |  |  |  |
| KRAS | chr12:25227341-25227341 | Q61H | T>G | Single Nucleotide Variants (SNVs) | COSMIC(Lung,CRC)-Tissue(CRC) | Yes | |  | |  | |  | |  | |  | |  | |  | | | |  | |  | |  | |  | |  | |  | |  | |  | |  | |  | |  | |  | |  | |  | |  | |  | |  | |  | |  | |  | |  | |  | |  | |  | |  | |  |  |  |
| KRAS | chr12:25227342-25227342 | Q61L | T>A | Single Nucleotide Variants (SNVs) | COSMIC(Lung,CRC) | Yes | |  | |  | |  | |  | |  | |  | |  | | | |  | |  | |  | |  | |  | |  | |  | |  | |  | |  | |  | |  | |  | |  | |  | |  | |  | |  | |  | |  | |  | |  | |  | |  | |  | |  |  |  |
| KRAS | chr12:25227342-25227342 | Q61P | T>G | Single Nucleotide Variants (SNVs) | COSMIC(Lung,CRC) | Yes | |  | |  | |  | |  | |  | |  | |  | | | |  | |  | |  | |  | |  | |  | |  | |  | |  | |  | |  | |  | |  | |  | |  | |  | |  | |  | |  | |  | |  | |  | |  | |  | |  | |  |  |  |
| KRAS | chr12:25227342-25227342 | Q61R | T>C | Single Nucleotide Variants (SNVs) | COSMIC(Lung,CRC) | Yes | |  | |  | |  | |  | |  | |  | |  | | | |  | |  | |  | |  | |  | |  | |  | |  | |  | |  | |  | |  | |  | |  | |  | |  | |  | |  | |  | |  | |  | |  | |  | |  | |  | |  |  |  |
| KRAS | chr12:25227343-25227343 | Q61E | G>C | Single Nucleotide Variants (SNVs) | COSMIC(Lung,CRC) | Yes | |  | |  | |  | |  | |  | |  | |  | | | |  | |  | |  | |  | |  | |  | |  | |  | |  | |  | |  | |  | |  | |  | |  | |  | |  | |  | |  | |  | |  | |  | |  | |  | |  | |  |  |  |
| KRAS | chr12:25227343-25227343 | Q61K | G>T | Single Nucleotide Variants (SNVs) | COSMIC(Lung,CRC) | Yes | |  | |  | |  | |  | |  | |  | |  | | | |  | |  | |  | |  | |  | |  | |  | |  | |  | |  | |  | |  | |  | |  | |  | |  | |  | |  | |  | |  | |  | |  | |  | |  | |  | |  |  |  |
| KRAS | chr12:25227343-25227343 | Q61K | GA>TT | Multi- nucleotide variants (MNVs) | Tissue(CRC) | Yes | |  | |  | |  | |  | |  | |  | |  | | | |  | |  | |  | |  | |  | |  | |  | |  | |  | |  | |  | |  | |  | |  | |  | |  | |  | |  | |  | |  | |  | |  | |  | |  | |  | |  |  |  |
| KRAS | chr12:25227344-25227344 | G60R | ACC>CCT | Multi- nucleotide variants (MNVs) | COSMIC(Lung,CRC) | No | |  | |  | |  | |  | |  | |  | |  | | | |  | |  | |  | |  | |  | |  | |  | |  | |  | |  | |  | |  | |  | |  | |  | |  | |  | |  | |  | |  | |  | |  | |  | |  | |  | |  |  |  |
| KRAS | chr12:25227344-25227344 | G60R | ACC>TCT | Multi- nucleotide variants (MNVs) | COSMIC(Lung,CRC) | No | |  | |  | |  | |  | |  | |  | |  | | | |  | |  | |  | |  | |  | |  | |  | |  | |  | |  | |  | |  | |  | |  | |  | |  | |  | |  | |  | |  | |  | |  | |  | |  | |  | |  |  |  |
| KRAS | chr12:25227345-25227345 | G60D | C>T | Single Nucleotide Variants (SNVs) | COSMIC(Lung,CRC) | No | |  | |  | |  | |  | |  | |  | |  | | | |  | |  | |  | |  | |  | |  | |  | |  | |  | |  | |  | |  | |  | |  | |  | |  | |  | |  | |  | |  | |  | |  | |  | |  | |  | |  |  |  |
| KRAS | chr12:25227346-25227346 | G60R | C>G | Single Nucleotide Variants (SNVs) | COSMIC(Lung,CRC) | No | |  | |  | |  | |  | |  | |  | |  | | | |  | |  | |  | |  | |  | |  | |  | |  | |  | |  | |  | |  | |  | |  | |  | |  | |  | |  | |  | |  | |  | |  | |  | |  | |  | |  |  |  |
| KRAS | chr12:25227348-25227348 | A59E | G>T | Single Nucleotide Variants (SNVs) | COSMIC(Lung,CRC) | No | |  | |  | |  | |  | |  | |  | |  | | | |  | |  | |  | |  | |  | |  | |  | |  | |  | |  | |  | |  | |  | |  | |  | |  | |  | |  | |  | |  | |  | |  | |  | |  | |  | |  |  |  |
| KRAS | chr12:25227348-25227348 | A59G | G>C | Single Nucleotide Variants (SNVs) | COSMIC(Lung)-Tissue(HCC) | No | |  | |  | |  | |  | |  | |  | |  | | | |  | |  | |  | |  | |  | |  | |  | |  | |  | |  | |  | |  | |  | |  | |  | |  | |  | |  | |  | |  | |  | |  | |  | |  | |  | |  |  |  |
| KRAS | chr12:25227349-25227349 | A59T | C>T | Single Nucleotide Variants (SNVs) | COSMIC(Lung,Gastric) | No | |  | |  | |  | |  | |  | |  | |  | | | |  | |  | |  | |  | |  | |  | |  | |  | |  | |  | |  | |  | |  | |  | |  | |  | |  | |  | |  | |  | |  | |  | |  | |  | |  | |  |  |  |
| KRAS | chr12:25227351-25227351 | T58I | G>A | Single Nucleotide Variants (SNVs) | COSMIC(CRC) | No | |  | |  | |  | |  | |  | |  | |  | | | |  | |  | |  | |  | |  | |  | |  | |  | |  | |  | |  | |  | |  | |  | |  | |  | |  | |  | |  | |  | |  | |  | |  | |  | |  | |  |  |  |
| KRAS | chr12:25245314-25245314 | I24N | A>T | Single Nucleotide Variants (SNVs) | COSMIC(CRC) | No | |  | |  | |  | |  | |  | |  | |  | | | |  | |  | |  | |  | |  | |  | |  | |  | |  | |  | |  | |  | |  | |  | |  | |  | |  | |  | |  | |  | |  | |  | |  | |  | |  | |  |  |  |
| KRAS | chr12:25245320-25245320 | Q22L | T>A | Single Nucleotide Variants (SNVs) | COSMIC(CRC) | No | |  | |  | |  | |  | |  | |  | |  | | | |  | |  | |  | |  | |  | |  | |  | |  | |  | |  | |  | |  | |  | |  | |  | |  | |  | |  | |  | |  | |  | |  | |  | |  | |  | |  |  |  |
| KRAS | chr12:25245320-25245320 | Q22R | T>C | Single Nucleotide Variants (SNVs) | COSMIC(CRC) | No | |  | |  | |  | |  | |  | |  | |  | | | |  | |  | |  | |  | |  | |  | |  | |  | |  | |  | |  | |  | |  | |  | |  | |  | |  | |  | |  | |  | |  | |  | |  | |  | |  | |  |  |  |
| KRAS | chr12:25245321-25245321 | Q22E | G>C | Single Nucleotide Variants (SNVs) | COSMIC(CRC) | No | |  | |  | |  | |  | |  | |  | |  | | | |  | |  | |  | |  | |  | |  | |  | |  | |  | |  | |  | |  | |  | |  | |  | |  | |  | |  | |  | |  | |  | |  | |  | |  | |  | |  |  |  |
| KRAS | chr12:25245321-25245321 | Q22K | G>T | Single Nucleotide Variants (SNVs) | COSMIC(CRC) | No | |  | |  | |  | |  | |  | |  | |  | | | |  | |  | |  | |  | |  | |  | |  | |  | |  | |  | |  | |  | |  | |  | |  | |  | |  | |  | |  | |  | |  | |  | |  | |  | |  | |  |  |  |
| KRAS | chr12:25245328-25245328 | L19F | C>A | Single Nucleotide Variants (SNVs) | COSMIC(CRC) | No | |  | |  | |  | |  | |  | |  | |  | | | |  | |  | |  | |  | |  | |  | |  | |  | |  | |  | |  | |  | |  | |  | |  | |  | |  | |  | |  | |  | |  | |  | |  | |  | |  | |  |  |  |
| KRAS | chr12:25245328-25245328 | L19F | C>G | Single Nucleotide Variants (SNVs) | COSMIC(CRC) | No | |  | |  | |  | |  | |  | |  | |  | | | |  | |  | |  | |  | |  | |  | |  | |  | |  | |  | |  | |  | |  | |  | |  | |  | |  | |  | |  | |  | |  | |  | |  | |  | |  | |  |  |  |
| KRAS | chr12:25245332-25245332 | A18D | G>T | Single Nucleotide Variants (SNVs) | COSMIC(Lung,CRC) | No | |  | |  | |  | |  | |  | |  | |  | | | |  | |  | |  | |  | |  | |  | |  | |  | |  | |  | |  | |  | |  | |  | |  | |  | |  | |  | |  | |  | |  | |  | |  | |  | |  | |  |  |  |
| KRAS | chr12:25245345-25245345 | G13dup | C>CCAC | Insertion | COSMIC(CRC) | No | |  | |  | |  | |  | |  | |  | |  | | | |  | |  | |  | |  | |  | |  | |  | |  | |  | |  | |  | |  | |  | |  | |  | |  | |  | |  | |  | |  | |  | |  | |  | |  | |  | |  |  |  |
| KRAS | chr12:25245345-25245345 | G13dup | C>CCCC | Insertion | COSMIC(CRC) | No | |  | |  | |  | |  | |  | |  | |  | | | |  | |  | |  | |  | |  | |  | |  | |  | |  | |  | |  | |  | |  | |  | |  | |  | |  | |  | |  | |  | |  | |  | |  | |  | |  | |  |  |  |
| KRAS | chr12:25245345-25245345 | G13dup | C>CCGC | Insertion | COSMIC(CRC) | No | |  | |  | |  | |  | |  | |  | |  | | | |  | |  | |  | |  | |  | |  | |  | |  | |  | |  | |  | |  | |  | |  | |  | |  | |  | |  | |  | |  | |  | |  | |  | |  | |  | |  |  |  |
| KRAS | chr12:25245345-25245345 | G13dup | C>CCTC | Insertion | COSMIC(CRC) | No | |  | |  | |  | |  | |  | |  | |  | | | |  | |  | |  | |  | |  | |  | |  | |  | |  | |  | |  | |  | |  | |  | |  | |  | |  | |  | |  | |  | |  | |  | |  | |  | |  | |  |  |  |
| KRAS | chr12:25245345-25245345 | V14I | C>T | Single Nucleotide Variants (SNVs) | COSMIC(Lung,CRC) | No | |  | |  | |  | |  | |  | |  | |  | | | |  | |  | |  | |  | |  | |  | |  | |  | |  | |  | |  | |  | |  | |  | |  | |  | |  | |  | |  | |  | |  | |  | |  | |  | |  | |  |  |  |
| KRAS | chr12:25245345-25245345 | V14L | C>A | Single Nucleotide Variants (SNVs) | COSMIC(Lung,CRC) | No | |  | |  | |  | |  | |  | |  | |  | | | |  | |  | |  | |  | |  | |  | |  | |  | |  | |  | |  | |  | |  | |  | |  | |  | |  | |  | |  | |  | |  | |  | |  | |  | |  | |  |  |  |
| KRAS | chr12:25245345-25245345 | V14L | C>G | Single Nucleotide Variants (SNVs) | COSMIC(Lung,CRC) | No | |  | |  | |  | |  | |  | |  | |  | | | |  | |  | |  | |  | |  | |  | |  | |  | |  | |  | |  | |  | |  | |  | |  | |  | |  | |  | |  | |  | |  | |  | |  | |  | |  | |  |  |  |
| KRAS | chr12:25245346-25245346 | G13E | GC>CT | Multi- nucleotide variants (MNVs) | COSMIC(Lung,CRC,Breast) | No | |  | |  | |  | |  | |  | |  | |  | | | |  | |  | |  | |  | |  | |  | |  | |  | |  | |  | |  | |  | |  | |  | |  | |  | |  | |  | |  | |  | |  | |  | |  | |  | |  | |  |  |  |
| KRAS | chr12:25245346-25245346 | G13E | GC>TT | Multi- nucleotide variants (MNVs) | COSMIC(Lung,CRC,Breast) | No | |  | |  | |  | |  | |  | |  | |  | | | |  | |  | |  | |  | |  | |  | |  | |  | |  | |  | |  | |  | |  | |  | |  | |  | |  | |  | |  | |  | |  | |  | |  | |  | |  | |  |  |  |
| KRAS | chr12:25245346-25245346 | G13R | GCC>CCT | Multi- nucleotide variants (MNVs) | COSMIC(Lung,CRC) | Yes | |  | |  | |  | |  | |  | |  | |  | | | |  | |  | |  | |  | |  | |  | |  | |  | |  | |  | |  | |  | |  | |  | |  | |  | |  | |  | |  | |  | |  | |  | |  | |  | |  | |  |  |  |
| KRAS | chr12:25245346-25245346 | G13R | GCC>TCT | Multi- nucleotide variants (MNVs) | COSMIC(Lung,CRC) | Yes | |  | |  | |  | |  | |  | |  | |  | | | |  | |  | |  | |  | |  | |  | |  | |  | |  | |  | |  | |  | |  | |  | |  | |  | |  | |  | |  | |  | |  | |  | |  | |  | |  | |  |  |  |
| KRAS | chr12:25245347-25245347 | G13A | C>G | Single Nucleotide Variants (SNVs) | COSMIC(Lung,CRC,Breast,HCC) | Yes | |  | |  | |  | |  | |  | |  | |  | | | |  | |  | |  | |  | |  | |  | |  | |  | |  | |  | |  | |  | |  | |  | |  | |  | |  | |  | |  | |  | |  | |  | |  | |  | |  | |  |  |  |
| KRAS | chr12:25245347-25245347 | G13D | C>T | Single Nucleotide Variants (SNVs) | COSMIC(Lung,CRC,Breast,HCC,Gastric)-Tissue(CRC,Gastric,HCC) | Yes | |  | |  | |  | |  | |  | |  | |  | | | |  | |  | |  | |  | |  | |  | |  | |  | |  | |  | |  | |  | |  | |  | |  | |  | |  | |  | |  | |  | |  | |  | |  | |  | |  | |  |  |  |
| KRAS | chr12:25245347-25245347 | G13H | CC>TG | Multi- nucleotide variants (MNVs) | COSMIC(Lung,CRC,Breast) | No | |  | |  | |  | |  | |  | |  | |  | | | |  | |  | |  | |  | |  | |  | |  | |  | |  | |  | |  | |  | |  | |  | |  | |  | |  | |  | |  | |  | |  | |  | |  | |  | |  | |  |  |  |
| KRAS | chr12:25245347-25245347 | G13S | CC>GA | Multi- nucleotide variants (MNVs) | COSMIC(Lung,CRC) | Yes | |  | |  | |  | |  | |  | |  | |  | | | |  | |  | |  | |  | |  | |  | |  | |  | |  | |  | |  | |  | |  | |  | |  | |  | |  | |  | |  | |  | |  | |  | |  | |  | |  | |  |  |  |
| KRAS | chr12:25245347-25245347 | G13V | C>A | Single Nucleotide Variants (SNVs) | COSMIC(Lung,CRC,Breast) | Yes | |  | |  | |  | |  | |  | |  | |  | | | |  | |  | |  | |  | |  | |  | |  | |  | |  | |  | |  | |  | |  | |  | |  | |  | |  | |  | |  | |  | |  | |  | |  | |  | |  | |  |  |  |
| KRAS | chr12:25245348-25245348 | G12_G13InsV | C>CTGG | Insertion | COSMIC(CRC) | No | |  | |  | |  | |  | |  | |  | |  | | | |  | |  | |  | |  | |  | |  | |  | |  | |  | |  | |  | |  | |  | |  | |  | |  | |  | |  | |  | |  | |  | |  | |  | |  | |  | |  |  |  |
| KRAS | chr12:25245348-25245348 | G13C | C>A | Single Nucleotide Variants (SNVs) | COSMIC(Lung,CRC,Breast,HCC)-Tissue(Lung) | Yes | |  | |  | |  | |  | |  | |  | |  | | | |  | |  | |  | |  | |  | |  | |  | |  | |  | |  | |  | |  | |  | |  | |  | |  | |  | |  | |  | |  | |  | |  | |  | |  | |  | |  |  |  |
| KRAS | chr12:25245348-25245348 | G13R | C>G | Single Nucleotide Variants (SNVs) | COSMIC(Lung,CRC,Breast) | Yes | |  | |  | |  | |  | |  | |  | |  | | | |  | |  | |  | |  | |  | |  | |  | |  | |  | |  | |  | |  | |  | |  | |  | |  | |  | |  | |  | |  | |  | |  | |  | |  | |  | |  |  |  |
| KRAS | chr12:25245348-25245348 | G13S | C>T | Single Nucleotide Variants (SNVs) | COSMIC(Lung,CRC,Breast,Gastric) | Yes | |  | |  | |  | |  | |  | |  | |  | | | |  | |  | |  | |  | |  | |  | |  | |  | |  | |  | |  | |  | |  | |  | |  | |  | |  | |  | |  | |  | |  | |  | |  | |  | |  | |  |  |  |
| KRAS | chr12:25245349-25245349 | G12E | AC>CT | Multi- nucleotide variants (MNVs) | COSMIC(Lung,CRC,Breast) | Yes | |  | |  | |  | |  | |  | |  | |  | | | |  | |  | |  | |  | |  | |  | |  | |  | |  | |  | |  | |  | |  | |  | |  | |  | |  | |  | |  | |  | |  | |  | |  | |  | |  | |  |  |  |
| KRAS | chr12:25245349-25245349 | G12E | AC>TT | Multi- nucleotide variants (MNVs) | COSMIC(Lung,CRC,Breast) | Yes | |  | |  | |  | |  | |  | |  | |  | | | |  | |  | |  | |  | |  | |  | |  | |  | |  | |  | |  | |  | |  | |  | |  | |  | |  | |  | |  | |  | |  | |  | |  | |  | |  | |  |  |  |
| KRAS | chr12:25245349-25245349 | G12L | ACC>CAA | Multi- nucleotide variants (MNVs) | COSMIC(Lung,CRC) | No | |  | |  | |  | |  | |  | |  | |  | | | |  | |  | |  | |  | |  | |  | |  | |  | |  | |  | |  | |  | |  | |  | |  | |  | |  | |  | |  | |  | |  | |  | |  | |  | |  | |  |  |  |
| KRAS | chr12:25245349-25245349 | G12L | ACC>TAA | Multi- nucleotide variants (MNVs) | COSMIC(Lung,CRC) | No | |  | |  | |  | |  | |  | |  | |  | | | |  | |  | |  | |  | |  | |  | |  | |  | |  | |  | |  | |  | |  | |  | |  | |  | |  | |  | |  | |  | |  | |  | |  | |  | |  | |  |  |  |
| KRAS | chr12:25245349-25245349 | G12R | ACC>CCT | Multi- nucleotide variants (MNVs) | COSMIC(Lung,CRC) | Yes | |  | |  | |  | |  | |  | |  | |  | | | |  | |  | |  | |  | |  | |  | |  | |  | |  | |  | |  | |  | |  | |  | |  | |  | |  | |  | |  | |  | |  | |  | |  | |  | |  | |  |  |  |
| KRAS | chr12:25245349-25245349 | G12R | ACC>TCT | Multi- nucleotide variants (MNVs) | COSMIC(Lung,CRC) | Yes | |  | |  | |  | |  | |  | |  | |  | | | |  | |  | |  | |  | |  | |  | |  | |  | |  | |  | |  | |  | |  | |  | |  | |  | |  | |  | |  | |  | |  | |  | |  | |  | |  | |  |  |  |
| KRAS | chr12:25245349-25245349 | G12W | ACC>CCA | Multi- nucleotide variants (MNVs) | COSMIC(Lung,CRC,Breast) | Yes | |  | |  | |  | |  | |  | |  | |  | | | |  | |  | |  | |  | |  | |  | |  | |  | |  | |  | |  | |  | |  | |  | |  | |  | |  | |  | |  | |  | |  | |  | |  | |  | |  | |  |  |  |
| KRAS | chr12:25245350-25245350 | G12A | C>G | Single Nucleotide Variants (SNVs) | COSMIC(Lung,CRC,Breast,Gastric,HCC)-Tissue(CRC,Gastric) | Yes | |  | |  | |  | |  | |  | |  | |  | | | |  | |  | |  | |  | |  | |  | |  | |  | |  | |  | |  | |  | |  | |  | |  | |  | |  | |  | |  | |  | |  | |  | |  | |  | |  | |  |  |  |
| KRAS | chr12:25245350-25245350 | G12D | C>T | Single Nucleotide Variants (SNVs) | COSMIC(Lung,CRC,Breast,Gastric,HCC)-Tissue(Lung,CRC) | Yes | |  | |  | |  | |  | |  | |  | |  | | | |  | |  | |  | |  | |  | |  | |  | |  | |  | |  | |  | |  | |  | |  | |  | |  | |  | |  | |  | |  | |  | |  | |  | |  | |  | |  |  |  |
| KRAS | chr12:25245350-25245350 | G12F | CC>AA | Multi- nucleotide variants (MNVs) | COSMIC(Lung,CRC,Breast) | Yes | |  | |  | |  | |  | |  | |  | |  | | | |  | |  | |  | |  | |  | |  | |  | |  | |  | |  | |  | |  | |  | |  | |  | |  | |  | |  | |  | |  | |  | |  | |  | |  | |  | |  |  |  |
| KRAS | chr12:25245350-25245350 | G12I | CC>AT | Multi- nucleotide variants (MNVs) | COSMIC(Lung,CRC,Breast) | Yes | |  | |  | |  | |  | |  | |  | |  | | | |  | |  | |  | |  | |  | |  | |  | |  | |  | |  | |  | |  | |  | |  | |  | |  | |  | |  | |  | |  | |  | |  | |  | |  | |  | |  |  |  |
| KRAS | chr12:25245350-25245350 | G12L | CC>AG | Multi- nucleotide variants (MNVs) | COSMIC(Lung,CRC,Breast,Gastric,HCC) | No | |  | |  | |  | |  | |  | |  | |  | | | |  | |  | |  | |  | |  | |  | |  | |  | |  | |  | |  | |  | |  | |  | |  | |  | |  | |  | |  | |  | |  | |  | |  | |  | |  | |  |  |  |
| KRAS | chr12:25245350-25245350 | G12S | CC>GA | Multi- nucleotide variants (MNVs) | COSMIC(Lung,CRC) | Yes | |  | |  | |  | |  | |  | |  | |  | | | |  | |  | |  | |  | |  | |  | |  | |  | |  | |  | |  | |  | |  | |  | |  | |  | |  | |  | |  | |  | |  | |  | |  | |  | |  | |  |  |  |
| KRAS | chr12:25245350-25245350 | G12V | C>A | Single Nucleotide Variants (SNVs) | COSMIC(Lung,CRC,Breast,Gastric,HCC)-Tissue(Lung,CRC) | Yes | |  | |  | |  | |  | |  | |  | |  | | | |  | |  | |  | |  | |  | |  | |  | |  | |  | |  | |  | |  | |  | |  | |  | |  | |  | |  | |  | |  | |  | |  | |  | |  | |  | |  |  |  |
| KRAS | chr12:25245351-25245351 | G12C | C>A | Single Nucleotide Variants (SNVs) | COSMIC(Lung,CRC,Breast,Gastric,HCC)-Tissue(Lung,CRC,HCC) | Yes | |  | |  | |  | |  | |  | |  | |  | | | |  | |  | |  | |  | |  | |  | |  | |  | |  | |  | |  | |  | |  | |  | |  | |  | |  | |  | |  | |  | |  | |  | |  | |  | |  | |  |  |  |
| KRAS | chr12:25245351-25245351 | G12R | C>G | Single Nucleotide Variants (SNVs) | COSMIC(Lung,CRC,Breast,Gastric,HCC)-Tissue(CRC) | Yes | |  | |  | |  | |  | |  | |  | |  | | | |  | |  | |  | |  | |  | |  | |  | |  | |  | |  | |  | |  | |  | |  | |  | |  | |  | |  | |  | |  | |  | |  | |  | |  | |  | |  |  |  |
| KRAS | chr12:25245351-25245351 | G12S | C>T | Single Nucleotide Variants (SNVs) | COSMIC(Lung,CRC,Breast,Gastric,HCC)-Tissue(CRC) | Yes | |  | |  | |  | |  | |  | |  | |  | | | |  | |  | |  | |  | |  | |  | |  | |  | |  | |  | |  | |  | |  | |  | |  | |  | |  | |  | |  | |  | |  | |  | |  | |  | |  | |  |  |  |
| NRAS | chr1:114713895-114713895 | S65R | A>C | Single Nucleotide Variants (SNVs) | COSMIC(CRC) | No | |  | |  | |  | |  | |  | |  | |  | | | |  | |  | |  | |  | |  | |  | |  | |  | |  | |  | |  | |  | |  | |  | |  | |  | |  | |  | |  | |  | |  | |  | |  | |  | |  | |  |  |  |
| NRAS | chr1:114713895-114713895 | S65R | A>T | Single Nucleotide Variants (SNVs) | COSMIC(CRC) | No | |  | |  | |  | |  | |  | |  | |  | | | |  | |  | |  | |  | |  | |  | |  | |  | |  | |  | |  | |  | |  | |  | |  | |  | |  | |  | |  | |  | |  | |  | |  | |  | |  | |  |  |  |
| NRAS | chr1:114713897-114713897 | S65R | T>G | Single Nucleotide Variants (SNVs) | COSMIC(CRC) | No | |  | |  | |  | |  | |  | |  | |  | | | |  | |  | |  | |  | |  | |  | |  | |  | |  | |  | |  | |  | |  | |  | |  | |  | |  | |  | |  | |  | |  | |  | |  | |  | |  | |  |  |  |
| NRAS | chr1:114713907-114713907 | Q61H | T>A | Single Nucleotide Variants (SNVs) | COSMIC(Lung,CRC) | No | |  | |  | |  | |  | |  | |  | |  | | | |  | |  | |  | |  | |  | |  | |  | |  | |  | |  | |  | |  | |  | |  | |  | |  | |  | |  | |  | |  | |  | |  | |  | |  | |  | |  |  |  |
| NRAS | chr1:114713907-114713907 | Q61H | T>G | Single Nucleotide Variants (SNVs) | COSMIC(Lung,CRC) | No | |  | |  | |  | |  | |  | |  | |  | | | |  | |  | |  | |  | |  | |  | |  | |  | |  | |  | |  | |  | |  | |  | |  | |  | |  | |  | |  | |  | |  | |  | |  | |  | |  | |  |  |  |
| NRAS | chr1:114713908-114713908 | Q61L | T>A | Single Nucleotide Variants (SNVs) | COSMIC(Lung,CRC) | No | |  | |  | |  | |  | |  | |  | |  | | | |  | |  | |  | |  | |  | |  | |  | |  | |  | |  | |  | |  | |  | |  | |  | |  | |  | |  | |  | |  | |  | |  | |  | |  | |  | |  |  |  |
| NRAS | chr1:114713908-114713908 | Q61P | T>G | Single Nucleotide Variants (SNVs) | COSMIC(Lung,CRC) | No | |  | |  | |  | |  | |  | |  | |  | | | |  | |  | |  | |  | |  | |  | |  | |  | |  | |  | |  | |  | |  | |  | |  | |  | |  | |  | |  | |  | |  | |  | |  | |  | |  | |  |  |  |
| NRAS | chr1:114713908-114713908 | Q61R | T>C | Single Nucleotide Variants (SNVs) | COSMIC(Lung,CRC)-Tissue(CRC,HCC) | No | |  | |  | |  | |  | |  | |  | |  | | | |  | |  | |  | |  | |  | |  | |  | |  | |  | |  | |  | |  | |  | |  | |  | |  | |  | |  | |  | |  | |  | |  | |  | |  | |  | |  |  |  |
| NRAS | chr1:114713909-114713909 | Q61* | G>A | Single Nucleotide Variants (SNVs) | COSMIC(Lung,CRC) | No | |  | |  | |  | |  | |  | |  | |  | | | |  | |  | |  | |  | |  | |  | |  | |  | |  | |  | |  | |  | |  | |  | |  | |  | |  | |  | |  | |  | |  | |  | |  | |  | |  | |  |  |  |
| NRAS | chr1:114713909-114713909 | Q61E | G>C | Single Nucleotide Variants (SNVs) | COSMIC(Lung,CRC) | No | |  | |  | |  | |  | |  | |  | |  | | | |  | |  | |  | |  | |  | |  | |  | |  | |  | |  | |  | |  | |  | |  | |  | |  | |  | |  | |  | |  | |  | |  | |  | |  | |  | |  |  |  |
| NRAS | chr1:114713909-114713909 | Q61K | G>T | Single Nucleotide Variants (SNVs) | COSMIC(Lung,CRC) | No | |  | |  | |  | |  | |  | |  | |  | | | |  | |  | |  | |  | |  | |  | |  | |  | |  | |  | |  | |  | |  | |  | |  | |  | |  | |  | |  | |  | |  | |  | |  | |  | |  | |  |  |  |
| NRAS | chr1:114713911-114713911 | G60E | C>T | Single Nucleotide Variants (SNVs) | COSMIC(CRC) | No | |  | |  | |  | |  | |  | |  | |  | | | |  | |  | |  | |  | |  | |  | |  | |  | |  | |  | |  | |  | |  | |  | |  | |  | |  | |  | |  | |  | |  | |  | |  | |  | |  | |  |  |  |
| NRAS | chr1:114713914-114713914 | A59G | G>C | Single Nucleotide Variants (SNVs) | COSMIC(CRC) | No | |  | |  | |  | |  | |  | |  | |  | | | |  | |  | |  | |  | |  | |  | |  | |  | |  | |  | |  | |  | |  | |  | |  | |  | |  | |  | |  | |  | |  | |  | |  | |  | |  | |  |  |  |
| NRAS | chr1:114713915-114713915 | A59T | C>T | Single Nucleotide Variants (SNVs) | COSMIC(CRC) | No | |  | |  | |  | |  | |  | |  | |  | | | |  | |  | |  | |  | |  | |  | |  | |  | |  | |  | |  | |  | |  | |  | |  | |  | |  | |  | |  | |  | |  | |  | |  | |  | |  | |  |  |  |
| NRAS | chr1:114713917-114713917 | T58I | G>A | Single Nucleotide Variants (SNVs) | COSMIC(CRC) | No | |  | |  | |  | |  | |  | |  | |  | | | |  | |  | |  | |  | |  | |  | |  | |  | |  | |  | |  | |  | |  | |  | |  | |  | |  | |  | |  | |  | |  | |  | |  | |  | |  | |  |  |  |
| NRAS | chr1:114716109-114716109 | A18T | C>T | Single Nucleotide Variants (SNVs) | COSMIC(Lung,CRC) | No | |  | |  | |  | |  | |  | |  | |  | | | |  | |  | |  | |  | |  | |  | |  | |  | |  | |  | |  | |  | |  | |  | |  | |  | |  | |  | |  | |  | |  | |  | |  | |  | |  | |  |  |  |
| NRAS | chr1:114716122-114716122 | G13R | ACC>CCT | Multi- nucleotide variants (MNVs) | COSMIC(Lung,CRC) | No | |  | |  | |  | |  | |  | |  | |  | | | |  | |  | |  | |  | |  | |  | |  | |  | |  | |  | |  | |  | |  | |  | |  | |  | |  | |  | |  | |  | |  | |  | |  | |  | |  | |  |  |  |
| NRAS | chr1:114716122-114716122 | G13R | ACC>TCT | Multi- nucleotide variants (MNVs) | COSMIC(Lung,CRC) | No | |  | |  | |  | |  | |  | |  | |  | | | |  | |  | |  | |  | |  | |  | |  | |  | |  | |  | |  | |  | |  | |  | |  | |  | |  | |  | |  | |  | |  | |  | |  | |  | |  | |  |  |  |
| NRAS | chr1:114716123-114716123 | G13A | C>G | Single Nucleotide Variants (SNVs) | COSMIC(Lung,CRC) | No | |  | |  | |  | |  | |  | |  | |  | | | |  | |  | |  | |  | |  | |  | |  | |  | |  | |  | |  | |  | |  | |  | |  | |  | |  | |  | |  | |  | |  | |  | |  | |  | |  | |  |  |  |
| NRAS | chr1:114716123-114716123 | G13D | C>T | Single Nucleotide Variants (SNVs) | COSMIC(Lung,CRC) | No | |  | |  | |  | |  | |  | |  | |  | | | |  | |  | |  | |  | |  | |  | |  | |  | |  | |  | |  | |  | |  | |  | |  | |  | |  | |  | |  | |  | |  | |  | |  | |  | |  | |  |  |  |
| NRAS | chr1:114716123-114716123 | G13S | CC>GA | Multi- nucleotide variants (MNVs) | COSMIC(Lung,CRC) | No | |  | |  | |  | |  | |  | |  | |  | | | |  | |  | |  | |  | |  | |  | |  | |  | |  | |  | |  | |  | |  | |  | |  | |  | |  | |  | |  | |  | |  | |  | |  | |  | |  | |  |  |  |
| NRAS | chr1:114716123-114716123 | G13V | C>A | Single Nucleotide Variants (SNVs) | COSMIC(Lung,CRC) | No | |  | |  | |  | |  | |  | |  | |  | | | |  | |  | |  | |  | |  | |  | |  | |  | |  | |  | |  | |  | |  | |  | |  | |  | |  | |  | |  | |  | |  | |  | |  | |  | |  | |  |  |  |
| NRAS | chr1:114716124-114716124 | G13C | C>A | Single Nucleotide Variants (SNVs) | COSMIC(Lung,CRC) | No | |  | |  | |  | |  | |  | |  | |  | | | |  | |  | |  | |  | |  | |  | |  | |  | |  | |  | |  | |  | |  | |  | |  | |  | |  | |  | |  | |  | |  | |  | |  | |  | |  | |  |  |  |
| NRAS | chr1:114716124-114716124 | G13R | C>G | Single Nucleotide Variants (SNVs) | COSMIC(Lung,CRC) | No | |  | |  | |  | |  | |  | |  | |  | | | |  | |  | |  | |  | |  | |  | |  | |  | |  | |  | |  | |  | |  | |  | |  | |  | |  | |  | |  | |  | |  | |  | |  | |  | |  | |  |  |  |
| NRAS | chr1:114716124-114716124 | G13S | C>T | Single Nucleotide Variants (SNVs) | COSMIC(Lung,CRC) | No | |  | |  | |  | |  | |  | |  | |  | | | |  | |  | |  | |  | |  | |  | |  | |  | |  | |  | |  | |  | |  | |  | |  | |  | |  | |  | |  | |  | |  | |  | |  | |  | |  | |  |  |  |
| NRAS | chr1:114716125-114716125 | G12L | ACC>CAA | Multi- nucleotide variants (MNVs) | COSMIC(Lung,CRC) | No | |  | |  | |  | |  | |  | |  | |  | | | |  | |  | |  | |  | |  | |  | |  | |  | |  | |  | |  | |  | |  | |  | |  | |  | |  | |  | |  | |  | |  | |  | |  | |  | |  | |  |  |  |
| NRAS | chr1:114716125-114716125 | G12L | ACC>TAA | Multi- nucleotide variants (MNVs) | COSMIC(Lung,CRC) | No | |  | |  | |  | |  | |  | |  | |  | | | |  | |  | |  | |  | |  | |  | |  | |  | |  | |  | |  | |  | |  | |  | |  | |  | |  | |  | |  | |  | |  | |  | |  | |  | |  | |  |  |  |
| NRAS | chr1:114716125-114716125 | G12R | ACC>CCT | Multi- nucleotide variants (MNVs) | COSMIC(Lung,CRC) | Yes | |  | |  | |  | |  | |  | |  | |  | | | |  | |  | |  | |  | |  | |  | |  | |  | |  | |  | |  | |  | |  | |  | |  | |  | |  | |  | |  | |  | |  | |  | |  | |  | |  | |  |  |  |
| NRAS | chr1:114716125-114716125 | G12R | ACC>TCT | Multi- nucleotide variants (MNVs) | COSMIC(Lung,CRC) | Yes | |  | |  | |  | |  | |  | |  | |  | | | |  | |  | |  | |  | |  | |  | |  | |  | |  | |  | |  | |  | |  | |  | |  | |  | |  | |  | |  | |  | |  | |  | |  | |  | |  | |  |  |  |
| NRAS | chr1:114716126-114716126 | G12A | C>G | Single Nucleotide Variants (SNVs) | COSMIC(Lung,CRC) | Yes | |  | |  | |  | |  | |  | |  | |  | | | |  | |  | |  | |  | |  | |  | |  | |  | |  | |  | |  | |  | |  | |  | |  | |  | |  | |  | |  | |  | |  | |  | |  | |  | |  | |  |  |  |
| NRAS | chr1:114716126-114716126 | G12D | C>T | Single Nucleotide Variants (SNVs) | COSMIC(Lung,CRC)-Tissue(CRC) | Yes | |  | |  | |  | |  | |  | |  | |  | | | |  | |  | |  | |  | |  | |  | |  | |  | |  | |  | |  | |  | |  | |  | |  | |  | |  | |  | |  | |  | |  | |  | |  | |  | |  | |  |  |  |
| NRAS | chr1:114716126-114716126 | G12L | CC>AG | Multi- nucleotide variants (MNVs) | COSMIC(Lung,CRC) | No | |  | |  | |  | |  | |  | |  | |  | | | |  | |  | |  | |  | |  | |  | |  | |  | |  | |  | |  | |  | |  | |  | |  | |  | |  | |  | |  | |  | |  | |  | |  | |  | |  | |  |  |  |
| NRAS | chr1:114716126-114716126 | G12S | CC>GA | Multi- nucleotide variants (MNVs) | COSMIC(Lung,CRC) | Yes | |  | |  | |  | |  | |  | |  | |  | | | |  | |  | |  | |  | |  | |  | |  | |  | |  | |  | |  | |  | |  | |  | |  | |  | |  | |  | |  | |  | |  | |  | |  | |  | |  | |  |  |  |
| NRAS | chr1:114716126-114716126 | G12V | C>A | Single Nucleotide Variants (SNVs) | COSMIC(Lung,CRC) | Yes | |  | |  | |  | |  | |  | |  | |  | | | |  | |  | |  | |  | |  | |  | |  | |  | |  | |  | |  | |  | |  | |  | |  | |  | |  | |  | |  | |  | |  | |  | |  | |  | |  | |  |  |  |
| NRAS | chr1:114716127-114716127 | G12C | C>A | Single Nucleotide Variants (SNVs) | COSMIC(Lung,CRC) | Yes | |  | |  | |  | |  | |  | |  | |  | | | |  | |  | |  | |  | |  | |  | |  | |  | |  | |  | |  | |  | |  | |  | |  | |  | |  | |  | |  | |  | |  | |  | |  | |  | |  | |  |  |  |
| NRAS | chr1:114716127-114716127 | G12R | C>G | Single Nucleotide Variants (SNVs) | COSMIC(Lung,CRC) | Yes | |  | |  | |  | |  | |  | |  | |  | | | |  | |  | |  | |  | |  | |  | |  | |  | |  | |  | |  | |  | |  | |  | |  | |  | |  | |  | |  | |  | |  | |  | |  | |  | |  | |  |  |  |
| NRAS | chr1:114716127-114716127 | G12S | C>T | Single Nucleotide Variants (SNVs) | COSMIC(Lung,CRC) | Yes | |  | |  | |  | |  | |  | |  | |  | | | |  | |  | |  | |  | |  | |  | |  | |  | |  | |  | |  | |  | |  | |  | |  | |  | |  | |  | |  | |  | |  | |  | |  | |  | |  | |  |  |  |
| PIK3CA | chr3:179198937-179198937 | R38C | C>T | Single Nucleotide Variants (SNVs) | COSMIC(Lung,Breast) | No | |  | |  | |  | |  | |  | |  | |  | | | |  | |  | |  | |  | |  | |  | |  | |  | |  | |  | |  | |  | |  | |  | |  | |  | |  | |  | |  | |  | |  | |  | |  | |  | |  | |  |  |  |
| PIK3CA | chr3:179198937-179198937 | R38G | C>G | Single Nucleotide Variants (SNVs) | COSMIC(Lung,Breast) | No | |  | |  | |  | |  | |  | |  | |  | | | |  | |  | |  | |  | |  | |  | |  | |  | |  | |  | |  | |  | |  | |  | |  | |  | |  | |  | |  | |  | |  | |  | |  | |  | |  | |  |  |  |
| PIK3CA | chr3:179198937-179198937 | R38S | C>A | Single Nucleotide Variants (SNVs) | COSMIC(Lung,Breast) | No | |  | |  | |  | |  | |  | |  | |  | | | |  | |  | |  | |  | |  | |  | |  | |  | |  | |  | |  | |  | |  | |  | |  | |  | |  | |  | |  | |  | |  | |  | |  | |  | |  | |  |  |  |
| PIK3CA | chr3:179198937-179198937 | R38S | CG>TC | Multi- nucleotide variants (MNVs) | COSMIC(Lung,Breast) | No | |  | |  | |  | |  | |  | |  | |  | | | |  | |  | |  | |  | |  | |  | |  | |  | |  | |  | |  | |  | |  | |  | |  | |  | |  | |  | |  | |  | |  | |  | |  | |  | |  | |  |  |  |
| PIK3CA | chr3:179198938-179198938 | R38H | G>A | Single Nucleotide Variants (SNVs) | COSMIC(Lung,Breast) | No | |  | |  | |  | |  | |  | |  | |  | | | |  | |  | |  | |  | |  | |  | |  | |  | |  | |  | |  | |  | |  | |  | |  | |  | |  | |  | |  | |  | |  | |  | |  | |  | |  | |  |  |  |
| PIK3CA | chr3:179198938-179198938 | R38L | G>T | Single Nucleotide Variants (SNVs) | COSMIC(Lung,Breast) | No | |  | |  | |  | |  | |  | |  | |  | | | |  | |  | |  | |  | |  | |  | |  | |  | |  | |  | |  | |  | |  | |  | |  | |  | |  | |  | |  | |  | |  | |  | |  | |  | |  | |  |  |  |
| PIK3CA | chr3:179198940-179198940 | E39K | G>A | Single Nucleotide Variants (SNVs) | COSMIC(Lung,Breast) | No | |  | |  | |  | |  | |  | |  | |  | | | |  | |  | |  | |  | |  | |  | |  | |  | |  | |  | |  | |  | |  | |  | |  | |  | |  | |  | |  | |  | |  | |  | |  | |  | |  | |  |  |  |
| PIK3CA | chr3:179199102-179199102 | R93W | C>T | Single Nucleotide Variants (SNVs) | COSMIC(Lung,Breast) | No | |  | |  | |  | |  | |  | |  | |  | | | |  | |  | |  | |  | |  | |  | |  | |  | |  | |  | |  | |  | |  | |  | |  | |  | |  | |  | |  | |  | |  | |  | |  | |  | |  | |  |  |  |
| PIK3CA | chr3:179199103-179199103 | R93Q | G>A | Single Nucleotide Variants (SNVs) | COSMIC(Lung,Breast) | No | |  | |  | |  | |  | |  | |  | |  | | | |  | |  | |  | |  | |  | |  | |  | |  | |  | |  | |  | |  | |  | |  | |  | |  | |  | |  | |  | |  | |  | |  | |  | |  | |  | |  |  |  |
| PIK3CA | chr3:179203760-179203760 | V344M | G>A | Single Nucleotide Variants (SNVs) | COSMIC(Lung,Breast) | No | |  | |  | |  | |  | |  | |  | |  | | | |  | |  | |  | |  | |  | |  | |  | |  | |  | |  | |  | |  | |  | |  | |  | |  | |  | |  | |  | |  | |  | |  | |  | |  | |  | |  |  |  |
| PIK3CA | chr3:179203761-179203761 | V344A | T>C | Single Nucleotide Variants (SNVs) | COSMIC(Lung) | No | |  | |  | |  | |  | |  | |  | |  | | | |  | |  | |  | |  | |  | |  | |  | |  | |  | |  | |  | |  | |  | |  | |  | |  | |  | |  | |  | |  | |  | |  | |  | |  | |  | |  |  |  |
| PIK3CA | chr3:179203761-179203761 | V344G | T>G | Single Nucleotide Variants (SNVs) | COSMIC(Lung)-Tissue(Breast) | No | |  | |  | |  | |  | |  | |  | |  | | | |  | |  | |  | |  | |  | |  | |  | |  | |  | |  | |  | |  | |  | |  | |  | |  | |  | |  | |  | |  | |  | |  | |  | |  | |  | |  |  |  |
| PIK3CA | chr3:179203763-179203763 | N345H | A>C | Single Nucleotide Variants (SNVs) | COSMIC(Lung) | No | |  | |  | |  | |  | |  | |  | |  | | | |  | |  | |  | |  | |  | |  | |  | |  | |  | |  | |  | |  | |  | |  | |  | |  | |  | |  | |  | |  | |  | |  | |  | |  | |  | |  |  |  |
| PIK3CA | chr3:179203763-179203763 | N345S | AA>TC | Multi- nucleotide variants (MNVs) | COSMIC(Lung) | No | |  | |  | |  | |  | |  | |  | |  | | | |  | |  | |  | |  | |  | |  | |  | |  | |  | |  | |  | |  | |  | |  | |  | |  | |  | |  | |  | |  | |  | |  | |  | |  | |  | |  |  |  |
| PIK3CA | chr3:179203764-179203764 | N345I | A>T | Single Nucleotide Variants (SNVs) | COSMIC(Lung,Breast) | No | |  | |  | |  | |  | |  | |  | |  | | | |  | |  | |  | |  | |  | |  | |  | |  | |  | |  | |  | |  | |  | |  | |  | |  | |  | |  | |  | |  | |  | |  | |  | |  | |  | |  |  |  |
| PIK3CA | chr3:179203764-179203764 | N345S | A>G | Single Nucleotide Variants (SNVs) | COSMIC(Lung) | No | |  | |  | |  | |  | |  | |  | |  | | | |  | |  | |  | |  | |  | |  | |  | |  | |  | |  | |  | |  | |  | |  | |  | |  | |  | |  | |  | |  | |  | |  | |  | |  | |  | |  |  |  |
| PIK3CA | chr3:179203764-179203764 | N345T | A>C | Single Nucleotide Variants (SNVs) | COSMIC(Lung) | No | |  | |  | |  | |  | |  | |  | |  | | | |  | |  | |  | |  | |  | |  | |  | |  | |  | |  | |  | |  | |  | |  | |  | |  | |  | |  | |  | |  | |  | |  | |  | |  | |  | |  |  |  |
| PIK3CA | chr3:179203765-179203765 | N345K | T>A | Single Nucleotide Variants (SNVs) | COSMIC(Breast,Gastric,Lung)-Tissue(Breast) | No | |  | |  | |  | |  | |  | |  | |  | | | |  | |  | |  | |  | |  | |  | |  | |  | |  | |  | |  | |  | |  | |  | |  | |  | |  | |  | |  | |  | |  | |  | |  | |  | |  | |  |  |  |
| PIK3CA | chr3:179203765-179203765 | N345K | T>G | Single Nucleotide Variants (SNVs) | COSMIC(Lung) | No | |  | |  | |  | |  | |  | |  | |  | | | |  | |  | |  | |  | |  | |  | |  | |  | |  | |  | |  | |  | |  | |  | |  | |  | |  | |  | |  | |  | |  | |  | |  | |  | |  | |  |  |  |
| PIK3CA | chr3:179210186-179210186 | E418K | G>A | Single Nucleotide Variants (SNVs) | COSMIC(Breast) | No | |  | |  | |  | |  | |  | |  | |  | | | |  | |  | |  | |  | |  | |  | |  | |  | |  | |  | |  | |  | |  | |  | |  | |  | |  | |  | |  | |  | |  | |  | |  | |  | |  | |  |  |  |
| PIK3CA | chr3:179210192-179210192 | C420G | T>G | Single Nucleotide Variants (SNVs) | COSMIC(Lung,Breast) | No | |  | |  | |  | |  | |  | |  | |  | | | |  | |  | |  | |  | |  | |  | |  | |  | |  | |  | |  | |  | |  | |  | |  | |  | |  | |  | |  | |  | |  | |  | |  | |  | |  | |  |  |  |
| PIK3CA | chr3:179210192-179210192 | C420R | T>C | Single Nucleotide Variants (SNVs) | COSMIC(Lung,Breast) | No | |  | |  | |  | |  | |  | |  | |  | | | |  | |  | |  | |  | |  | |  | |  | |  | |  | |  | |  | |  | |  | |  | |  | |  | |  | |  | |  | |  | |  | |  | |  | |  | |  | |  |  |  |
| PIK3CA | chr3:179210192-179210192 | C420R | TGT>AGA | Multi- nucleotide variants (MNVs) | COSMIC(Lung,Breast) | No | |  | |  | |  | |  | |  | |  | |  | | | |  | |  | |  | |  | |  | |  | |  | |  | |  | |  | |  | |  | |  | |  | |  | |  | |  | |  | |  | |  | |  | |  | |  | |  | |  | |  |  |  |
| PIK3CA | chr3:179210192-179210192 | C420R | TGT>AGG | Multi- nucleotide variants (MNVs) | COSMIC(Lung,Breast) | No | |  | |  | |  | |  | |  | |  | |  | | | |  | |  | |  | |  | |  | |  | |  | |  | |  | |  | |  | |  | |  | |  | |  | |  | |  | |  | |  | |  | |  | |  | |  | |  | |  | |  |  |  |
| PIK3CA | chr3:179218294-179218294 | E542K | G>A | Single Nucleotide Variants (SNVs) | COSMIC(Lung,CRC,Breast,Gastric,HCC)-Tissue(CRC) | No | |  | |  | |  | |  | |  | |  | |  | | | |  | |  | |  | |  | |  | |  | |  | |  | |  | |  | |  | |  | |  | |  | |  | |  | |  | |  | |  | |  | |  | |  | |  | |  | |  | |  |  |  |
| PIK3CA | chr3:179218294-179218294 | E542Q | G>C | Single Nucleotide Variants (SNVs) | COSMIC(Lung,CRC,Breast)-Tissue(CRC) | No | |  | |  | |  | |  | |  | |  | |  | | | |  | |  | |  | |  | |  | |  | |  | |  | |  | |  | |  | |  | |  | |  | |  | |  | |  | |  | |  | |  | |  | |  | |  | |  | |  | |  |  |  |
| PIK3CA | chr3:179218295-179218295 | E542A | A>C | Single Nucleotide Variants (SNVs) | COSMIC(Lung,CRC,Breast) | No | |  | |  | |  | |  | |  | |  | |  | | | |  | |  | |  | |  | |  | |  | |  | |  | |  | |  | |  | |  | |  | |  | |  | |  | |  | |  | |  | |  | |  | |  | |  | |  | |  | |  |  |  |
| PIK3CA | chr3:179218295-179218295 | E542G | A>G | Single Nucleotide Variants (SNVs) | COSMIC(Lung,CRC,Breast) | No | |  | |  | |  | |  | |  | |  | |  | | | |  | |  | |  | |  | |  | |  | |  | |  | |  | |  | |  | |  | |  | |  | |  | |  | |  | |  | |  | |  | |  | |  | |  | |  | |  | |  |  |  |
| PIK3CA | chr3:179218295-179218295 | E542V | A>T | Single Nucleotide Variants (SNVs) | COSMIC(Lung,CRC,Breast) | No | |  | |  | |  | |  | |  | |  | |  | | | |  | |  | |  | |  | |  | |  | |  | |  | |  | |  | |  | |  | |  | |  | |  | |  | |  | |  | |  | |  | |  | |  | |  | |  | |  | |  |  |  |
| PIK3CA | chr3:179218303-179218303 | E545K | G>A | Single Nucleotide Variants (SNVs) | COSMIC(Lung,CRC,Breast,Gastric,HCC)-Tissue(CRC,Gastric) | Yes | |  | |  | |  | |  | |  | |  | |  | | | |  | |  | |  | |  | |  | |  | |  | |  | |  | |  | |  | |  | |  | |  | |  | |  | |  | |  | |  | |  | |  | |  | |  | |  | |  | |  |  |  |
| PIK3CA | chr3:179218303-179218303 | E545Q | G>C | Single Nucleotide Variants (SNVs) | COSMIC(Lung,CRC,Breast)-Tissue(Breast) | No | |  | |  | |  | |  | |  | |  | |  | | | |  | |  | |  | |  | |  | |  | |  | |  | |  | |  | |  | |  | |  | |  | |  | |  | |  | |  | |  | |  | |  | |  | |  | |  | |  | |  |  |  |
| PIK3CA | chr3:179218304-179218304 | E545A | A>C | Single Nucleotide Variants (SNVs) | COSMIC(Lung,CRC,Breast) | No | |  | |  | |  | |  | |  | |  | |  | | | |  | |  | |  | |  | |  | |  | |  | |  | |  | |  | |  | |  | |  | |  | |  | |  | |  | |  | |  | |  | |  | |  | |  | |  | |  | |  |  |  |
| PIK3CA | chr3:179218304-179218304 | E545G | A>G | Single Nucleotide Variants (SNVs) | COSMIC(Lung,CRC,Breast)-Tissue(CRC) | No | |  | |  | |  | |  | |  | |  | |  | | | |  | |  | |  | |  | |  | |  | |  | |  | |  | |  | |  | |  | |  | |  | |  | |  | |  | |  | |  | |  | |  | |  | |  | |  | |  | |  |  |  |
| PIK3CA | chr3:179218304-179218304 | E545V | A>T | Single Nucleotide Variants (SNVs) | COSMIC(Lung,CRC,Breast) | No | |  | |  | |  | |  | |  | |  | |  | | | |  | |  | |  | |  | |  | |  | |  | |  | |  | |  | |  | |  | |  | |  | |  | |  | |  | |  | |  | |  | |  | |  | |  | |  | |  | |  |  |  |
| PIK3CA | chr3:179218305-179218305 | E545D | G>C | Single Nucleotide Variants (SNVs) | COSMIC(Lung,CRC,Breast,HCC) | No | |  | |  | |  | |  | |  | |  | |  | | | |  | |  | |  | |  | |  | |  | |  | |  | |  | |  | |  | |  | |  | |  | |  | |  | |  | |  | |  | |  | |  | |  | |  | |  | |  | |  |  |  |
| PIK3CA | chr3:179218305-179218305 | E545D | G>T | Single Nucleotide Variants (SNVs) | COSMIC(Lung,CRC,Breast,HCC) | No | |  | |  | |  | |  | |  | |  | |  | | | |  | |  | |  | |  | |  | |  | |  | |  | |  | |  | |  | |  | |  | |  | |  | |  | |  | |  | |  | |  | |  | |  | |  | |  | |  | |  |  |  |
| PIK3CA | chr3:179218306-179218306 | Q546E | C>G | Single Nucleotide Variants (SNVs) | COSMIC(Lung,CRC,Breast,Gastric) | No | |  | |  | |  | |  | |  | |  | |  | | | |  | |  | |  | |  | |  | |  | |  | |  | |  | |  | |  | |  | |  | |  | |  | |  | |  | |  | |  | |  | |  | |  | |  | |  | |  | |  |  |  |
| PIK3CA | chr3:179218306-179218306 | Q546K | C>A | Single Nucleotide Variants (SNVs) | COSMIC(Lung,CRC,Breast)-Tissue(CRC) | No | |  | |  | |  | |  | |  | |  | |  | | | |  | |  | |  | |  | |  | |  | |  | |  | |  | |  | |  | |  | |  | |  | |  | |  | |  | |  | |  | |  | |  | |  | |  | |  | |  | |  |  |  |
| PIK3CA | chr3:179218307-179218307 | Q546L | A>T | Single Nucleotide Variants (SNVs) | COSMIC(Lung,CRC,Breast) | No | |  | |  | |  | |  | |  | |  | |  | | | |  | |  | |  | |  | |  | |  | |  | |  | |  | |  | |  | |  | |  | |  | |  | |  | |  | |  | |  | |  | |  | |  | |  | |  | |  | |  |  |  |
| PIK3CA | chr3:179218307-179218307 | Q546P | A>C | Single Nucleotide Variants (SNVs) | COSMIC(Lung,CRC,Breast) | No | |  | |  | |  | |  | |  | |  | |  | | | |  | |  | |  | |  | |  | |  | |  | |  | |  | |  | |  | |  | |  | |  | |  | |  | |  | |  | |  | |  | |  | |  | |  | |  | |  | |  |  |  |
| PIK3CA | chr3:179218307-179218307 | Q546R | A>G | Single Nucleotide Variants (SNVs) | COSMIC(Lung,CRC,Breast,Gastric) | No | |  | |  | |  | |  | |  | |  | |  | | | |  | |  | |  | |  | |  | |  | |  | |  | |  | |  | |  | |  | |  | |  | |  | |  | |  | |  | |  | |  | |  | |  | |  | |  | |  | |  |  |  |
| PIK3CA | chr3:179218308-179218308 | Q546H | G>C | Single Nucleotide Variants (SNVs) | COSMIC(Lung,CRC,Breast) | No | |  | |  | |  | |  | |  | |  | |  | | | |  | |  | |  | |  | |  | |  | |  | |  | |  | |  | |  | |  | |  | |  | |  | |  | |  | |  | |  | |  | |  | |  | |  | |  | |  | |  |  |  |
| PIK3CA | chr3:179218308-179218308 | Q546H | G>T | Single Nucleotide Variants (SNVs) | COSMIC(Lung,CRC,Breast) | No | |  | |  | |  | |  | |  | |  | |  | | | |  | |  | |  | |  | |  | |  | |  | |  | |  | |  | |  | |  | |  | |  | |  | |  | |  | |  | |  | |  | |  | |  | |  | |  | |  | |  |  |  |
| PIK3CA | chr3:179218315-179218315 | D549N | G>A | Single Nucleotide Variants (SNVs) | COSMIC(Lung) | No | |  | |  | |  | |  | |  | |  | |  | | | |  | |  | |  | |  | |  | |  | |  | |  | |  | |  | |  | |  | |  | |  | |  | |  | |  | |  | |  | |  | |  | |  | |  | |  | |  | |  |  |  |
| PIK3CA | chr3:179234218-179234218 | Y1021H | T>C | Single Nucleotide Variants (SNVs) | COSMIC(Lung,Breast) | No | |  | |  | |  | |  | |  | |  | |  | | | |  | |  | |  | |  | |  | |  | |  | |  | |  | |  | |  | |  | |  | |  | |  | |  | |  | |  | |  | |  | |  | |  | |  | |  | |  | |  |  |  |
| PIK3CA | chr3:179234219-179234219 | Y1021C | A>G | Single Nucleotide Variants (SNVs) | COSMIC(Lung,Breast) | No | |  | |  | |  | |  | |  | |  | |  | | | |  | |  | |  | |  | |  | |  | |  | |  | |  | |  | |  | |  | |  | |  | |  | |  | |  | |  | |  | |  | |  | |  | |  | |  | |  | |  |  |  |
| PIK3CA | chr3:179234230-179234230 | T1025A | A>G | Single Nucleotide Variants (SNVs) | COSMIC(Lung,Breast) | No | |  | |  | |  | |  | |  | |  | |  | | | |  | |  | |  | |  | |  | |  | |  | |  | |  | |  | |  | |  | |  | |  | |  | |  | |  | |  | |  | |  | |  | |  | |  | |  | |  | |  |  |  |
| PIK3CA | chr3:179234230-179234230 | T1025S | A>T | Single Nucleotide Variants (SNVs) | COSMIC(Lung,Breast) | No | |  | |  | |  | |  | |  | |  | |  | | | |  | |  | |  | |  | |  | |  | |  | |  | |  | |  | |  | |  | |  | |  | |  | |  | |  | |  | |  | |  | |  | |  | |  | |  | |  | |  |  |  |
| PIK3CA | chr3:179234231-179234231 | T1025S | C>G | Single Nucleotide Variants (SNVs) | COSMIC(Lung,Breast) | No | |  | |  | |  | |  | |  | |  | |  | | | |  | |  | |  | |  | |  | |  | |  | |  | |  | |  | |  | |  | |  | |  | |  | |  | |  | |  | |  | |  | |  | |  | |  | |  | |  | |  |  |  |
| PIK3CA | chr3:179234284-179234284 | M1043L | A>C | Single Nucleotide Variants (SNVs) | COSMIC(Lung,Breast) | No | |  | |  | |  | |  | |  | |  | |  | | | |  | |  | |  | |  | |  | |  | |  | |  | |  | |  | |  | |  | |  | |  | |  | |  | |  | |  | |  | |  | |  | |  | |  | |  | |  | |  |  |  |
| PIK3CA | chr3:179234284-179234284 | M1043L | A>T | Single Nucleotide Variants (SNVs) | COSMIC(Lung,Breast) | No | |  | |  | |  | |  | |  | |  | |  | | | |  | |  | |  | |  | |  | |  | |  | |  | |  | |  | |  | |  | |  | |  | |  | |  | |  | |  | |  | |  | |  | |  | |  | |  | |  | |  |  |  |
| PIK3CA | chr3:179234284-179234284 | M1043V | A>G | Single Nucleotide Variants (SNVs) | COSMIC(Lung,Breast) | No | |  | |  | |  | |  | |  | |  | |  | | | |  | |  | |  | |  | |  | |  | |  | |  | |  | |  | |  | |  | |  | |  | |  | |  | |  | |  | |  | |  | |  | |  | |  | |  | |  | |  |  |  |
| PIK3CA | chr3:179234285-179234285 | M1043T | T>C | Single Nucleotide Variants (SNVs) | COSMIC(Lung,Breast) | No | |  | |  | |  | |  | |  | |  | |  | | | |  | |  | |  | |  | |  | |  | |  | |  | |  | |  | |  | |  | |  | |  | |  | |  | |  | |  | |  | |  | |  | |  | |  | |  | |  | |  |  |  |
| PIK3CA | chr3:179234286-179234286 | M1043I | G>A | Single Nucleotide Variants (SNVs) | COSMIC(Lung,Breast) | No | |  | |  | |  | |  | |  | |  | |  | | | |  | |  | |  | |  | |  | |  | |  | |  | |  | |  | |  | |  | |  | |  | |  | |  | |  | |  | |  | |  | |  | |  | |  | |  | |  | |  |  |  |
| PIK3CA | chr3:179234286-179234286 | M1043I | G>C | Single Nucleotide Variants (SNVs) | COSMIC(Lung,Breast) | No | |  | |  | |  | |  | |  | |  | |  | | | |  | |  | |  | |  | |  | |  | |  | |  | |  | |  | |  | |  | |  | |  | |  | |  | |  | |  | |  | |  | |  | |  | |  | |  | |  | |  |  |  |
| PIK3CA | chr3:179234286-179234286 | M1043I | G>T | Single Nucleotide Variants (SNVs) | COSMIC(Lung,Breast) | No | |  | |  | |  | |  | |  | |  | |  | | | |  | |  | |  | |  | |  | |  | |  | |  | |  | |  | |  | |  | |  | |  | |  | |  | |  | |  | |  | |  | |  | |  | |  | |  | |  | |  |  |  |
| PIK3CA | chr3:179234287-179234287 | N1044Y | A>T | Single Nucleotide Variants (SNVs) | COSMIC(Lung,Breast) | No | |  | |  | |  | |  | |  | |  | |  | | | |  | |  | |  | |  | |  | |  | |  | |  | |  | |  | |  | |  | |  | |  | |  | |  | |  | |  | |  | |  | |  | |  | |  | |  | |  | |  |  |  |
| PIK3CA | chr3:179234289-179234289 | N1044K | T>A | Single Nucleotide Variants (SNVs) | COSMIC(Lung,Breast) | No | |  | |  | |  | |  | |  | |  | |  | | | |  | |  | |  | |  | |  | |  | |  | |  | |  | |  | |  | |  | |  | |  | |  | |  | |  | |  | |  | |  | |  | |  | |  | |  | |  | |  |  |  |
| PIK3CA | chr3:179234289-179234289 | N1044K | T>G | Single Nucleotide Variants (SNVs) | COSMIC(Lung,Breast) | No | |  | |  | |  | |  | |  | |  | |  | | | |  | |  | |  | |  | |  | |  | |  | |  | |  | |  | |  | |  | |  | |  | |  | |  | |  | |  | |  | |  | |  | |  | |  | |  | |  | |  |  |  |
| PIK3CA | chr3:179234296-179234296 | H1047Y | C>T | Single Nucleotide Variants (SNVs) | COSMIC(Lung,CRC,Breast) | No | |  | |  | |  | |  | |  | |  | |  | | | |  | |  | |  | |  | |  | |  | |  | |  | |  | |  | |  | |  | |  | |  | |  | |  | |  | |  | |  | |  | |  | |  | |  | |  | |  | |  |  |  |
| PIK3CA | chr3:179234297-179234297 | H1047L | A>T | Single Nucleotide Variants (SNVs) | COSMIC(Lung,CRC,Breast,Gastric) | Yes | |  | |  | |  | |  | |  | |  | |  | | | |  | |  | |  | |  | |  | |  | |  | |  | |  | |  | |  | |  | |  | |  | |  | |  | |  | |  | |  | |  | |  | |  | |  | |  | |  | |  |  |  |
| PIK3CA | chr3:179234297-179234297 | H1047R | A>G | Single Nucleotide Variants (SNVs) | COSMIC(Lung,CRC,Breast,Gastric,HCC)-Tissue(CRC,Breast) | Yes | |  | |  | |  | |  | |  | |  | |  | | | |  | |  | |  | |  | |  | |  | |  | |  | |  | |  | |  | |  | |  | |  | |  | |  | |  | |  | |  | |  | |  | |  | |  | |  | |  | |  |  |  |
| PIK3CA | chr3:179234298-179234298 | H1047Q | T>A | Single Nucleotide Variants (SNVs) | COSMIC(Lung,CRC,Breast) | No | |  | |  | |  | |  | |  | |  | |  | | | |  | |  | |  | |  | |  | |  | |  | |  | |  | |  | |  | |  | |  | |  | |  | |  | |  | |  | |  | |  | |  | |  | |  | |  | |  | |  |  |  |
| PIK3CA | chr3:179234298-179234298 | H1047Q | T>G | Single Nucleotide Variants (SNVs) | COSMIC(Lung,CRC,Breast) | No | |  | |  | |  | |  | |  | |  | |  | | | |  | |  | |  | |  | |  | |  | |  | |  | |  | |  | |  | |  | |  | |  | |  | |  | |  | |  | |  | |  | |  | |  | |  | |  | |  | |  |  |  |
| PIK3CA | chr3:179234302-179234302 | G1049R | G>C | Single Nucleotide Variants (SNVs) | COSMIC(Lung,Breast) | No | |  | |  | |  | |  | |  | |  | |  | | | |  | |  | |  | |  | |  | |  | |  | |  | |  | |  | |  | |  | |  | |  | |  | |  | |  | |  | |  | |  | |  | |  | |  | |  | |  | |  |  |  |
| PIK3CA | chr3:179234302-179234302 | G1049R | GGT>AGA | Multi- nucleotide variants (MNVs) | COSMIC(Lung,Breast) | No | |  | |  | |  | |  | |  | |  | |  | | | |  | |  | |  | |  | |  | |  | |  | |  | |  | |  | |  | |  | |  | |  | |  | |  | |  | |  | |  | |  | |  | |  | |  | |  | |  | |  |  |  |
| PIK3CA | chr3:179234302-179234302 | G1049R | GGT>AGG | Multi- nucleotide variants (MNVs) | COSMIC(Lung,Breast) | No | |  | |  | |  | |  | |  | |  | |  | | | |  | |  | |  | |  | |  | |  | |  | |  | |  | |  | |  | |  | |  | |  | |  | |  | |  | |  | |  | |  | |  | |  | |  | |  | |  | |  |  |  |
| PIK3CA | chr3:179234302-179234302 | G1049S | G>A | Single Nucleotide Variants (SNVs) | COSMIC(Lung,Breast) | No | |  | |  | |  | |  | |  | |  | |  | | | |  | |  | |  | |  | |  | |  | |  | |  | |  | |  | |  | |  | |  | |  | |  | |  | |  | |  | |  | |  | |  | |  | |  | |  | |  | |  |  |  |
| PIK3CA | chr3:179234302-179234302 | G1049S | GG>TC | Multi- nucleotide variants (MNVs) | COSMIC(Lung,Breast) | No | |  | |  | |  | |  | |  | |  | |  | | | |  | |  | |  | |  | |  | |  | |  | |  | |  | |  | |  | |  | |  | |  | |  | |  | |  | |  | |  | |  | |  | |  | |  | |  | |  | |  |  |  |
| PIK3CA | chr3:179234302-179234302 | G1049T | GG>AC | Multi- nucleotide variants (MNVs) | COSMIC(Lung,Breast) | No | |  | |  | |  | |  | |  | |  | |  | | | |  | |  | |  | |  | |  | |  | |  | |  | |  | |  | |  | |  | |  | |  | |  | |  | |  | |  | |  | |  | |  | |  | |  | |  | |  | |  |  |  |
| SMAD4 | chr18:51065548-51065548 | R361C | C>T | Single Nucleotide Variants (SNVs) | Tissue(CRC)-COSMIC(CRC) | No | |  | |  | |  | |  | |  | |  | |  | | | |  | |  | |  | |  | |  | |  | |  | |  | |  | |  | |  | |  | |  | |  | |  | |  | |  | |  | |  | |  | |  | |  | |  | |  | |  | |  |  |  |
| SMAD4 | chr18:51065548-51065548 | R361G | C>G | Single Nucleotide Variants (SNVs) | Tissue(CRC) | No | |  | |  | |  | |  | |  | |  | |  | | | |  | |  | |  | |  | |  | |  | |  | |  | |  | |  | |  | |  | |  | |  | |  | |  | |  | |  | |  | |  | |  | |  | |  | |  | |  | |  |  |  |
| SMAD4 | chr18:51065549-51065549 | R361H | G>A | Single Nucleotide Variants (SNVs) | Tissue(CRC)-COSMIC(CRC) | No | |  | |  | |  | |  | |  | |  | |  | | | |  | |  | |  | |  | |  | |  | |  | |  | |  | |  | |  | |  | |  | |  | |  | |  | |  | |  | |  | |  | |  | |  | |  | |  | |  | |  |  |  |
| STK11 | chr19:1207079-1207079 | G56W | G>T | Single Nucleotide Variants (SNVs) | COSMIC(Lung) | No | |  | |  | |  | |  | |  | |  | |  | | | |  | |  | |  | |  | |  | |  | |  | |  | |  | |  | |  | |  | |  | |  | |  | |  | |  | |  | |  | |  | |  | |  | |  | |  | |  | |  |  |  |
| STK11 | chr19:1207081-1207081 | E57Kfs*7 | GG>G | Deletion | COSMIC(Lung) | No | |  | |  | |  | |  | |  | |  | |  | | | |  | |  | |  | |  | |  | |  | |  | |  | |  | |  | |  | |  | |  | |  | |  | |  | |  | |  | |  | |  | |  | |  | |  | |  | |  | |  |  |  |
| STK11 | chr19:1220630-1220630 | S216F | C>T | Single Nucleotide Variants (SNVs) | COSMIC(Lung) | No | |  | |  | |  | |  | |  | |  | |  | | | |  | |  | |  | |  | |  | |  | |  | |  | |  | |  | |  | |  | |  | |  | |  | |  | |  | |  | |  | |  | |  | |  | |  | |  | |  | |  |  |  |
| STK11 | chr19:1220641-1220641 | Q220* | C>T | Single Nucleotide Variants (SNVs) | COSMIC(Lung) | No | |  | |  | |  | |  | |  | |  | |  | | | |  | |  | |  | |  | |  | |  | |  | |  | |  | |  | |  | |  | |  | |  | |  | |  | |  | |  | |  | |  | |  | |  | |  | |  | |  | |  |  |  |
| STK11 | chr19:1220650-1220650 | E223* | G>T | Single Nucleotide Variants (SNVs) | COSMIC(Lung) | No | |  | |  | |  | |  | |  | |  | |  | | | |  | |  | |  | |  | |  | |  | |  | |  | |  | |  | |  | |  | |  | |  | |  | |  | |  | |  | |  | |  | |  | |  | |  | |  | |  | |  |  |  |
| TCF7L2 | chr10:113165565-113165565 | K468Sfs*23 | AA>A | Deletion | COSMIC(CRC) | No | |  | |  | |  | |  | |  | |  | |  | | | |  | |  | |  | |  | |  | |  | |  | |  | |  | |  | |  | |  | |  | |  | |  | |  | |  | |  | |  | |  | |  | |  | |  | |  | |  | |  |  |  |
| TCF7L2 | chr10:113165566-113165566 | C469Vfs*8 | A>AA | Insertion | COSMIC(CRC) | No | |  | |  | |  | |  | |  | |  | |  | | | |  | |  | |  | |  | |  | |  | |  | |  | |  | |  | |  | |  | |  | |  | |  | |  | |  | |  | |  | |  | |  | |  | |  | |  | |  | |  |  |  |
| TCF7L2 | chr10:113165574-113165574 | R471C | C>T | Single Nucleotide Variants (SNVs) | Tissue(CRC)-COSMIC(CRC) | No | |  | |  | |  | |  | |  | |  | |  | | | |  | |  | |  | |  | |  | |  | |  | |  | |  | |  | |  | |  | |  | |  | |  | |  | |  | |  | |  | |  | |  | |  | |  | |  | |  | |  |  |  |
| TP53 | chr17:7670683-7670683 | R342Qfs*3 | TC>T | Deletion | Tissue(Breast) | No | |  | |  | |  | |  | |  | |  | |  | | | |  | |  | |  | |  | |  | |  | |  | |  | |  | |  | |  | |  | |  | |  | |  | |  | |  | |  | |  | |  | |  | |  | |  | |  | |  | |  |  |  |
| TP53 | chr17:7670685-7670685 | R342* | G>A | Single Nucleotide Variants (SNVs) | Tissue(CRC,Breast,Gastric)-COSMIC(CRC) | No | |  | |  | |  | |  | |  | |  | |  | | | |  | |  | |  | |  | |  | |  | |  | |  | |  | |  | |  | |  | |  | |  | |  | |  | |  | |  | |  | |  | |  | |  | |  | |  | |  | |  |  |  |
| TP53 | chr17:7670685-7670685 | R342Efs*3 | GG>G | Deletion | COSMIC(CRC,Breast) | No | |  | |  | |  | |  | |  | |  | |  | | | |  | |  | |  | |  | |  | |  | |  | |  | |  | |  | |  | |  | |  | |  | |  | |  | |  | |  | |  | |  | |  | |  | |  | |  | |  | |  |  |  |
| TP53 | chr17:7670694-7670694 | E339* | C>A | Single Nucleotide Variants (SNVs) | Tissue(CRC)-COSMIC(CRC,HCC) | No | |  | |  | |  | |  | |  | |  | |  | | | |  | |  | |  | |  | |  | |  | |  | |  | |  | |  | |  | |  | |  | |  | |  | |  | |  | |  | |  | |  | |  | |  | |  | |  | |  | |  |  |  |
| TP53 | chr17:7670699-7670699 | R337L | C>A | Single Nucleotide Variants (SNVs) | Tissue(Gastric) | No | |  | |  | |  | |  | |  | |  | |  | | | |  | |  | |  | |  | |  | |  | |  | |  | |  | |  | |  | |  | |  | |  | |  | |  | |  | |  | |  | |  | |  | |  | |  | |  | |  | |  |  |  |
| TP53 | chr17:7670700-7670700 | R337C | G>A | Single Nucleotide Variants (SNVs) | COSMIC(CRC,Breast,Gastric) | No | |  | |  | |  | |  | |  | |  | |  | | | |  | |  | |  | |  | |  | |  | |  | |  | |  | |  | |  | |  | |  | |  | |  | |  | |  | |  | |  | |  | |  | |  | |  | |  | |  | |  |  |  |
| TP53 | chr17:7670708-7670708 | G334V | C>A | Single Nucleotide Variants (SNVs) | Tissue(HCC) | No | |  | |  | |  | |  | |  | |  | |  | | | |  | |  | |  | |  | |  | |  | |  | |  | |  | |  | |  | |  | |  | |  | |  | |  | |  | |  | |  | |  | |  | |  | |  | |  | |  | |  |  |  |
| TP53 | chr17:7670714-7670714 | I332N | A>T | Single Nucleotide Variants (SNVs) | Tissue(HCC) | No | |  | |  | |  | |  | |  | |  | |  | | | |  | |  | |  | |  | |  | |  | |  | |  | |  | |  | |  | |  | |  | |  | |  | |  | |  | |  | |  | |  | |  | |  | |  | |  | |  | |  |  |  |
| TP53 | chr17:7673609-7673609 | 920-1G>A | C>T | Single Nucleotide Variants (SNVs) | Tissue(Lung) | No | |  | |  | |  | |  | |  | |  | |  | | | |  | |  | |  | |  | |  | |  | |  | |  | |  | |  | |  | |  | |  | |  | |  | |  | |  | |  | |  | |  | |  | |  | |  | |  | |  | |  |  |  |
| TP53 | chr17:7673609-7673609 | 920-1G>C | C>G | Single Nucleotide Variants (SNVs) | Tissue(Lung) | No | |  | |  | |  | |  | |  | |  | |  | | | |  | |  | |  | |  | |  | |  | |  | |  | |  | |  | |  | |  | |  | |  | |  | |  | |  | |  | |  | |  | |  | |  | |  | |  | |  | |  |  |  |
| TP53 | chr17:7673610-7673610 | 920-2A>G | T>C | Single Nucleotide Variants (SNVs) | COSMIC(Lung) | No | |  | |  | |  | |  | |  | |  | |  | | | |  | |  | |  | |  | |  | |  | |  | |  | |  | |  | |  | |  | |  | |  | |  | |  | |  | |  | |  | |  | |  | |  | |  | |  | |  | |  |  |  |
| TP53 | chr17:7673700-7673700 | 919+1G>A | C>T | Single Nucleotide Variants (SNVs) | Tissue(CRC) | No | |  | |  | |  | |  | |  | |  | |  | | | |  | |  | |  | |  | |  | |  | |  | |  | |  | |  | |  | |  | |  | |  | |  | |  | |  | |  | |  | |  | |  | |  | |  | |  | |  | |  |  |  |
| TP53 | chr17:7673704-7673704 | R306* | G>A | Single Nucleotide Variants (SNVs) | Tissue(CRC,Gastric)-COSMIC(CRC,Gastric) | No | |  | |  | |  | |  | |  | |  | |  | | | |  | |  | |  | |  | |  | |  | |  | |  | |  | |  | |  | |  | |  | |  | |  | |  | |  | |  | |  | |  | |  | |  | |  | |  | |  | |  |  |  |
| TP53 | chr17:7673764-7673764 | E286K | C>T | Single Nucleotide Variants (SNVs) | COSMIC(CRC,Breast) | No | |  | |  | |  | |  | |  | |  | |  | | | |  | |  | |  | |  | |  | |  | |  | |  | |  | |  | |  | |  | |  | |  | |  | |  | |  | |  | |  | |  | |  | |  | |  | |  | |  | |  |  |  |
| TP53 | chr17:7673767-7673767 | E285* | C>A | Single Nucleotide Variants (SNVs) | Tissue(CRC) | No | |  | |  | |  | |  | |  | |  | |  | | | |  | |  | |  | |  | |  | |  | |  | |  | |  | |  | |  | |  | |  | |  | |  | |  | |  | |  | |  | |  | |  | |  | |  | |  | |  | |  |  |  |
| TP53 | chr17:7673767-7673767 | E285K | C>T | Single Nucleotide Variants (SNVs) | COSMIC(CRC,Breast) | No | |  | |  | |  | |  | |  | |  | |  | | | |  | |  | |  | |  | |  | |  | |  | |  | |  | |  | |  | |  | |  | |  | |  | |  | |  | |  | |  | |  | |  | |  | |  | |  | |  | |  |  |  |
| TP53 | chr17:7673771-7673771 | R280_R283DelInsS | GCGCCGGTCT>G | Deletion | Tissue(HCC) | No | |  | |  | |  | |  | |  | |  | |  | | | |  | |  | |  | |  | |  | |  | |  | |  | |  | |  | |  | |  | |  | |  | |  | |  | |  | |  | |  | |  | |  | |  | |  | |  | |  | |  |  |  |
| TP53 | chr17:7673776-7673776 | R282G | G>C | Single Nucleotide Variants (SNVs) | Tissue(Breast) | No | |  | |  | |  | |  | |  | |  | |  | | | |  | |  | |  | |  | |  | |  | |  | |  | |  | |  | |  | |  | |  | |  | |  | |  | |  | |  | |  | |  | |  | |  | |  | |  | |  | |  |  |  |
| TP53 | chr17:7673776-7673776 | R282W | G>A | Single Nucleotide Variants (SNVs) | Tissue(CRC,Breast,Gastric,HCC)-COSMIC(CRC,Gastric) | No | |  | |  | |  | |  | |  | |  | |  | | | |  | |  | |  | |  | |  | |  | |  | |  | |  | |  | |  | |  | |  | |  | |  | |  | |  | |  | |  | |  | |  | |  | |  | |  | |  | |  |  |  |
| TP53 | chr17:7673778-7673778 | D281G | T>C | Single Nucleotide Variants (SNVs) | Tissue(Lung) | No | |  | |  | |  | |  | |  | |  | |  | | | |  | |  | |  | |  | |  | |  | |  | |  | |  | |  | |  | |  | |  | |  | |  | |  | |  | |  | |  | |  | |  | |  | |  | |  | |  | |  |  |  |
| TP53 | chr17:7673779-7673779 | D281H | C>G | Single Nucleotide Variants (SNVs) | COSMIC(Lung,Breast) | No | |  | |  | |  | |  | |  | |  | |  | | | |  | |  | |  | |  | |  | |  | |  | |  | |  | |  | |  | |  | |  | |  | |  | |  | |  | |  | |  | |  | |  | |  | |  | |  | |  | |  |  |  |
| TP53 | chr17:7673780-7673780 | R280S | T>G | Single Nucleotide Variants (SNVs) | Tissue(Breast) | No | |  | |  | |  | |  | |  | |  | |  | | | |  | |  | |  | |  | |  | |  | |  | |  | |  | |  | |  | |  | |  | |  | |  | |  | |  | |  | |  | |  | |  | |  | |  | |  | |  | |  |  |  |
| TP53 | chr17:7673781-7673781 | R280K | C>T | Single Nucleotide Variants (SNVs) | Tissue(Breast) | No | |  | |  | |  | |  | |  | |  | |  | | | |  | |  | |  | |  | |  | |  | |  | |  | |  | |  | |  | |  | |  | |  | |  | |  | |  | |  | |  | |  | |  | |  | |  | |  | |  | |  |  |  |
| TP53 | chr17:7673781-7673781 | R280T | C>G | Single Nucleotide Variants (SNVs) | COSMIC(Lung,Breast) | No | |  | |  | |  | |  | |  | |  | |  | | | |  | |  | |  | |  | |  | |  | |  | |  | |  | |  | |  | |  | |  | |  | |  | |  | |  | |  | |  | |  | |  | |  | |  | |  | |  | |  |  |  |
| TP53 | chr17:7673787-7673787 | P278L | G>A | Single Nucleotide Variants (SNVs) | Tissue(Lung,CRC)-COSMIC(Lung,CRC,Breast) | No | |  | |  | |  | |  | |  | |  | |  | | | |  | |  | |  | |  | |  | |  | |  | |  | |  | |  | |  | |  | |  | |  | |  | |  | |  | |  | |  | |  | |  | |  | |  | |  | |  | |  |  |  |
| TP53 | chr17:7673787-7673787 | P278R | G>C | Single Nucleotide Variants (SNVs) | Tissue(Lung) | No | |  | |  | |  | |  | |  | |  | |  | | | |  | |  | |  | |  | |  | |  | |  | |  | |  | |  | |  | |  | |  | |  | |  | |  | |  | |  | |  | |  | |  | |  | |  | |  | |  | |  |  |  |
| TP53 | chr17:7673788-7673788 | P278A | G>C | Single Nucleotide Variants (SNVs) | Tissue(Breast) | No | |  | |  | |  | |  | |  | |  | |  | | | |  | |  | |  | |  | |  | |  | |  | |  | |  | |  | |  | |  | |  | |  | |  | |  | |  | |  | |  | |  | |  | |  | |  | |  | |  | |  |  |  |
| TP53 | chr17:7673788-7673788 | P278S | G>A | Single Nucleotide Variants (SNVs) | Tissue(CRC)-COSMIC(Lung,Breast) | No | |  | |  | |  | |  | |  | |  | |  | | | |  | |  | |  | |  | |  | |  | |  | |  | |  | |  | |  | |  | |  | |  | |  | |  | |  | |  | |  | |  | |  | |  | |  | |  | |  | |  |  |  |
| TP53 | chr17:7673789-7673789 | C277* | A>T | Single Nucleotide Variants (SNVs) | COSMIC(Lung) | No | |  | |  | |  | |  | |  | |  | |  | | | |  | |  | |  | |  | |  | |  | |  | |  | |  | |  | |  | |  | |  | |  | |  | |  | |  | |  | |  | |  | |  | |  | |  | |  | |  | |  |  |  |
| TP53 | chr17:7673790-7673790 | C277F | C>A | Single Nucleotide Variants (SNVs) | Tissue(Lung) | No | |  | |  | |  | |  | |  | |  | |  | | | |  | |  | |  | |  | |  | |  | |  | |  | |  | |  | |  | |  | |  | |  | |  | |  | |  | |  | |  | |  | |  | |  | |  | |  | |  | |  |  |  |
| TP53 | chr17:7673791-7673791 | C277G | A>C | Single Nucleotide Variants (SNVs) | Tissue(Gastric)-COSMIC(Lung) | No | |  | |  | |  | |  | |  | |  | |  | | | |  | |  | |  | |  | |  | |  | |  | |  | |  | |  | |  | |  | |  | |  | |  | |  | |  | |  | |  | |  | |  | |  | |  | |  | |  | |  |  |  |
| TP53 | chr17:7673796-7673796 | C275F | C>A | Single Nucleotide Variants (SNVs) | Tissue(HCC) | No | |  | |  | |  | |  | |  | |  | |  | | | |  | |  | |  | |  | |  | |  | |  | |  | |  | |  | |  | |  | |  | |  | |  | |  | |  | |  | |  | |  | |  | |  | |  | |  | |  | |  |  |  |
| TP53 | chr17:7673796-7673796 | C275Y | C>T | Single Nucleotide Variants (SNVs) | Tissue(CRC) | No | |  | |  | |  | |  | |  | |  | |  | | | |  | |  | |  | |  | |  | |  | |  | |  | |  | |  | |  | |  | |  | |  | |  | |  | |  | |  | |  | |  | |  | |  | |  | |  | |  | |  |  |  |
| TP53 | chr17:7673797-7673797 | C275G | A>C | Single Nucleotide Variants (SNVs) | Tissue(Gastric) | No | |  | |  | |  | |  | |  | |  | |  | | | |  | |  | |  | |  | |  | |  | |  | |  | |  | |  | |  | |  | |  | |  | |  | |  | |  | |  | |  | |  | |  | |  | |  | |  | |  | |  |  |  |
| TP53 | chr17:7673800-7673800 | R273Del | CACG>C | Deletion | COSMIC(Breast,Gastric) | No | |  | |  | |  | |  | |  | |  | |  | | | |  | |  | |  | |  | |  | |  | |  | |  | |  | |  | |  | |  | |  | |  | |  | |  | |  | |  | |  | |  | |  | |  | |  | |  | |  | |  |  |  |
| TP53 | chr17:7673801-7673801 | R273dup | A>AACG | Insertion | COSMIC(Gastric) | No | |  | |  | |  | |  | |  | |  | |  | | | |  | |  | |  | |  | |  | |  | |  | |  | |  | |  | |  | |  | |  | |  | |  | |  | |  | |  | |  | |  | |  | |  | |  | |  | |  | |  |  |  |
| TP53 | chr17:7673801-7673801 | R273dup | A>ACCG | Insertion | COSMIC(Breast,Gastric) | No | |  | |  | |  | |  | |  | |  | |  | | | |  | |  | |  | |  | |  | |  | |  | |  | |  | |  | |  | |  | |  | |  | |  | |  | |  | |  | |  | |  | |  | |  | |  | |  | |  | |  |  |  |
| TP53 | chr17:7673801-7673801 | R273dup | A>ACCT | Insertion | COSMIC(Breast,Gastric) | No | |  | |  | |  | |  | |  | |  | |  | | | |  | |  | |  | |  | |  | |  | |  | |  | |  | |  | |  | |  | |  | |  | |  | |  | |  | |  | |  | |  | |  | |  | |  | |  | |  | |  |  |  |
| TP53 | chr17:7673801-7673801 | R273dup | A>AGCG | Insertion | COSMIC(Breast,Gastric) | No | |  | |  | |  | |  | |  | |  | |  | | | |  | |  | |  | |  | |  | |  | |  | |  | |  | |  | |  | |  | |  | |  | |  | |  | |  | |  | |  | |  | |  | |  | |  | |  | |  | |  |  |  |
| TP53 | chr17:7673801-7673801 | R273dup | A>ATCG | Insertion | COSMIC(Breast,Gastric) | No | |  | |  | |  | |  | |  | |  | |  | | | |  | |  | |  | |  | |  | |  | |  | |  | |  | |  | |  | |  | |  | |  | |  | |  | |  | |  | |  | |  | |  | |  | |  | |  | |  | |  |  |  |
| TP53 | chr17:7673801-7673801 | R273dup | A>ATCT | Insertion | COSMIC(Breast,Gastric) | No | |  | |  | |  | |  | |  | |  | |  | | | |  | |  | |  | |  | |  | |  | |  | |  | |  | |  | |  | |  | |  | |  | |  | |  | |  | |  | |  | |  | |  | |  | |  | |  | |  | |  |  |  |
| TP53 | chr17:7673801-7673801 | R273Q | AC>CT | Multi- nucleotide variants (MNVs) | COSMIC(Breast,Gastric) | No | |  | |  | |  | |  | |  | |  | |  | | | |  | |  | |  | |  | |  | |  | |  | |  | |  | |  | |  | |  | |  | |  | |  | |  | |  | |  | |  | |  | |  | |  | |  | |  | |  | |  |  |  |
| TP53 | chr17:7673801-7673801 | R273Q | AC>TT | Multi- nucleotide variants (MNVs) | COSMIC(Breast,Gastric) | No | |  | |  | |  | |  | |  | |  | |  | | | |  | |  | |  | |  | |  | |  | |  | |  | |  | |  | |  | |  | |  | |  | |  | |  | |  | |  | |  | |  | |  | |  | |  | |  | |  | |  |  |  |
| TP53 | chr17:7673802-7673802 | R273F | CG>AA | Multi- nucleotide variants (MNVs) | COSMIC(Breast,Gastric) | No | |  | |  | |  | |  | |  | |  | |  | | | |  | |  | |  | |  | |  | |  | |  | |  | |  | |  | |  | |  | |  | |  | |  | |  | |  | |  | |  | |  | |  | |  | |  | |  | |  | |  |  |  |
| TP53 | chr17:7673802-7673802 | R273H | C>T | Single Nucleotide Variants (SNVs) | COSMIC(Breast,Gastric,Lung,CRC)-Tissue(CRC,Gastric) | No | |  | |  | |  | |  | |  | |  | |  | | | |  | |  | |  | |  | |  | |  | |  | |  | |  | |  | |  | |  | |  | |  | |  | |  | |  | |  | |  | |  | |  | |  | |  | |  | |  | |  |  |  |
| TP53 | chr17:7673802-7673802 | R273L | C>A | Single Nucleotide Variants (SNVs) | COSMIC(Breast,Gastric)-Tissue(Lung,Breast) | No | |  | |  | |  | |  | |  | |  | |  | | | |  | |  | |  | |  | |  | |  | |  | |  | |  | |  | |  | |  | |  | |  | |  | |  | |  | |  | |  | |  | |  | |  | |  | |  | |  | |  |  |  |
| TP53 | chr17:7673802-7673802 | R273P | C>G | Single Nucleotide Variants (SNVs) | COSMIC(Breast,Gastric) | No | |  | |  | |  | |  | |  | |  | |  | | | |  | |  | |  | |  | |  | |  | |  | |  | |  | |  | |  | |  | |  | |  | |  | |  | |  | |  | |  | |  | |  | |  | |  | |  | |  | |  |  |  |
| TP53 | chr17:7673802-7673802 | R273S | CG>GA | Multi- nucleotide variants (MNVs) | COSMIC(Breast,Gastric) | No | |  | |  | |  | |  | |  | |  | |  | | | |  | |  | |  | |  | |  | |  | |  | |  | |  | |  | |  | |  | |  | |  | |  | |  | |  | |  | |  | |  | |  | |  | |  | |  | |  | |  |  |  |
| TP53 | chr17:7673802-7673802 | R273V | CG>AC | Multi- nucleotide variants (MNVs) | COSMIC(Breast,Gastric) | No | |  | |  | |  | |  | |  | |  | |  | | | |  | |  | |  | |  | |  | |  | |  | |  | |  | |  | |  | |  | |  | |  | |  | |  | |  | |  | |  | |  | |  | |  | |  | |  | |  | |  |  |  |
| TP53 | chr17:7673802-7673802 | R273Y | CG>TA | Multi- nucleotide variants (MNVs) | COSMIC(Breast,Gastric) | No | |  | |  | |  | |  | |  | |  | |  | | | |  | |  | |  | |  | |  | |  | |  | |  | |  | |  | |  | |  | |  | |  | |  | |  | |  | |  | |  | |  | |  | |  | |  | |  | |  | |  |  |  |
| TP53 | chr17:7673803-7673803 | R273C | G>A | Single Nucleotide Variants (SNVs) | COSMIC(Breast,CRC,Gastric) | No | |  | |  | |  | |  | |  | |  | |  | | | |  | |  | |  | |  | |  | |  | |  | |  | |  | |  | |  | |  | |  | |  | |  | |  | |  | |  | |  | |  | |  | |  | |  | |  | |  | |  |  |  |
| TP53 | chr17:7673803-7673803 | R273G | G>C | Single Nucleotide Variants (SNVs) | COSMIC(Breast,Gastric) | No | |  | |  | |  | |  | |  | |  | |  | | | |  | |  | |  | |  | |  | |  | |  | |  | |  | |  | |  | |  | |  | |  | |  | |  | |  | |  | |  | |  | |  | |  | |  | |  | |  | |  |  |  |
| TP53 | chr17:7673803-7673803 | R273S | G>T | Single Nucleotide Variants (SNVs) | COSMIC(Breast,Gastric) | No | |  | |  | |  | |  | |  | |  | |  | | | |  | |  | |  | |  | |  | |  | |  | |  | |  | |  | |  | |  | |  | |  | |  | |  | |  | |  | |  | |  | |  | |  | |  | |  | |  | |  |  |  |
| TP53 | chr17:7673806-7673806 | V272M | C>T | Single Nucleotide Variants (SNVs) | COSMIC(CRC,Breast) | No | |  | |  | |  | |  | |  | |  | |  | | | |  | |  | |  | |  | |  | |  | |  | |  | |  | |  | |  | |  | |  | |  | |  | |  | |  | |  | |  | |  | |  | |  | |  | |  | |  | |  |  |  |
| TP53 | chr17:7673812-7673812 | F270V | A>C | Single Nucleotide Variants (SNVs) | Tissue(CRC) | No | |  | |  | |  | |  | |  | |  | |  | | | |  | |  | |  | |  | |  | |  | |  | |  | |  | |  | |  | |  | |  | |  | |  | |  | |  | |  | |  | |  | |  | |  | |  | |  | |  | |  |  |  |
| TP53 | chr17:7674216-7674216 | R249S | C>A | Single Nucleotide Variants (SNVs) | COSMIC(Breast,HCC,Lung)-Tissue(HCC) | No | |  | |  | |  | |  | |  | |  | |  | | | |  | |  | |  | |  | |  | |  | |  | |  | |  | |  | |  | |  | |  | |  | |  | |  | |  | |  | |  | |  | |  | |  | |  | |  | |  | |  |  |  |
| TP53 | chr17:7674216-7674216 | R249S | C>G | Single Nucleotide Variants (SNVs) | COSMIC(Breast,HCC) | No | |  | |  | |  | |  | |  | |  | |  | | | |  | |  | |  | |  | |  | |  | |  | |  | |  | |  | |  | |  | |  | |  | |  | |  | |  | |  | |  | |  | |  | |  | |  | |  | |  | |  |  |  |
| TP53 | chr17:7674217-7674217 | R249S | CT>GA | Multi- nucleotide variants (MNVs) | COSMIC(Breast,HCC) | No | |  | |  | |  | |  | |  | |  | |  | | | |  | |  | |  | |  | |  | |  | |  | |  | |  | |  | |  | |  | |  | |  | |  | |  | |  | |  | |  | |  | |  | |  | |  | |  | |  | |  |  |  |
| TP53 | chr17:7674218-7674218 | R249W | T>A | Single Nucleotide Variants (SNVs) | Tissue(Lung) | No | |  | |  | |  | |  | |  | |  | |  | | | |  | |  | |  | |  | |  | |  | |  | |  | |  | |  | |  | |  | |  | |  | |  | |  | |  | |  | |  | |  | |  | |  | |  | |  | |  | |  |  |  |
| TP53 | chr17:7674219-7674219 | R248* | CCG>TCA | Multi- nucleotide variants (MNVs) | COSMIC(Breast,Gastric) | No | |  | |  | |  | |  | |  | |  | |  | | | |  | |  | |  | |  | |  | |  | |  | |  | |  | |  | |  | |  | |  | |  | |  | |  | |  | |  | |  | |  | |  | |  | |  | |  | |  | |  |  |  |
| TP53 | chr17:7674219-7674219 | R248C | CCG>ACA | Multi- nucleotide variants (MNVs) | COSMIC(Breast,Gastric) | No | |  | |  | |  | |  | |  | |  | |  | | | |  | |  | |  | |  | |  | |  | |  | |  | |  | |  | |  | |  | |  | |  | |  | |  | |  | |  | |  | |  | |  | |  | |  | |  | |  | |  |  |  |
| TP53 | chr17:7674219-7674219 | R248C | CCG>GCA | Multi- nucleotide variants (MNVs) | COSMIC(Breast,Gastric) | No | |  | |  | |  | |  | |  | |  | |  | | | |  | |  | |  | |  | |  | |  | |  | |  | |  | |  | |  | |  | |  | |  | |  | |  | |  | |  | |  | |  | |  | |  | |  | |  | |  | |  |  |  |
| TP53 | chr17:7674220-7674220 | R248* | CG>TA | Multi- nucleotide variants (MNVs) | COSMIC(Breast,Gastric) | No | |  | |  | |  | |  | |  | |  | |  | | | |  | |  | |  | |  | |  | |  | |  | |  | |  | |  | |  | |  | |  | |  | |  | |  | |  | |  | |  | |  | |  | |  | |  | |  | |  | |  |  |  |
| TP53 | chr17:7674220-7674220 | R248L | C>A | Single Nucleotide Variants (SNVs) | COSMIC(Breast,Gastric,Lung) | No | |  | |  | |  | |  | |  | |  | |  | | | |  | |  | |  | |  | |  | |  | |  | |  | |  | |  | |  | |  | |  | |  | |  | |  | |  | |  | |  | |  | |  | |  | |  | |  | |  | |  |  |  |
| TP53 | chr17:7674220-7674220 | R248P | C>G | Single Nucleotide Variants (SNVs) | COSMIC(Breast,Gastric) | No | |  | |  | |  | |  | |  | |  | |  | | | |  | |  | |  | |  | |  | |  | |  | |  | |  | |  | |  | |  | |  | |  | |  | |  | |  | |  | |  | |  | |  | |  | |  | |  | |  | |  |  |  |
| TP53 | chr17:7674220-7674220 | R248Q | C>T | Single Nucleotide Variants (SNVs) | COSMIC(Breast,Gastric,CRC)-Tissue(CRC,Breast,Gastric) | No | |  | |  | |  | |  | |  | |  | |  | | | |  | |  | |  | |  | |  | |  | |  | |  | |  | |  | |  | |  | |  | |  | |  | |  | |  | |  | |  | |  | |  | |  | |  | |  | |  | |  |  |  |
| TP53 | chr17:7674221-7674221 | R248G | G>C | Single Nucleotide Variants (SNVs) | COSMIC(Gastric)-Tissue(Breast) | No | |  | |  | |  | |  | |  | |  | |  | | | |  | |  | |  | |  | |  | |  | |  | |  | |  | |  | |  | |  | |  | |  | |  | |  | |  | |  | |  | |  | |  | |  | |  | |  | |  | |  |  |  |
| TP53 | chr17:7674221-7674221 | R248W | G>A | Single Nucleotide Variants (SNVs) | COSMIC(Breast,Gastric,)-Tissue(Lung,CRC,Gastric) | No | |  | |  | |  | |  | |  | |  | |  | | | |  | |  | |  | |  | |  | |  | |  | |  | |  | |  | |  | |  | |  | |  | |  | |  | |  | |  | |  | |  | |  | |  | |  | |  | |  | |  |  |  |
| TP53 | chr17:7674227-7674227 | G245Del | TGCC>T | Deletion | COSMIC(Breast,Gastric) | No | |  | |  | |  | |  | |  | |  | |  | | | |  | |  | |  | |  | |  | |  | |  | |  | |  | |  | |  | |  | |  | |  | |  | |  | |  | |  | |  | |  | |  | |  | |  | |  | |  | |  |  |  |
| TP53 | chr17:7674227-7674227 | M246V | T>C | Single Nucleotide Variants (SNVs) | COSMIC(Lung,HCC) | No | |  | |  | |  | |  | |  | |  | |  | | | |  | |  | |  | |  | |  | |  | |  | |  | |  | |  | |  | |  | |  | |  | |  | |  | |  | |  | |  | |  | |  | |  | |  | |  | |  | |  |  |  |
| TP53 | chr17:7674228-7674228 | G245dup | G>GACC | Insertion | COSMIC(Breast,Gastric) | No | |  | |  | |  | |  | |  | |  | |  | | | |  | |  | |  | |  | |  | |  | |  | |  | |  | |  | |  | |  | |  | |  | |  | |  | |  | |  | |  | |  | |  | |  | |  | |  | |  | |  |  |  |
| TP53 | chr17:7674228-7674228 | G245dup | G>GCCC | Insertion | COSMIC(Breast,Gastric) | No | |  | |  | |  | |  | |  | |  | |  | | | |  | |  | |  | |  | |  | |  | |  | |  | |  | |  | |  | |  | |  | |  | |  | |  | |  | |  | |  | |  | |  | |  | |  | |  | |  | |  |  |  |
| TP53 | chr17:7674228-7674228 | G245dup | G>GGCC | Insertion | COSMIC(Breast,Gastric) | No | |  | |  | |  | |  | |  | |  | |  | | | |  | |  | |  | |  | |  | |  | |  | |  | |  | |  | |  | |  | |  | |  | |  | |  | |  | |  | |  | |  | |  | |  | |  | |  | |  | |  |  |  |
| TP53 | chr17:7674228-7674228 | G245dup | G>GTCC | Insertion | COSMIC(Breast,Gastric) | No | |  | |  | |  | |  | |  | |  | |  | | | |  | |  | |  | |  | |  | |  | |  | |  | |  | |  | |  | |  | |  | |  | |  | |  | |  | |  | |  | |  | |  | |  | |  | |  | |  | |  |  |  |
| TP53 | chr17:7674228-7674228 | G245E | GC>CT | Multi- nucleotide variants (MNVs) | COSMIC(Breast,Gastric) | No | |  | |  | |  | |  | |  | |  | |  | | | |  | |  | |  | |  | |  | |  | |  | |  | |  | |  | |  | |  | |  | |  | |  | |  | |  | |  | |  | |  | |  | |  | |  | |  | |  | |  |  |  |
| TP53 | chr17:7674228-7674228 | G245E | GC>TT | Multi- nucleotide variants (MNVs) | COSMIC(Breast,Gastric) | No | |  | |  | |  | |  | |  | |  | |  | | | |  | |  | |  | |  | |  | |  | |  | |  | |  | |  | |  | |  | |  | |  | |  | |  | |  | |  | |  | |  | |  | |  | |  | |  | |  | |  |  |  |
| TP53 | chr17:7674228-7674228 | G245L | GCC>CAA | Multi- nucleotide variants (MNVs) | COSMIC(Breast,Gastric) | No | |  | |  | |  | |  | |  | |  | |  | | | |  | |  | |  | |  | |  | |  | |  | |  | |  | |  | |  | |  | |  | |  | |  | |  | |  | |  | |  | |  | |  | |  | |  | |  | |  | |  |  |  |
| TP53 | chr17:7674228-7674228 | G245L | GCC>TAA | Multi- nucleotide variants (MNVs) | COSMIC(Breast,Gastric) | No | |  | |  | |  | |  | |  | |  | |  | | | |  | |  | |  | |  | |  | |  | |  | |  | |  | |  | |  | |  | |  | |  | |  | |  | |  | |  | |  | |  | |  | |  | |  | |  | |  | |  |  |  |
| TP53 | chr17:7674228-7674228 | G245R | GCC>CCT | Multi- nucleotide variants (MNVs) | COSMIC(Breast,Gastric) | No | |  | |  | |  | |  | |  | |  | |  | | | |  | |  | |  | |  | |  | |  | |  | |  | |  | |  | |  | |  | |  | |  | |  | |  | |  | |  | |  | |  | |  | |  | |  | |  | |  | |  |  |  |
| TP53 | chr17:7674228-7674228 | G245R | GCC>TCT | Multi- nucleotide variants (MNVs) | COSMIC(Breast,Gastric) | No | |  | |  | |  | |  | |  | |  | |  | | | |  | |  | |  | |  | |  | |  | |  | |  | |  | |  | |  | |  | |  | |  | |  | |  | |  | |  | |  | |  | |  | |  | |  | |  | |  | |  |  |  |
| TP53 | chr17:7674229-7674229 | G245A | C>G | Single Nucleotide Variants (SNVs) | COSMIC(Breast,Gastric) | No | |  | |  | |  | |  | |  | |  | |  | | | |  | |  | |  | |  | |  | |  | |  | |  | |  | |  | |  | |  | |  | |  | |  | |  | |  | |  | |  | |  | |  | |  | |  | |  | |  | |  |  |  |
| TP53 | chr17:7674229-7674229 | G245D | C>T | Single Nucleotide Variants (SNVs) | COSMIC(Breast,Gastric,Lung,CRC) | No | |  | |  | |  | |  | |  | |  | |  | | | |  | |  | |  | |  | |  | |  | |  | |  | |  | |  | |  | |  | |  | |  | |  | |  | |  | |  | |  | |  | |  | |  | |  | |  | |  | |  |  |  |
| TP53 | chr17:7674229-7674229 | G245F | CC>AA | Multi- nucleotide variants (MNVs) | COSMIC(Breast,Gastric) | No | |  | |  | |  | |  | |  | |  | |  | | | |  | |  | |  | |  | |  | |  | |  | |  | |  | |  | |  | |  | |  | |  | |  | |  | |  | |  | |  | |  | |  | |  | |  | |  | |  | |  |  |  |
| TP53 | chr17:7674229-7674229 | G245H | CC>TG | Multi- nucleotide variants (MNVs) | COSMIC(Breast,Gastric) | No | |  | |  | |  | |  | |  | |  | |  | | | |  | |  | |  | |  | |  | |  | |  | |  | |  | |  | |  | |  | |  | |  | |  | |  | |  | |  | |  | |  | |  | |  | |  | |  | |  | |  |  |  |
| TP53 | chr17:7674229-7674229 | G245L | CC>AG | Multi- nucleotide variants (MNVs) | COSMIC(Breast,Gastric) | No | |  | |  | |  | |  | |  | |  | |  | | | |  | |  | |  | |  | |  | |  | |  | |  | |  | |  | |  | |  | |  | |  | |  | |  | |  | |  | |  | |  | |  | |  | |  | |  | |  | |  |  |  |
| TP53 | chr17:7674229-7674229 | G245N | CC>TT | Multi- nucleotide variants (MNVs) | COSMIC(Breast,Gastric) | No | |  | |  | |  | |  | |  | |  | |  | | | |  | |  | |  | |  | |  | |  | |  | |  | |  | |  | |  | |  | |  | |  | |  | |  | |  | |  | |  | |  | |  | |  | |  | |  | |  | |  |  |  |
| TP53 | chr17:7674229-7674229 | G245P | CC>GG | Multi- nucleotide variants (MNVs) | COSMIC(Breast,Gastric) | No | |  | |  | |  | |  | |  | |  | |  | | | |  | |  | |  | |  | |  | |  | |  | |  | |  | |  | |  | |  | |  | |  | |  | |  | |  | |  | |  | |  | |  | |  | |  | |  | |  | |  |  |  |
| TP53 | chr17:7674229-7674229 | G245S | CC>GA | Multi- nucleotide variants (MNVs) | COSMIC(Breast,Gastric,HCC) | No | |  | |  | |  | |  | |  | |  | |  | | | |  | |  | |  | |  | |  | |  | |  | |  | |  | |  | |  | |  | |  | |  | |  | |  | |  | |  | |  | |  | |  | |  | |  | |  | |  | |  |  |  |
| TP53 | chr17:7674229-7674229 | G245V | C>A | Single Nucleotide Variants (SNVs) | COSMIC(Breast,Gastric) | No | |  | |  | |  | |  | |  | |  | |  | | | |  | |  | |  | |  | |  | |  | |  | |  | |  | |  | |  | |  | |  | |  | |  | |  | |  | |  | |  | |  | |  | |  | |  | |  | |  | |  |  |  |
| TP53 | chr17:7674230-7674230 | G245C | C>A | Single Nucleotide Variants (SNVs) | COSMIC(Breast,Gastric)-Tissue(Lung) | No | |  | |  | |  | |  | |  | |  | |  | | | |  | |  | |  | |  | |  | |  | |  | |  | |  | |  | |  | |  | |  | |  | |  | |  | |  | |  | |  | |  | |  | |  | |  | |  | |  | |  |  |  |
| TP53 | chr17:7674230-7674230 | G245R | C>G | Single Nucleotide Variants (SNVs) | COSMIC(Breast,Gastric) | No | |  | |  | |  | |  | |  | |  | |  | | | |  | |  | |  | |  | |  | |  | |  | |  | |  | |  | |  | |  | |  | |  | |  | |  | |  | |  | |  | |  | |  | |  | |  | |  | |  | |  |  |  |
| TP53 | chr17:7674230-7674230 | G245S | C>T | Single Nucleotide Variants (SNVs) | COSMIC(Breast,Gastric,HCC,CRC)-Tissue(CRC,Gastric) | No | |  | |  | |  | |  | |  | |  | |  | | | |  | |  | |  | |  | |  | |  | |  | |  | |  | |  | |  | |  | |  | |  | |  | |  | |  | |  | |  | |  | |  | |  | |  | |  | |  | |  |  |  |
| TP53 | chr17:7674232-7674232 | G244V | C>A | Single Nucleotide Variants (SNVs) | Tissue(Breast) | No | |  | |  | |  | |  | |  | |  | |  | | | |  | |  | |  | |  | |  | |  | |  | |  | |  | |  | |  | |  | |  | |  | |  | |  | |  | |  | |  | |  | |  | |  | |  | |  | |  | |  |  |  |
| TP53 | chr17:7674233-7674233 | G244C | C>A | Single Nucleotide Variants (SNVs) | Tissue(Lung) | No | |  | |  | |  | |  | |  | |  | |  | | | |  | |  | |  | |  | |  | |  | |  | |  | |  | |  | |  | |  | |  | |  | |  | |  | |  | |  | |  | |  | |  | |  | |  | |  | |  | |  |  |  |
| TP53 | chr17:7674238-7674238 | C242S | C>G | Single Nucleotide Variants (SNVs) | Tissue(Lung) | No | |  | |  | |  | |  | |  | |  | |  | | | |  | |  | |  | |  | |  | |  | |  | |  | |  | |  | |  | |  | |  | |  | |  | |  | |  | |  | |  | |  | |  | |  | |  | |  | |  | |  |  |  |
| TP53 | chr17:7674238-7674238 | C242Y | C>T | Single Nucleotide Variants (SNVs) | COSMIC(Lung,CRC,Breast) | No | |  | |  | |  | |  | |  | |  | |  | | | |  | |  | |  | |  | |  | |  | |  | |  | |  | |  | |  | |  | |  | |  | |  | |  | |  | |  | |  | |  | |  | |  | |  | |  | |  | |  |  |  |
| TP53 | chr17:7674240-7674240 | C242Afs*5 | GG>G | Deletion | Tissue(Breast)-COSMIC(Breast) | No | |  | |  | |  | |  | |  | |  | |  | | | |  | |  | |  | |  | |  | |  | |  | |  | |  | |  | |  | |  | |  | |  | |  | |  | |  | |  | |  | |  | |  | |  | |  | |  | |  | |  |  |  |
| TP53 | chr17:7674241-7674241 | S241C | G>C | Single Nucleotide Variants (SNVs) | COSMIC(Breast) | No | |  | |  | |  | |  | |  | |  | |  | | | |  | |  | |  | |  | |  | |  | |  | |  | |  | |  | |  | |  | |  | |  | |  | |  | |  | |  | |  | |  | |  | |  | |  | |  | |  | |  |  |  |
| TP53 | chr17:7674241-7674241 | S241F | G>A | Single Nucleotide Variants (SNVs) | COSMIC(Breast) | No | |  | |  | |  | |  | |  | |  | |  | | | |  | |  | |  | |  | |  | |  | |  | |  | |  | |  | |  | |  | |  | |  | |  | |  | |  | |  | |  | |  | |  | |  | |  | |  | |  | |  |  |  |
| TP53 | chr17:7674248-7674248 | N239D | T>C | Single Nucleotide Variants (SNVs) | Tissue(HCC)-COSMIC(Breast) | No | |  | |  | |  | |  | |  | |  | |  | | | |  | |  | |  | |  | |  | |  | |  | |  | |  | |  | |  | |  | |  | |  | |  | |  | |  | |  | |  | |  | |  | |  | |  | |  | |  | |  |  |  |
| TP53 | chr17:7674250-7674250 | C238F | C>A | Single Nucleotide Variants (SNVs) | COSMIC(Breast) | No | |  | |  | |  | |  | |  | |  | |  | | | |  | |  | |  | |  | |  | |  | |  | |  | |  | |  | |  | |  | |  | |  | |  | |  | |  | |  | |  | |  | |  | |  | |  | |  | |  | |  |  |  |
| TP53 | chr17:7674250-7674250 | C238S | C>G | Single Nucleotide Variants (SNVs) | Tissue(Breast) | No | |  | |  | |  | |  | |  | |  | |  | | | |  | |  | |  | |  | |  | |  | |  | |  | |  | |  | |  | |  | |  | |  | |  | |  | |  | |  | |  | |  | |  | |  | |  | |  | |  | |  |  |  |
| TP53 | chr17:7674250-7674250 | C238Y | C>T | Single Nucleotide Variants (SNVs) | COSMIC(Breast) | No | |  | |  | |  | |  | |  | |  | |  | | | |  | |  | |  | |  | |  | |  | |  | |  | |  | |  | |  | |  | |  | |  | |  | |  | |  | |  | |  | |  | |  | |  | |  | |  | |  | |  |  |  |
| TP53 | chr17:7674252-7674252 | M237I | C>A | Single Nucleotide Variants (SNVs) | COSMIC(Breast) | No | |  | |  | |  | |  | |  | |  | |  | | | |  | |  | |  | |  | |  | |  | |  | |  | |  | |  | |  | |  | |  | |  | |  | |  | |  | |  | |  | |  | |  | |  | |  | |  | |  | |  |  |  |
| TP53 | chr17:7674252-7674252 | M237I | C>G | Single Nucleotide Variants (SNVs) | COSMIC(Breast) | No | |  | |  | |  | |  | |  | |  | |  | | | |  | |  | |  | |  | |  | |  | |  | |  | |  | |  | |  | |  | |  | |  | |  | |  | |  | |  | |  | |  | |  | |  | |  | |  | |  | |  |  |  |
| TP53 | chr17:7674252-7674252 | M237I | C>T | Single Nucleotide Variants (SNVs) | Tissue(Breast) | No | |  | |  | |  | |  | |  | |  | |  | | | |  | |  | |  | |  | |  | |  | |  | |  | |  | |  | |  | |  | |  | |  | |  | |  | |  | |  | |  | |  | |  | |  | |  | |  | |  | |  |  |  |
| TP53 | chr17:7674256-7674256 | Y236C | T>C | Single Nucleotide Variants (SNVs) | Tissue(HCC)-COSMIC(Breast) | No | |  | |  | |  | |  | |  | |  | |  | | | |  | |  | |  | |  | |  | |  | |  | |  | |  | |  | |  | |  | |  | |  | |  | |  | |  | |  | |  | |  | |  | |  | |  | |  | |  | |  |  |  |
| TP53 | chr17:7674260-7674260 | N235D | T>C | Single Nucleotide Variants (SNVs) | Tissue(Gastric) | No | |  | |  | |  | |  | |  | |  | |  | | | |  | |  | |  | |  | |  | |  | |  | |  | |  | |  | |  | |  | |  | |  | |  | |  | |  | |  | |  | |  | |  | |  | |  | |  | |  | |  |  |  |
| TP53 | chr17:7674262-7674262 | Y234C | T>C | Single Nucleotide Variants (SNVs) | COSMIC(Breast) | No | |  | |  | |  | |  | |  | |  | |  | | | |  | |  | |  | |  | |  | |  | |  | |  | |  | |  | |  | |  | |  | |  | |  | |  | |  | |  | |  | |  | |  | |  | |  | |  | |  | |  |  |  |
| TP53 | chr17:7674870-7674870 | P219_Y220Del | CATAGGG>C | Deletion | Tissue(Breast) | No | |  | |  | |  | |  | |  | |  | |  | | | |  | |  | |  | |  | |  | |  | |  | |  | |  | |  | |  | |  | |  | |  | |  | |  | |  | |  | |  | |  | |  | |  | |  | |  | |  | |  |  |  |
| TP53 | chr17:7674870-7674870 | Y220Del | CATA>C | Deletion | COSMIC(Breast,Gastric) | No | |  | |  | |  | |  | |  | |  | |  | | | |  | |  | |  | |  | |  | |  | |  | |  | |  | |  | |  | |  | |  | |  | |  | |  | |  | |  | |  | |  | |  | |  | |  | |  | |  | |  |  |  |
| TP53 | chr17:7674871-7674871 | Y220* | A>C | Single Nucleotide Variants (SNVs) | COSMIC(Breast,Gastric) | No | |  | |  | |  | |  | |  | |  | |  | | | |  | |  | |  | |  | |  | |  | |  | |  | |  | |  | |  | |  | |  | |  | |  | |  | |  | |  | |  | |  | |  | |  | |  | |  | |  | |  |  |  |
| TP53 | chr17:7674871-7674871 | Y220* | A>T | Single Nucleotide Variants (SNVs) | COSMIC(Breast,Gastric) | No | |  | |  | |  | |  | |  | |  | |  | | | |  | |  | |  | |  | |  | |  | |  | |  | |  | |  | |  | |  | |  | |  | |  | |  | |  | |  | |  | |  | |  | |  | |  | |  | |  | |  |  |  |
| TP53 | chr17:7674871-7674871 | Y220dup | A>AATA | Insertion | COSMIC(Breast,Gastric) | No | |  | |  | |  | |  | |  | |  | |  | | | |  | |  | |  | |  | |  | |  | |  | |  | |  | |  | |  | |  | |  | |  | |  | |  | |  | |  | |  | |  | |  | |  | |  | |  | |  | |  |  |  |
| TP53 | chr17:7674871-7674871 | Y220dup | A>AGTA | Insertion | COSMIC(Breast,Gastric) | No | |  | |  | |  | |  | |  | |  | |  | | | |  | |  | |  | |  | |  | |  | |  | |  | |  | |  | |  | |  | |  | |  | |  | |  | |  | |  | |  | |  | |  | |  | |  | |  | |  | |  |  |  |
| TP53 | chr17:7674871-7674871 | Y220L | AT>CA | Multi- nucleotide variants (MNVs) | COSMIC(Breast,Gastric) | No | |  | |  | |  | |  | |  | |  | |  | | | |  | |  | |  | |  | |  | |  | |  | |  | |  | |  | |  | |  | |  | |  | |  | |  | |  | |  | |  | |  | |  | |  | |  | |  | |  | |  |  |  |
| TP53 | chr17:7674871-7674871 | Y220L | AT>TA | Multi- nucleotide variants (MNVs) | COSMIC(Breast,Gastric) | No | |  | |  | |  | |  | |  | |  | |  | | | |  | |  | |  | |  | |  | |  | |  | |  | |  | |  | |  | |  | |  | |  | |  | |  | |  | |  | |  | |  | |  | |  | |  | |  | |  | |  |  |  |
| TP53 | chr17:7674871-7674871 | Y220M | ATA>CAT | Multi- nucleotide variants (MNVs) | COSMIC(Breast,Gastric) | No | |  | |  | |  | |  | |  | |  | |  | | | |  | |  | |  | |  | |  | |  | |  | |  | |  | |  | |  | |  | |  | |  | |  | |  | |  | |  | |  | |  | |  | |  | |  | |  | |  | |  |  |  |
| TP53 | chr17:7674871-7674871 | Y220R | ATA>CCT | Multi- nucleotide variants (MNVs) | COSMIC(Breast,Gastric) | No | |  | |  | |  | |  | |  | |  | |  | | | |  | |  | |  | |  | |  | |  | |  | |  | |  | |  | |  | |  | |  | |  | |  | |  | |  | |  | |  | |  | |  | |  | |  | |  | |  | |  |  |  |
| TP53 | chr17:7674871-7674871 | Y220R | ATA>TCT | Multi- nucleotide variants (MNVs) | COSMIC(Breast,Gastric) | No | |  | |  | |  | |  | |  | |  | |  | | | |  | |  | |  | |  | |  | |  | |  | |  | |  | |  | |  | |  | |  | |  | |  | |  | |  | |  | |  | |  | |  | |  | |  | |  | |  | |  |  |  |
| TP53 | chr17:7674872-7674872 | Y220C | T>C | Single Nucleotide Variants (SNVs) | COSMIC(Breast,Gastric,HCC,Lung)-Tissue(Gastric) | No | |  | |  | |  | |  | |  | |  | |  | | | |  | |  | |  | |  | |  | |  | |  | |  | |  | |  | |  | |  | |  | |  | |  | |  | |  | |  | |  | |  | |  | |  | |  | |  | |  | |  |  |  |
| TP53 | chr17:7674872-7674872 | Y220L | TA>AG | Multi- nucleotide variants (MNVs) | COSMIC(Breast,Gastric) | No | |  | |  | |  | |  | |  | |  | |  | | | |  | |  | |  | |  | |  | |  | |  | |  | |  | |  | |  | |  | |  | |  | |  | |  | |  | |  | |  | |  | |  | |  | |  | |  | |  | |  |  |  |
| TP53 | chr17:7674872-7674872 | Y220R | TA>CG | Multi- nucleotide variants (MNVs) | COSMIC(Breast,Gastric) | No | |  | |  | |  | |  | |  | |  | |  | | | |  | |  | |  | |  | |  | |  | |  | |  | |  | |  | |  | |  | |  | |  | |  | |  | |  | |  | |  | |  | |  | |  | |  | |  | |  | |  |  |  |
| TP53 | chr17:7674872-7674872 | Y220S | T>G | Single Nucleotide Variants (SNVs) | COSMIC(Breast,Gastric)-Tissue(Lung) | No | |  | |  | |  | |  | |  | |  | |  | | | |  | |  | |  | |  | |  | |  | |  | |  | |  | |  | |  | |  | |  | |  | |  | |  | |  | |  | |  | |  | |  | |  | |  | |  | |  | |  |  |  |
| TP53 | chr17:7674872-7674872 | Y220S | TA>CT | Multi- nucleotide variants (MNVs) | COSMIC(Breast,Gastric) | No | |  | |  | |  | |  | |  | |  | |  | | | |  | |  | |  | |  | |  | |  | |  | |  | |  | |  | |  | |  | |  | |  | |  | |  | |  | |  | |  | |  | |  | |  | |  | |  | |  | |  |  |  |
| TP53 | chr17:7674873-7674873 | Y220D | A>C | Single Nucleotide Variants (SNVs) | COSMIC(Breast,Gastric) | No | |  | |  | |  | |  | |  | |  | |  | | | |  | |  | |  | |  | |  | |  | |  | |  | |  | |  | |  | |  | |  | |  | |  | |  | |  | |  | |  | |  | |  | |  | |  | |  | |  | |  |  |  |
| TP53 | chr17:7674873-7674873 | Y220H | A>G | Single Nucleotide Variants (SNVs) | COSMIC(Breast,Gastric)-Tissue(Lung) | No | |  | |  | |  | |  | |  | |  | |  | | | |  | |  | |  | |  | |  | |  | |  | |  | |  | |  | |  | |  | |  | |  | |  | |  | |  | |  | |  | |  | |  | |  | |  | |  | |  | |  |  |  |
| TP53 | chr17:7674873-7674873 | Y220N | A>T | Single Nucleotide Variants (SNVs) | COSMIC(Gastric)-Tissue(Breast) | No | |  | |  | |  | |  | |  | |  | |  | | | |  | |  | |  | |  | |  | |  | |  | |  | |  | |  | |  | |  | |  | |  | |  | |  | |  | |  | |  | |  | |  | |  | |  | |  | |  | |  |  |  |
| TP53 | chr17:7674877-7674877 | V218Gfs* | CA>C | Deletion | Tissue(Lung) | No | |  | |  | |  | |  | |  | |  | |  | | | |  | |  | |  | |  | |  | |  | |  | |  | |  | |  | |  | |  | |  | |  | |  | |  | |  | |  | |  | |  | |  | |  | |  | |  | |  | |  |  |  |
| TP53 | chr17:7674878-7674878 | V218Cfs*29 | AC>A | Deletion | Tissue(Breast) | No | |  | |  | |  | |  | |  | |  | |  | | | |  | |  | |  | |  | |  | |  | |  | |  | |  | |  | |  | |  | |  | |  | |  | |  | |  | |  | |  | |  | |  | |  | |  | |  | |  | |  |  |  |
| TP53 | chr17:7674885-7674885 | V216L | C>A | Single Nucleotide Variants (SNVs) | Tissue(Lung) | No | |  | |  | |  | |  | |  | |  | |  | | | |  | |  | |  | |  | |  | |  | |  | |  | |  | |  | |  | |  | |  | |  | |  | |  | |  | |  | |  | |  | |  | |  | |  | |  | |  | |  |  |  |
| TP53 | chr17:7674885-7674885 | V216M | C>T | Single Nucleotide Variants (SNVs) | Tissue(CRC)-COSMIC(Lung,CRC,Breast) | No | |  | |  | |  | |  | |  | |  | |  | | | |  | |  | |  | |  | |  | |  | |  | |  | |  | |  | |  | |  | |  | |  | |  | |  | |  | |  | |  | |  | |  | |  | |  | |  | |  | |  |  |  |
| TP53 | chr17:7674887-7674887 | S215N | C>T | Single Nucleotide Variants (SNVs) | Tissue(CRC) | No | |  | |  | |  | |  | |  | |  | |  | | | |  | |  | |  | |  | |  | |  | |  | |  | |  | |  | |  | |  | |  | |  | |  | |  | |  | |  | |  | |  | |  | |  | |  | |  | |  | |  |  |  |
| TP53 | chr17:7674890-7674890 | H214R | T>C | Single Nucleotide Variants (SNVs) | COSMIC(Lung,Gastric,HCC) | No | |  | |  | |  | |  | |  | |  | |  | | | |  | |  | |  | |  | |  | |  | |  | |  | |  | |  | |  | |  | |  | |  | |  | |  | |  | |  | |  | |  | |  | |  | |  | |  | |  | |  |  |  |
| TP53 | chr17:7674893-7674893 | R213L | C>A | Single Nucleotide Variants (SNVs) | Tissue(HCC)-COSMIC(CRC,HCC) | No | |  | |  | |  | |  | |  | |  | |  | | | |  | |  | |  | |  | |  | |  | |  | |  | |  | |  | |  | |  | |  | |  | |  | |  | |  | |  | |  | |  | |  | |  | |  | |  | |  | |  |  |  |
| TP53 | chr17:7674894-7674894 | R213* | G>A | Single Nucleotide Variants (SNVs) | Tissue(CRC,Breast)-COSMIC(CRC) | No | |  | |  | |  | |  | |  | |  | |  | | | |  | |  | |  | |  | |  | |  | |  | |  | |  | |  | |  | |  | |  | |  | |  | |  | |  | |  | |  | |  | |  | |  | |  | |  | |  | |  |  |  |
| TP53 | chr17:7674945-7674945 | R196* | G>A | Single Nucleotide Variants (SNVs) | Tissue(CRC)-COSMIC(CRC,Gastric) | No | |  | |  | |  | |  | |  | |  | |  | | | |  | |  | |  | |  | |  | |  | |  | |  | |  | |  | |  | |  | |  | |  | |  | |  | |  | |  | |  | |  | |  | |  | |  | |  | |  | |  |  |  |
| TP53 | chr17:7674947-7674947 | I195T | A>G | Single Nucleotide Variants (SNVs) | Tissue(Gastric) | No | |  | |  | |  | |  | |  | |  | |  | | | |  | |  | |  | |  | |  | |  | |  | |  | |  | |  | |  | |  | |  | |  | |  | |  | |  | |  | |  | |  | |  | |  | |  | |  | |  | |  |  |  |
| TP53 | chr17:7674948-7674948 | I195F | T>A | Single Nucleotide Variants (SNVs) | Tissue(Lung) | No | |  | |  | |  | |  | |  | |  | |  | | | |  | |  | |  | |  | |  | |  | |  | |  | |  | |  | |  | |  | |  | |  | |  | |  | |  | |  | |  | |  | |  | |  | |  | |  | |  | |  |  |  |
| TP53 | chr17:7674950-7674950 | L194R | A>C | Single Nucleotide Variants (SNVs) | Tissue(CRC) | No | |  | |  | |  | |  | |  | |  | |  | | | |  | |  | |  | |  | |  | |  | |  | |  | |  | |  | |  | |  | |  | |  | |  | |  | |  | |  | |  | |  | |  | |  | |  | |  | |  | |  |  |  |
| TP53 | chr17:7674953-7674953 | H193R | T>C | Single Nucleotide Variants (SNVs) | Tissue(Gastric) | No | |  | |  | |  | |  | |  | |  | |  | | | |  | |  | |  | |  | |  | |  | |  | |  | |  | |  | |  | |  | |  | |  | |  | |  | |  | |  | |  | |  | |  | |  | |  | |  | |  | |  |  |  |
| TP53 | chr17:7674954-7674954 | H193Y | G>A | Single Nucleotide Variants (SNVs) | Tissue(Lung) | No | |  | |  | |  | |  | |  | |  | |  | | | |  | |  | |  | |  | |  | |  | |  | |  | |  | |  | |  | |  | |  | |  | |  | |  | |  | |  | |  | |  | |  | |  | |  | |  | |  | |  |  |  |
| TP53 | chr17:7674956-7674956 | P191Del | TGAG>T | Deletion | Tissue(Lung) | No | |  | |  | |  | |  | |  | |  | |  | | | |  | |  | |  | |  | |  | |  | |  | |  | |  | |  | |  | |  | |  | |  | |  | |  | |  | |  | |  | |  | |  | |  | |  | |  | |  | |  |  |  |
| TP53 | chr17:7674957-7674957 | Q192* | G>A | Single Nucleotide Variants (SNVs) | Tissue(Lung) | No | |  | |  | |  | |  | |  | |  | |  | | | |  | |  | |  | |  | |  | |  | |  | |  | |  | |  | |  | |  | |  | |  | |  | |  | |  | |  | |  | |  | |  | |  | |  | |  | |  | |  |  |  |
| TP53 | chr17:7674962-7674962 | P190R | G>C | Single Nucleotide Variants (SNVs) | Tissue(CRC) | No | |  | |  | |  | |  | |  | |  | |  | | | |  | |  | |  | |  | |  | |  | |  | |  | |  | |  | |  | |  | |  | |  | |  | |  | |  | |  | |  | |  | |  | |  | |  | |  | |  | |  |  |  |
| TP53 | chr17:7674963-7674963 | P190T | G>T | Single Nucleotide Variants (SNVs) | Tissue(Lung) | No | |  | |  | |  | |  | |  | |  | |  | | | |  | |  | |  | |  | |  | |  | |  | |  | |  | |  | |  | |  | |  | |  | |  | |  | |  | |  | |  | |  | |  | |  | |  | |  | |  | |  |  |  |
| TP53 | chr17:7675076-7675076 | H179R | T>C | Single Nucleotide Variants (SNVs) | Tissue(Breast,HCC) | No | |  | |  | |  | |  | |  | |  | |  | | | |  | |  | |  | |  | |  | |  | |  | |  | |  | |  | |  | |  | |  | |  | |  | |  | |  | |  | |  | |  | |  | |  | |  | |  | |  | |  |  |  |
| TP53 | chr17:7675077-7675077 | H179D | G>C | Single Nucleotide Variants (SNVs) | Tissue(Breast) | No | |  | |  | |  | |  | |  | |  | |  | | | |  | |  | |  | |  | |  | |  | |  | |  | |  | |  | |  | |  | |  | |  | |  | |  | |  | |  | |  | |  | |  | |  | |  | |  | |  | |  |  |  |
| TP53 | chr17:7675077-7675077 | H179N | G>T | Single Nucleotide Variants (SNVs) | Tissue(Breast) | No | |  | |  | |  | |  | |  | |  | |  | | | |  | |  | |  | |  | |  | |  | |  | |  | |  | |  | |  | |  | |  | |  | |  | |  | |  | |  | |  | |  | |  | |  | |  | |  | |  | |  |  |  |
| TP53 | chr17:7675077-7675077 | H179Y | G>A | Single Nucleotide Variants (SNVs) | Tissue(Gastric)-COSMIC(CRC,Breast) | No | |  | |  | |  | |  | |  | |  | |  | | | |  | |  | |  | |  | |  | |  | |  | |  | |  | |  | |  | |  | |  | |  | |  | |  | |  | |  | |  | |  | |  | |  | |  | |  | |  | |  |  |  |
| TP53 | chr17:7675079-7675079 | H178Tfs*69 | TG>T | Deletion | Tissue(CRC) | No | |  | |  | |  | |  | |  | |  | |  | | | |  | |  | |  | |  | |  | |  | |  | |  | |  | |  | |  | |  | |  | |  | |  | |  | |  | |  | |  | |  | |  | |  | |  | |  | |  | |  |  |  |
| TP53 | chr17:7675085-7675085 | C176F | C>A | Single Nucleotide Variants (SNVs) | Tissue(Gastric,HCC)-COSMIC(CRC,Gastric) | No | |  | |  | |  | |  | |  | |  | |  | | | |  | |  | |  | |  | |  | |  | |  | |  | |  | |  | |  | |  | |  | |  | |  | |  | |  | |  | |  | |  | |  | |  | |  | |  | |  | |  |  |  |
| TP53 | chr17:7675085-7675085 | C176Y | C>T | Single Nucleotide Variants (SNVs) | COSMIC(CRC) | No | |  | |  | |  | |  | |  | |  | |  | | | |  | |  | |  | |  | |  | |  | |  | |  | |  | |  | |  | |  | |  | |  | |  | |  | |  | |  | |  | |  | |  | |  | |  | |  | |  | |  |  |  |
| TP53 | chr17:7675086-7675086 | C176R | A>G | Single Nucleotide Variants (SNVs) | Tissue(Breast,Gastric) | No | |  | |  | |  | |  | |  | |  | |  | | | |  | |  | |  | |  | |  | |  | |  | |  | |  | |  | |  | |  | |  | |  | |  | |  | |  | |  | |  | |  | |  | |  | |  | |  | |  | |  |  |  |
| TP53 | chr17:7675086-7675086 | R175Del | AGCG>A | Deletion | COSMIC(Breast,Gastric) | No | |  | |  | |  | |  | |  | |  | |  | | | |  | |  | |  | |  | |  | |  | |  | |  | |  | |  | |  | |  | |  | |  | |  | |  | |  | |  | |  | |  | |  | |  | |  | |  | |  | |  |  |  |
| TP53 | chr17:7675087-7675087 | R175dup | G>GACG | Insertion | COSMIC(Breast,Gastric) | No | |  | |  | |  | |  | |  | |  | |  | | | |  | |  | |  | |  | |  | |  | |  | |  | |  | |  | |  | |  | |  | |  | |  | |  | |  | |  | |  | |  | |  | |  | |  | |  | |  | |  |  |  |
| TP53 | chr17:7675087-7675087 | R175dup | G>GCCG | Insertion | COSMIC(Breast,Gastric) | No | |  | |  | |  | |  | |  | |  | |  | | | |  | |  | |  | |  | |  | |  | |  | |  | |  | |  | |  | |  | |  | |  | |  | |  | |  | |  | |  | |  | |  | |  | |  | |  | |  | |  |  |  |
| TP53 | chr17:7675087-7675087 | R175dup | G>GCCT | Insertion | COSMIC(Breast,Gastric) | No | |  | |  | |  | |  | |  | |  | |  | | | |  | |  | |  | |  | |  | |  | |  | |  | |  | |  | |  | |  | |  | |  | |  | |  | |  | |  | |  | |  | |  | |  | |  | |  | |  | |  |  |  |
| TP53 | chr17:7675087-7675087 | R175dup | G>GGCG | Insertion | COSMIC(Breast,Gastric) | No | |  | |  | |  | |  | |  | |  | |  | | | |  | |  | |  | |  | |  | |  | |  | |  | |  | |  | |  | |  | |  | |  | |  | |  | |  | |  | |  | |  | |  | |  | |  | |  | |  | |  |  |  |
| TP53 | chr17:7675087-7675087 | R175dup | G>GTCG | Insertion | COSMIC(Breast,Gastric) | No | |  | |  | |  | |  | |  | |  | |  | | | |  | |  | |  | |  | |  | |  | |  | |  | |  | |  | |  | |  | |  | |  | |  | |  | |  | |  | |  | |  | |  | |  | |  | |  | |  | |  |  |  |
| TP53 | chr17:7675087-7675087 | R175dup | G>GTCT | Insertion | COSMIC(Breast,Gastric) | No | |  | |  | |  | |  | |  | |  | |  | | | |  | |  | |  | |  | |  | |  | |  | |  | |  | |  | |  | |  | |  | |  | |  | |  | |  | |  | |  | |  | |  | |  | |  | |  | |  | |  |  |  |
| TP53 | chr17:7675088-7675088 | R175A | CG>GC | Multi- nucleotide variants (MNVs) | COSMIC(Breast,Gastric) | No | |  | |  | |  | |  | |  | |  | |  | | | |  | |  | |  | |  | |  | |  | |  | |  | |  | |  | |  | |  | |  | |  | |  | |  | |  | |  | |  | |  | |  | |  | |  | |  | |  | |  |  |  |
| TP53 | chr17:7675088-7675088 | R175H | C>T | Single Nucleotide Variants (SNVs) | COSMIC(Breast, CRC,Gastric)-Tissue(CRC) | No | |  | |  | |  | |  | |  | |  | |  | | | |  | |  | |  | |  | |  | |  | |  | |  | |  | |  | |  | |  | |  | |  | |  | |  | |  | |  | |  | |  | |  | |  | |  | |  | |  | |  |  |  |
| TP53 | chr17:7675088-7675088 | R175L | C>A | Single Nucleotide Variants (SNVs) | COSMIC(Breast,Gastric) | No | |  | |  | |  | |  | |  | |  | |  | | | |  | |  | |  | |  | |  | |  | |  | |  | |  | |  | |  | |  | |  | |  | |  | |  | |  | |  | |  | |  | |  | |  | |  | |  | |  | |  |  |  |
| TP53 | chr17:7675088-7675088 | R175P | C>G | Single Nucleotide Variants (SNVs) | COSMIC(Breast,Gastric) | No | |  | |  | |  | |  | |  | |  | |  | | | |  | |  | |  | |  | |  | |  | |  | |  | |  | |  | |  | |  | |  | |  | |  | |  | |  | |  | |  | |  | |  | |  | |  | |  | |  | |  |  |  |
| TP53 | chr17:7675088-7675088 | R175S | CG>GA | Multi- nucleotide variants (MNVs) | COSMIC(Breast,Gastric) | No | |  | |  | |  | |  | |  | |  | |  | | | |  | |  | |  | |  | |  | |  | |  | |  | |  | |  | |  | |  | |  | |  | |  | |  | |  | |  | |  | |  | |  | |  | |  | |  | |  | |  |  |  |
| TP53 | chr17:7675089-7675089 | R175C | G>A | Single Nucleotide Variants (SNVs) | COSMIC(Breast,Gastric) | No | |  | |  | |  | |  | |  | |  | |  | | | |  | |  | |  | |  | |  | |  | |  | |  | |  | |  | |  | |  | |  | |  | |  | |  | |  | |  | |  | |  | |  | |  | |  | |  | |  | |  |  |  |
| TP53 | chr17:7675089-7675089 | R175G | G>C | Single Nucleotide Variants (SNVs) | COSMIC(Gastric)-Tissue(Breast) | No | |  | |  | |  | |  | |  | |  | |  | | | |  | |  | |  | |  | |  | |  | |  | |  | |  | |  | |  | |  | |  | |  | |  | |  | |  | |  | |  | |  | |  | |  | |  | |  | |  | |  |  |  |
| TP53 | chr17:7675089-7675089 | R175S | G>T | Single Nucleotide Variants (SNVs) | COSMIC(Breast,Gastric) | No | |  | |  | |  | |  | |  | |  | |  | | | |  | |  | |  | |  | |  | |  | |  | |  | |  | |  | |  | |  | |  | |  | |  | |  | |  | |  | |  | |  | |  | |  | |  | |  | |  | |  |  |  |
| TP53 | chr17:7675095-7675095 | V173* | CA>C | Deletion | Tissue(Gastric) | No | |  | |  | |  | |  | |  | |  | |  | | | |  | |  | |  | |  | |  | |  | |  | |  | |  | |  | |  | |  | |  | |  | |  | |  | |  | |  | |  | |  | |  | |  | |  | |  | |  | |  |  |  |
| TP53 | chr17:7675095-7675095 | V173L | C>A | Single Nucleotide Variants (SNVs) | Tissue(CRC)-COSMIC(CRC,Gastric) | No | |  | |  | |  | |  | |  | |  | |  | | | |  | |  | |  | |  | |  | |  | |  | |  | |  | |  | |  | |  | |  | |  | |  | |  | |  | |  | |  | |  | |  | |  | |  | |  | |  | |  |  |  |
| TP53 | chr17:7675095-7675095 | V173L | C>G | Single Nucleotide Variants (SNVs) | COSMIC(CRC) | No | |  | |  | |  | |  | |  | |  | |  | | | |  | |  | |  | |  | |  | |  | |  | |  | |  | |  | |  | |  | |  | |  | |  | |  | |  | |  | |  | |  | |  | |  | |  | |  | |  | |  |  |  |
| TP53 | chr17:7675095-7675095 | V173M | C>T | Single Nucleotide Variants (SNVs) | Tissue(Gastric)-COSMIC(CRC,Breast) | No | |  | |  | |  | |  | |  | |  | |  | | | |  | |  | |  | |  | |  | |  | |  | |  | |  | |  | |  | |  | |  | |  | |  | |  | |  | |  | |  | |  | |  | |  | |  | |  | |  | |  |  |  |
| TP53 | chr17:7675113-7675113 | Q167* | G>A | Single Nucleotide Variants (SNVs) | Tissue(Lung) | No | |  | |  | |  | |  | |  | |  | |  | | | |  | |  | |  | |  | |  | |  | |  | |  | |  | |  | |  | |  | |  | |  | |  | |  | |  | |  | |  | |  | |  | |  | |  | |  | |  | |  |  |  |
| TP53 | chr17:7675119-7675119 | Q165* | G>A | Single Nucleotide Variants (SNVs) | Tissue(Lung) | No | |  | |  | |  | |  | |  | |  | |  | | | |  | |  | |  | |  | |  | |  | |  | |  | |  | |  | |  | |  | |  | |  | |  | |  | |  | |  | |  | |  | |  | |  | |  | |  | |  | |  |  |  |
| TP53 | chr17:7675137-7675137 | A159P | C>G | Single Nucleotide Variants (SNVs) | COSMIC(Lung,HCC) | No | |  | |  | |  | |  | |  | |  | |  | | | |  | |  | |  | |  | |  | |  | |  | |  | |  | |  | |  | |  | |  | |  | |  | |  | |  | |  | |  | |  | |  | |  | |  | |  | |  | |  |  |  |
| TP53 | chr17:7675139-7675139 | R158H | C>T | Single Nucleotide Variants (SNVs) | COSMIC(Gastric) | No | |  | |  | |  | |  | |  | |  | |  | | | |  | |  | |  | |  | |  | |  | |  | |  | |  | |  | |  | |  | |  | |  | |  | |  | |  | |  | |  | |  | |  | |  | |  | |  | |  | |  |  |  |
| TP53 | chr17:7675139-7675139 | R158L | C>A | Single Nucleotide Variants (SNVs) | COSMIC(Lung) | No | |  | |  | |  | |  | |  | |  | |  | | | |  | |  | |  | |  | |  | |  | |  | |  | |  | |  | |  | |  | |  | |  | |  | |  | |  | |  | |  | |  | |  | |  | |  | |  | |  | |  |  |  |
| TP53 | chr17:7675139-7675139 | R158P | C>G | Single Nucleotide Variants (SNVs) | Tissue(Lung) | No | |  | |  | |  | |  | |  | |  | |  | | | |  | |  | |  | |  | |  | |  | |  | |  | |  | |  | |  | |  | |  | |  | |  | |  | |  | |  | |  | |  | |  | |  | |  | |  | |  | |  |  |  |
| TP53 | chr17:7675143-7675143 | V157F | C>A | Single Nucleotide Variants (SNVs) | COSMIC(Lung,HCC) | No | |  | |  | |  | |  | |  | |  | |  | | | |  | |  | |  | |  | |  | |  | |  | |  | |  | |  | |  | |  | |  | |  | |  | |  | |  | |  | |  | |  | |  | |  | |  | |  | |  | |  |  |  |
| TP53 | chr17:7675144-7675144 | R156Pfs*14 | GC>G | Deletion | Tissue(HCC) | No | |  | |  | |  | |  | |  | |  | |  | | | |  | |  | |  | |  | |  | |  | |  | |  | |  | |  | |  | |  | |  | |  | |  | |  | |  | |  | |  | |  | |  | |  | |  | |  | |  | |  |  |  |
| TP53 | chr17:7675151-7675151 | G154V | C>A | Single Nucleotide Variants (SNVs) | Tissue(Lung) | No | |  | |  | |  | |  | |  | |  | |  | | | |  | |  | |  | |  | |  | |  | |  | |  | |  | |  | |  | |  | |  | |  | |  | |  | |  | |  | |  | |  | |  | |  | |  | |  | |  | |  |  |  |
| TP53 | chr17:7674888-7674888 | S215G | T>C | Single Nucleotide Variants (SNVs) | COSMIC(Gastric) | No | |  | |  | |  | |  | |  | |  | |  | | | |  | |  | |  | |  | |  | |  | |  | |  | |  | |  | |  | |  | |  | |  | |  | |  | |  | |  | |  | |  | |  | |  | |  | |  | |  | |  |  |  |
| TERT | chr5:1295113-1295113 | C228T | G>A | Single Nucleotide Variants (SNVs) | Tissue(Lung,HCC) | No | |  | |  | |  | |  | |  | |  | |  | | | |  | |  | |  | |  | |  | |  | |  | |  | |  | |  | |  | |  | |  | |  | |  | |  | |  | |  | |  | |  | |  | |  | |  | |  | |  | |  |  |  |
