# Supplementary material for: Combination of Hotspot Mutations With Methylation and Fragmentomic Profiles to Enhance Multi‐Cancer Early Detection
Source: Cancer Med. 2025 Jan 3;14(1):e70575. doi: 10.1002/cam4.70575 (PMC11695824; doi:10.1002/cam4.70575)
Supplement: Supplementary file 4 — Table S3. List of recurrent mutations (detected in at least 2 patients). [file CAM4-14-e70575-s001.docx]

| **Table S3: List of recurrent mutations (detected in at least 2 patients)** | | | | | |  | |  |
| --- | --- | --- | --- | --- | --- | --- | --- | --- |
|  |  |  |  |  |  | |  | |
| **SampleID** | **LABEL** | **Gene** | **AA mutation** | **CDS mutation** | **Variant_Classification** | |  | |
| ZMC004 | Colorectal cancer | KRAS | p.G12V | C>A | Single Nucleotide Variants (SNVs) | |  | |
| ZMC004 | Colorectal cancer | PIK3CA | p.R108H | G>A | Single Nucleotide Variants (SNVs) | |  | |
| ZMC005 | Colorectal cancer | APC | p.R232* | C>T | Single Nucleotide Variants (SNVs) | |  | |
| ZMC005 | Colorectal cancer | KRAS | p.G13D | C>T | Single Nucleotide Variants (SNVs) | |  | |
| ZMC006 | Colorectal cancer | KRAS | p.G12D | C>T | Single Nucleotide Variants (SNVs) | |  | |
| ZMC006 | Colorectal cancer | PIK3CA | p.E542K | G>A | Single Nucleotide Variants (SNVs) | |  | |
| ZMC008 | Colorectal cancer | KRAS | p.G12D | C>T | Single Nucleotide Variants (SNVs) | |  | |
| ZMC008 | Colorectal cancer | APC | p.R232* | C>T | Single Nucleotide Variants (SNVs) | |  | |
| ZMC009 | Colorectal cancer | KRAS | p.G12D | C>T | Single Nucleotide Variants (SNVs) | |  | |
| ZMC009 | Colorectal cancer | PIK3CA | p.E545K | G>A | Single Nucleotide Variants (SNVs) | |  | |
| ZMC040 | Colorectal cancer | TP53 | p.R248Q | C>T | Single Nucleotide Variants (SNVs) | |  | |
| ZMC056 | Colorectal cancer | TP53 | p.R306* | G>A | Single Nucleotide Variants (SNVs) | |  | |
| ZMC056 | Colorectal cancer | APC | p.R302* | C>T | Single Nucleotide Variants (SNVs) | |  | |
| ZMC065 | Colorectal cancer | AMER1 | p.R631* | G>A | Single Nucleotide Variants (SNVs) | |  | |
| ZMC075 | Colorectal cancer | KRAS | p.G13D | C>T | Single Nucleotide Variants (SNVs) | |  | |
| ZMC075 | Colorectal cancer | PIK3CA | p.H1047R | A>G | Single Nucleotide Variants (SNVs) | |  | |
| ZMC076 | Colorectal cancer | TP53 | p.L194R | A>C | Single Nucleotide Variants (SNVs) | |  | |
| ZMC119 | Colorectal cancer | TP53 | p.R213* | G>A | Single Nucleotide Variants (SNVs) | |  | |
| ZMC125 | Colorectal cancer | PIK3CA | p.H1047R | A>G | Single Nucleotide Variants (SNVs) | |  | |
| ZMC077 | Colorectal cancer | KRAS | p.G12C | C>A | Single Nucleotide Variants (SNVs) | |  | |
| ZMH004 | Liver cancer | KRAS | p.G12C | C>A | Single Nucleotide Variants (SNVs) | |  | |
| ZMH005 | Liver cancer | TERT | P.C228T | G>A | Single Nucleotide Variants (SNVs) | |  | |
| ZMH006 | Liver cancer | TERT | P.C228T | G>A | Single Nucleotide Variants (SNVs) | |  | |
| ZMH010 | Liver cancer | TP53 | p.C275F | C>A | Single Nucleotide Variants (SNVs) | |  | |
| ZMH011 | Liver cancer | TERT | P.C228T | G>A | Single Nucleotide Variants (SNVs) | |  | |
| ZMH012 | Liver cancer | TERT | P.C228T | G>A | Single Nucleotide Variants (SNVs) | |  | |
| ZMH015 | Liver cancer | KRAS | p.G13D | C>T | Single Nucleotide Variants (SNVs) | |  | |
| ZMH018 | Liver cancer | TERT | P.C228T | G>A | Single Nucleotide Variants (SNVs) | |  | |
| ZMH044 | Liver cancer | TERT | P.C228T | G>A | Single Nucleotide Variants (SNVs) | |  | |
| ZMH050 | Liver cancer | TP53 | p.C275F | C>A | Single Nucleotide Variants (SNVs) | |  | |
| ZMH052 | Liver cancer | TERT | P.C228T | G>A | Single Nucleotide Variants (SNVs) | |  | |
| ZMH053 | Liver cancer | TERT | P.C228T | G>A | Single Nucleotide Variants (SNVs) | |  | |
| ZMH059 | Liver cancer | TERT | P.C228T | G>A | Single Nucleotide Variants (SNVs) | |  | |
| ZMH060 | Liver cancer | TERT | P.C228T | G>A | Single Nucleotide Variants (SNVs) | |  | |
| ZMG019 | Gastric cancer | TP53 | p.R248Q | C>T | Single Nucleotide Variants (SNVs) | |  | |
| ZMG021 | Gastric cancer | PIK3CA | p.R108H | G>A | Single Nucleotide Variants (SNVs) | |  | |
| ZMG048 | Gastric cancer | PIK3CA | p.H1047R | A>G | Single Nucleotide Variants (SNVs) | |  | |
| ZMG131 | Gastric cancer | PIK3CA | p.H1047R | A>G | Single Nucleotide Variants (SNVs) | |  | |
| ZMG159 | Gastric cancer | PIK3CA | p.H1047R | A>G | Single Nucleotide Variants (SNVs) | |  | |
| ZMB040 | Breast cancer | PIK3CA | p.H1047R | A>G | Single Nucleotide Variants (SNVs) | |  | |
| ZMB146 | Breast cancer | PIK3CA | p.E545K | G>A | Single Nucleotide Variants (SNVs) | |  | |
| ZMB149 | Breast cancer | TP53 | p.R213* | G>A | Single Nucleotide Variants (SNVs) | |  | |
| ZMB185 | Breast cancer | PIK3CA | p.E542K | G>A | Single Nucleotide Variants (SNVs) | |  | |
| ZMB181 | Breast cancer | PIK3CA | p.E545K | G>A | Single Nucleotide Variants (SNVs) | |  | |
| ZMB183 | Breast cancer | PIK3CA | p.E542K | G>A | Single Nucleotide Variants (SNVs) | |  | |
| ZMB201 | Breast cancer | PIK3CA | p.H1047R | A>G | Single Nucleotide Variants (SNVs) | |  | |
| ZMB567 | Breast cancer | KRAS | p.G12D | C>T | Single Nucleotide Variants (SNVs) | |  | |
| LAAJ24 | Lung cancer | KRAS | p.G12C | C>A | Single Nucleotide Variants (SNVs) | |  | |
| LABD63 | Lung cancer | KRAS | p.G12V | C>A | Single Nucleotide Variants (SNVs) | |  | |
| LAAW21 | Lung cancer | EGFR | p.L858R | T>G | Single Nucleotide Variants (SNVs) | |  | |
| LABH01 | Lung cancer | KRAS | p.G12D | C>T | Single Nucleotide Variants (SNVs) | |  | |
| LHAF44 | Lung cancer | EGFR | E746_A750del | AGGAATTAAGAGAAGC>A | Deletion | |  | |
| L12866 | Lung cancer | EGFR | E746_A750del | AGGAATTAAGAGAAGC>A | Deletion | |  | |
| NL09 | Lung cancer | KRAS | p.G13C | C>A | Single Nucleotide Variants (SNVs) | |  | |
| LC019 | Colorectal cancer | TP53 | p.L194R | A>C | Single Nucleotide Variants (SNVs) | |  | |
| LC023 | Colorectal cancer | KRAS | p.G13D | C>T | Single Nucleotide Variants (SNVs) | |  | |
| LC034 | Colorectal cancer | APC | p.R302* | C>T | Single Nucleotide Variants (SNVs) | |  | |
| LC036 | Colorectal cancer | AMER1 | p.R631* | G>A | Single Nucleotide Variants (SNVs) | |  | |
| LC069 | Colorectal cancer | TP53 | p.R306* | G>A | Single Nucleotide Variants (SNVs) | |  | |
| LBG30 | Gastric cancer | TP53 | p.R306* | G>A | Single Nucleotide Variants (SNVs) | |  | |
| LC133 | Colorectal cancer | TP53 | R175H | C>T | Single Nucleotide Variants (SNVs) | |  | |
| LC135 | Colorectal cancer | KRAS | p.G12D | C>T | Single Nucleotide Variants (SNVs) | |  | |
| L12401 | Lung cancer | EGFR | E746_A750del | AGGAATTAAGAGAAGC>A | Deletion | |  | |
| L12406 | Lung cancer | KRAS | p.G12C | C>A | Single Nucleotide Variants (SNVs) | |  | |
| L12174 | Lung cancer | KRAS | p.G12C | C>A | Single Nucleotide Variants (SNVs) | |  | |
| L12997 | Lung cancer | EGFR | p.L858R | T>G | Single Nucleotide Variants (SNVs) | |  | |
| ZMC093 | Colorectal cancer | PIK3CA | p.E542K | G>A | Single Nucleotide Variants (SNVs) | |  | |
| ZMC094 | Colorectal cancer | APC | p.R232* | C>T | Single Nucleotide Variants (SNVs) | |  | |
| LABA02 | Lung cancer | TP53 | R175H | C>T | Single Nucleotide Variants (SNVs) | |  | |
| LAAN29 | Lung cancer | KRAS | p.G13C | C>A | Single Nucleotide Variants (SNVs) | |  | |
| LABH01 | Lung cancer | KRAS | p.G12D | C>T | Single Nucleotide Variants (SNVs) | |  | |
| LAAL31 | Lung cancer | KRAS | p.G12C | C>A | Single Nucleotide Variants (SNVs) | |  | |
| LAAL98 | Lung cancer | KRAS | p.G12V | C>A | Single Nucleotide Variants (SNVs) | |  | |
| LAAL98 | Lung cancer | TP53 | p.R213* | G>A | Single Nucleotide Variants (SNVs) | |  | |
| LAAM80 | Lung cancer | TP53 | p.R248Q | C>T | Single Nucleotide Variants (SNVs) | |  | |
| LAAM90 | Lung cancer | KRAS | p.G12C | C>A | Single Nucleotide Variants (SNVs) | |  | |
| LAAM12 | Lung cancer | EGFR | E746_A750del | AGGAATTAAGAGAAGC>A | Deletion | |  | |
