# Supplementary material for: Combination of Hotspot Mutations With Methylation and Fragmentomic Profiles to Enhance Multi‐Cancer Early Detection
Source: Cancer Med. 2025 Jan 3;14(1):e70575. doi: 10.1002/cam4.70575 (PMC11695824; doi:10.1002/cam4.70575)
Supplement: Supplementary file 5 — Table S4. Mutation profiling and SPOT‐MAS model score of 255 cancer patients. [file CAM4-14-e70575-s005.docx]

| **Table S4: Mutation profiling and SPOT-MAS model score of 255 cancer patients** | | | | | | | |  | | |
| --- | --- | --- | --- | --- | --- | --- | --- | --- | --- | --- |
| **SampleID** | **Type** | **SPOT-MAS model score cutoff = 0.60368985** | **SPOT-MAS model detection status** | **VAF cutoff=0.05%** | **Hotspot Detection status** | **Hotspot mutation** | **Stage** | | **Actionable mutation FDA approved drugs and NCCN 2024 recommended)** |  |
| ZMC001 | Colorectal cancer | 1.00 | Detected | 3.87 | Detected | TP53_V173L | II | | No |  |
| ZMC002 | Colorectal cancer | 0.91 | Detected | <0.05% | Not detected | Negative | II | | No |  |
| ZMC004 | Colorectal cancer | 0.73 | Detected | 1.554495103 | Detected | TP53_F270V  APC_Q1469*  NTRK3_R459Q  KRAS_G12V  PIK3CA_R108H | III | | Yes |  |
| ZMC005 | Colorectal cancer | 0.72 | Detected | 0.908658212 | Detected | ERBB3_D297Y  TP53_T253A  APC_R232*  KRAS_G13D  PIK3CA_Q546K  PIK3R1_R348* | II | | Yes |  |
| ZMC006 | Colorectal cancer | 0.23 | Not detected | 0.124250229 | Detected | APC_S1327*  KRAS_G12D  PIK3CA_E542K  TCF7L2_D10Efs*2 | II | | Yes |  |
| ZMC008 | Colorectal cancer | 0.88 | Detected | 0.23 | Detected | KRAS_G12D  APC_R232* | II | | Yes |  |
| ZMC009 | Colorectal cancer | 0.93 | Detected | 0.3 | Detected | KRAS_G12D  PIK3CA_E545K | III | | Yes |  |
| ZMC016 | Colorectal cancer | 0.95 | Detected | <0.05% | Not detected | Negative | I | | No |  |
| ZMC029 | Colorectal cancer | 1.00 | Detected | 3.41 | Detected | APC_Q1338* | II | | No |  |
| ZMC037 | Colorectal cancer | 0.93 | Detected | 0.07 | Detected | TP53_E285* | III | | No |  |
| ZMC040 | Colorectal cancer | 0.95 | Detected | 0.4 | Detected | KRAS_Q61K  TP53_R248Q | III | | Yes |  |
| ZMC053 | Colorectal cancer | 0.98 | Detected | 0.08 | Detected | APC_E1397* | II | | No |  |
| ZMC056 | Colorectal cancer | 0.99 | Detected | 0.866666667 | Detected | TP53_R306*  APC_R302*  FBXW7_S582L | II | | No |  |
| ZMC060 | Colorectal cancer | 1.00 | Detected | <0.05% | Not detected | Negative | III | | No |  |
| ZMC065 | Colorectal cancer | 1.00 | Detected | 0.06 | Detected | AMER1_R631* | I | | No |  |
| ZMC069 | Colorectal cancer | 0.99 | Detected | 0.22 | Detected | TP53_R196* | III | | No |  |
| ZMC074 | Colorectal cancer | 0.28 | Not detected | <0.05% | Not detected | Negative | I | | No |  |
| ZMC075 | Colorectal cancer | 0.33 | Not detected | 0.15 | Detected | KRAS_G13D  KMT2C_W1639*  PIK3CA_H1047R  KMT2C_W1639* | III | | Yes |  |
| ZMC076 | Colorectal cancer | 0.09 | Not detected | 0.14 | Detected | TP53_L194R | II | | No |  |
| ZMC119 | Colorectal cancer | 0.18 | Not detected | 0.09 | Detected | TP53_L252_I254del  TP53_L252_I254del  KRAS_Q61H  APC_R213* | II | | Yes |  |
| ZMC124 | Colorectal cancer | 0.24 | Not detected | 0.23 | Detected | KDM6A_R1307W | Nonmetastatic with unknow staging | | No |  |
| ZMC082 | Colorectal cancer | 0.13 | Not detected | <0.05% | Not detected | Negative | II | | No |  |
| ZMC125 | Colorectal cancer | 0.78 | Detected | 0.3 | Detected | TP53_R290Afs*55  TP53_R290Afs*55  PIK3CA_H1047R | III | | No |  |
| YCAA33 | Colorectal cancer | 0.91 | Detected | <0.05% | Not detected | Negative | Nonmetastatic with unknow staging | | No |  |
| YCAA35 | Colorectal cancer | 0.99 | Detected | <0.05% | Not detected | Negative | Nonmetastatic with unknow staging | | No |  |
| YCAB60 | Colorectal cancer | 0.12 | Not detected | <0.05% | Not detected | Negative | Nonmetastatic with unknow staging | | No |  |
| ZMC072 | Colorectal cancer | 0.85 | Detected | 0.18 | Detected | CTNNB1_T41A | II | | No |  |
| ZMC077 | Colorectal cancer | 0.38 | Not detected | 0.11 | Detected | KRAS_G12C  TP53_Y234H  TP53_Y234H | III | | Yes |  |
| ZMC123 | Colorectal cancer | 0.11 | Not detected | 0.1 | Detected | TP53_S215N | II | | No |  |
| ZMH002 | Liver cancer | 1.00 | Detected | 2.0225 | Detected | LRP1B_W2657*  NOTCH2_E1216*  FAT1_C4022Y  PDE4DIP_V912L | III | | No |  |
| ZMH004 | Liver cancer | 1.00 | Detected | 17.16457285 | Detected | KRAS_G12C  AXIN1_P848R | III | | No |  |
| ZMH005 | Liver cancer | 0.99 | Detected | 5.21 | Detected | ARID1A_R2232Gfs*35  TERT_P.C228T  AXIN1_P26Lfs*58 | I | | No |  |
| ZMH006 | Liver cancer | 1.00 | Detected | 31.54023292 | Detected | TERT_P.C228T  TP53_N268_F270del  TSC2_F1501Sfs*21 | II | | No |  |
| ZMH008 | Liver cancer | 1.00 | Detected | 2.675 | Detected | CTNNB1_S33F  TP53_V143E | III | | No |  |
| ZMH010 | Liver cancer | 0.91 | Detected | 2.026666667 | Detected | TP53_C275F  ERBB4_L566l  NOTCH1_C1018* | II | | No |  |
| ZMH011 | Liver cancer | 1.00 | Detected | 5.51 | Detected | TP53_I332N  TERT_P.C228T | III | | No |  |
| ZMH012 | Liver cancer | 0.98 | Detected | 2.08 | Detected | TP53_R209*  TERT_P.C228T | III | | No |  |
| ZMH014 | Liver cancer | 0.88 | Detected | <0.05% | Not detected | Negative | I | | No |  |
| ZMH015 | Liver cancer | 0.85 | Detected | 0.468 | Detected | KRAS_G13D  CTNNB1_T34I  PTPRT_R1349H  PTPRT_Y280D  PTPRT_Y280D | I | | No |  |
| ZMH018 | Liver cancer | 1.00 | Detected | 17.374 | Detected | KRAS_A59G  AXIN1_W444*  ATM_D2016H  TERT_P.C228T  AXIN1_W444* | II | | No |  |
| ZMH019 | Liver cancer | 0.82 | Detected | 0.11 | Detected | CTNNB1_S45P | I | | No |  |
| ZMH023 | Liver cancer | 1.00 | Detected | 2.53 | Detected | NCOR1_R17147L  FOXP1_M137I | II | | No |  |
| ZMH025 | Liver cancer | 0.92 | Detected | 1.51 | Detected | TP53_R249S | I | | No |  |
| ZMH032 | Liver cancer | 0.97 | Detected | 0.05 | Detected | ALK_S866L | III | | No |  |
| ZMH034 | Liver cancer | 1.00 | Detected | 5.48 | Detected | CTNNB1_S45F | I | | No |  |
| ZMH036 | Liver cancer | 1.00 | Detected | 7.54 | Detected | ARID1A_A2235Rfs*30 | II | | No |  |
| ZMH039 | Liver cancer | 1.00 | Detected | 4.596666667 | Detected | TP53_R213L  CTNNB1_S33P  CTNNB1_G34V | II | | No |  |
| ZMH043 | Liver cancer | 0.95 | Detected | 0.48 | Detected | CTNNB1_S45Y  ARID1A_K1938*  ARID1A_K1938* | II | | No |  |
| ZMH044 | Liver cancer | 1.00 | Detected | 0.99 | Detected | TP53_Y236C  TERT_P.C228T | I | | No |  |
| ZMH048 | Liver cancer | 1.00 | Detected | 10.47 | Detected | CTNNB1_D32G  NCOR2_A1069S | II | | No |  |
| ZMH050 | Liver cancer | 1.00 | Detected | 30.28 | Detected | TP53_C275F | III | | No |  |
| ZMH052 | Liver cancer | 0.99 | Detected | 1.64 | Detected | AXIN1_W247*  TERT_P.C228T | I | | No |  |
| ZMH053 | Liver cancer | 1.00 | Detected | 2.47 | Detected | TERT_P.C228T | I | | No |  |
| ZMH054 | Liver cancer | 1.00 | Detected | 7.55 | Detected | ARID1A_F1999* | II | | No |  |
| ZMH059 | Liver cancer | 1.00 | Detected | 8.486666667 | Detected | TP53_H179R  TERT_P.C228T  ARID2_K569* | II | | No |  |
| ZMH060 | Liver cancer | 1.00 | Detected | 26.29 | Detected | TP53_R280_R283delinsS  AXIN1_K379*  ARID2_T879A  TERT_P.C228T | II | | No |  |
| ZMH062 | Liver cancer | 1.00 | Detected | 14.41 | Detected | TP53_Y205C | III | | No |  |
| ZMG086 | Gastric cancer | 0.10 | Not detected | 0.13 | Detected | NCOR1_S172L | Nonmetastatic with unknow staging | | No |  |
| ZMG005 | Gastric cancer | 0.63 | Detected | <0.05% | Not detected | Negative | III | | No |  |
| ZMG010 | Gastric cancer | 1.00 | Detected | <0.05% | Not detected | Negative | Nonmetastatic with unknow staging | | No |  |
| ZMG011 | Gastric cancer | 1.00 | Detected | 0.16 | Detected | SMARCA4_T910M  FAT4_A2178T  ESR1_E247K  CREBBP_R672C  ETV1_R440H  SMARCB1_R377H | II | | No |  |
| ZMG013 | Gastric cancer | 0.98 | Detected | 0.06 | Detected | ERBB2_R678Q | II | | Yes |  |
| ZMG019 | Gastric cancer | 1.00 | Detected | 0.14 | Detected | TP53_R248Q | III | | No |  |
| ZMG021 | Gastric cancer | 0.99 | Detected | 0.06 | Detected | TP53_R248W  PIK3CA_R108H  BRCA2_D707N | Nonmetastatic with unknow staging | | No |  |
| ZMG024 | Gastric cancer | 0.98 | Detected | <0.05% | Not detected | Negative | I | | No |  |
| ZMG027 | Gastric cancer | 1.00 | Detected | 0.09 | Detected | TP53_H179Y  PREX2_I1527T | III | | No |  |
| ZMG028 | Gastric cancer | 0.21 | Not detected | 0.23 | Detected | CDH1_I615Sfs*46  TP53_Y126C | III | | No |  |
| ZMG029 | Gastric cancer | 0.34 | Not detected | <0.05% | Not detected | Negative | II | | No |  |
| ZMG032 | Gastric cancer | 1.00 | Detected | 0.05 | Detected | TP53_V173M | II | | No |  |
| ZMG038 | Gastric cancer | 0.79 | Detected | <0.05% | Not detected | Negative | II | | No |  |
| ZMG042 | Gastric cancer | 0.89 | Detected | 0.05 | Detected | CDH1_W156G | II | | No |  |
| ZMG045 | Gastric cancer | 0.98 | Detected | <0.05% | Not detected | Negative | III | | No |  |
| ZMG046 | Gastric cancer | 0.96 | Detected | <0.05% | Not detected | Negative | III | | No |  |
| ZMG047 | Gastric cancer | 0.99 | Detected | 0.09 | Detected | TP53_C176F  ARID1A_R2158*  PIK3CA_D138N | II | | No |  |
| ZMG048 | Gastric cancer | 0.90 | Detected | 1.71 | Detected | PIK3CA_H1047R  TP53_R273H | II | | No |  |
| ZMG049 | Gastric cancer | 0.97 | Detected | 1.04 | Detected | TP53_C277G  BCOR_G1359E  TSHR_L426Q | Nonmetastatic with unknow staging | | No |  |
| ZMG054 | Gastric cancer | 0.98 | Detected | 0.15 | Detected | TP53_C275G  PIK3CA_L602P  LRP1B_D4407Y  PTPRS_R1432H  PARP1_L617F  DNMT3B_R410Q | III | | No |  |
| ZMG061 | Gastric cancer | 0.93 | Detected | <0.05% | Not detected | Negative | Nonmetastatic with unknow staging | | No |  |
| ZMG062 | Gastric cancer | 0.98 | Detected | 0.47 | Detected | TP53_E171*  ERBB2_I767M  ARID1A_D1913N | Nonmetastatic with unknow staging | | Yes |  |
| ZMG069 | Gastric cancer | 0.42 | Not detected | <0.05% | Not detected | Negative | III | | No |  |
| ZMG076 | Gastric cancer | 0.20 | Not detected | <0.05% | Not detected | Negative | Nonmetastatic with unknow staging | | No |  |
| ZMG088 | Gastric cancer | 0.55 | Not detected | 0.25 | Detected | CDH1_E223G | III | | No |  |
| ZMG097 | Gastric cancer | 0.40 | Not detected | <0.05% | Not detected | Negative | II | | No |  |
| ZMG099 | Gastric cancer | 0.38 | Not detected | <0.05% | Not detected | Negative | III | | No |  |
| ZMG115 | Gastric cancer | 0.92 | Detected | <0.05% | Not detected | Negative | III | | No |  |
| ZMG117 | Gastric cancer | 0.18 | Not detected | <0.05% | Not detected | Negative | Nonmetastatic with unknow staging | | No |  |
| ZMG119 | Gastric cancer | 0.99 | Detected | <0.05% | Not detected | Negative | II | | No |  |
| ZMG123 | Gastric cancer | 0.30 | Not detected | <0.05% | Not detected | Negative | I | | No |  |
| ZMG124 | Gastric cancer | 0.37 | Not detected | <0.05% | Not detected | Negative | I | | No |  |
| ZMG126 | Gastric cancer | 0.44 | Not detected | <0.05% | Not detected | Negative | III | | No |  |
| ZMG127 | Gastric cancer | 0.95 | Detected | 0.11 | Detected | TP53_Q136* | II | | No |  |
| ZMG089 | Gastric cancer | 0.30 | Not detected | <0.05% | Not detected | Negative | Nonmetastatic with unknow staging | | No |  |
| ZMG091 | Gastric cancer | 0.12 | Not detected | <0.05% | Not detected | Negative | III | | No |  |
| ZMG131 | Gastric cancer | 0.40 | Not detected | 0.41 | Detected | PIK3CA_H1047R  CTNNB1_R151C  TP53_R267W  TAP1_L494*  ARID1A_F2141Sfs*59 | III | | No |  |
| ZMG132 | Gastric cancer | 0.67 | Detected | 0.11 | Detected | SMARCA4_L180V  DOT1L_V1510M | II | | No |  |
| ZMG129 | Gastric cancer | 0.47 | Not detected | <0.05% | Not detected | Negative | I | | No |  |
| ZMG142 | Gastric cancer | 0.98 | Detected | 0.49 | Detected | ERBB3_A245V  KMT2C_R2066* | II | | No |  |
| ZMG146 | Gastric cancer | 0.66 | Detected | <0.05% | Not detected | Negative | Nonmetastatic with unknow staging | | No |  |
| ZMG149 | Gastric cancer | 0.10 | Not detected | <0.05% | Not detected | Negative | III | | No |  |
| ZMG151 | Gastric cancer | 0.36 | Not detected | 0.5 | Detected | CDH1_Q23*  RHOA_V33M  PARP1_T124A  DOT1L_A1230V  CDH1_L214P | Nonmetastatic with unknow staging | | No |  |
| ZMG154 | Gastric cancer | 0.10 | Not detected | 0.18 | Detected | TP53_673-2A>T | III | | No |  |
| ZMG156 | Gastric cancer | 0.98 | Detected | 5.64 | Detected | LRP1B_N1179Y  APC_D1015Vfs*7  PIK3CD_R663C | III | | No |  |
| ZMG157 | Gastric cancer | 0.73 | Detected | <0.05% | Not detected | Negative | III | | No |  |
| ZMG159 | Gastric cancer | 0.88 | Detected | 6.41 | Detected | TP53_R273H  PIK3CA_H1047R  ELF3_F11Lfs*32  STAT5B_X592_splice  RAB35_A151T  RNF43_S268Cfs*11 | III | | No |  |
| ZMG160 | Gastric cancer | 0.89 | Detected | <0.05% | Not detected | Negative | III | | No |  |
| ZMB501 | Breast cancer | 0.73 | Detected | <0.05% | Not detected | Negative | II | | No |  |
| ZMB526 | Breast cancer | 0.93 | Detected | <0.05% | Not detected | Negative | II | | No |  |
| ZMB028 | Breast cancer | 0.05 | Not detected | <0.05% | Not detected | Negative | II | | No |  |
| ZMB040 | Breast cancer | 0.26 | Not detected | 0.88 | Detected | PIK3CA_H1047R  RB1_2489+1G>A  NRAS_Q61K  GRIN2A_V1018M | II | | Yes |  |
| ZMB134 | Breast cancer | 0.98 | Detected | <0.05% | Not detected | Negative | III | | No |  |
| ZMB135 | Breast cancer | 0.89 | Detected | <0.05% | Not detected | Negative | II | | No |  |
| ZMB138 | Breast cancer | 0.78 | Detected | 1 read | Detected | BRCA2_S1720Ffs*7 | II | | Yes |  |
| ZMB139 | Breast cancer | 0.27 | Not detected | <0.05% | Not detected | Negative | II | | No |  |
| ZMB145 | Breast cancer | 0.29 | Not detected | <0.05% | Not detected | Negative | Nonmetastatic with unknow staging | | No |  |
| ZMB146 | Breast cancer | 0.09 | Not detected | 0.27 | Detected | PIK3CA_E545K | III | | Yes |  |
| ZMB148 | Breast cancer | 0.30 | Not detected | 0.08 | Detected | PIK3R1_c.836+1dup | I | | No |  |
| ZMB149 | Breast cancer | 0.14 | Not detected | 0.16 | Detected | TP53_R213* | Nonmetastatic with unknow staging | | No |  |
| ZMB152 | Breast cancer | 0.31 | Not detected | <0.05% | Not detected | Negative | II | | No |  |
| ZMB153 | Breast cancer | 0.81 | Detected | 1.16 | Detected | RB1_S215* | Nonmetastatic with unknow staging | | No |  |
| ZMB154 | Breast cancer | 0.26 | Not detected | <0.05% | Not detected | Negative | II | | No |  |
| ZMB168 | Breast cancer | 0.21 | Not detected | <0.05% | Not detected | Negative | I | | No |  |
| ZMB170 | Breast cancer | 0.05 | Not detected | 0.08 | Detected | RNF43_S446F | II | | No |  |
| ZMB171 | Breast cancer | 0.26 | Not detected | <0.05% | Not detected | Negative | I | | No |  |
| ZMB178 | Breast cancer | 0.28 | Not detected | <0.05% | Not detected | Negative | I | | No |  |
| ZMB185 | Breast cancer | 0.18 | Not detected | 0.46 | Detected | PIK3CA_E542K | III | | Yes |  |
| ZMB210 | Breast cancer | 0.21 | Not detected | 0.24 | Detected | TP53_C229Yfs*10 | III | | No |  |
| ZMB510 | Breast cancer | 0.82 | Detected | <0.05% | Not detected | Negative | II | | No |  |
| ZMB512 | Breast cancer | 0.88 | Detected | 0.13 | Detected | TP53_F341Lfs*2 | I | | No |  |
| ZMB522 | Breast cancer | 0.63 | Detected | 0.05 | Detected | MAP3K1_L1360F | I | | No |  |
| ZMB524 | Breast cancer | 0.85 | Detected | - | Detected | IGFR1_E1162G  SPEN_A3662P  PTEN_G36*  TP53_P278R | II | | Yes |  |
| ZMB527 | Breast cancer | 0.74 | Detected | <0.05% | Not detected | Negative | I | | No |  |
| ZMB528 | Breast cancer | 0.32 | Not detected | <0.05% | Not detected | Negative | II | | No |  |
| ZMB142 | Breast cancer | 0.39 | Not detected | <0.05% | Not detected | Negative | II | | No |  |
| ZMB163 | Breast cancer | 0.95 | Detected | 1.15 | Detected | GATA3_D336Gfs*17 | I | | No |  |
| ZMB167 | Breast cancer | 0.32 | Not detected | <0.05% | Not detected | Negative | Nonmetastatic with unknow staging | | No |  |
| ZMB181 | Breast cancer | 0.75 | Detected | 0.12 | Detected | PIK3CA_E545K | II | | Yes |  |
| ZMB183 | Breast cancer | 0.17 | Not detected | 0.37 | Detected | PIK3CA_E542K | II | | Yes |  |
| ZMB201 | Breast cancer | 0.80 | Detected | 0.06 | Detected | PIK3CA_H1047R | II | | Yes |  |
| ZMB536 | Breast cancer | 0.10 | Not detected | <0.05% | Not detected | Negative | II | | No |  |
| ZMB537 | Breast cancer | 0.27 | Not detected | 0.17 | Detected | FOXA1_H247Q | III | | No |  |
| ZMB538 | Breast cancer | 0.13 | Not detected | <0.05% | Not detected | Negative | II | | No |  |
| ZMB540 | Breast cancer | 0.06 | Not detected | <0.05% | Not detected | Negative | II | | No |  |
| ZMB544 | Breast cancer | 0.11 | Not detected | <0.05% | Not detected | Negative | II | | No |  |
| ZMB548 | Breast cancer | 0.04 | Not detected | <0.05% | Not detected | Negative | I | | No |  |
| ZMB549 | Breast cancer | 0.90 | Detected | <0.05% | Not detected | Negative | II | | No |  |
| ZMB551 | Breast cancer | 0.64 | Detected | <0.05% | Not detected | Negative | I | | No |  |
| ZMB557 | Breast cancer | 0.64 | Detected | <0.05% | Not detected | Negative | II | | No |  |
| ZMB559 | Breast cancer | 0.80 | Detected | <0.05% | Not detected | Negative | II | | No |  |
| ZMB560 | Breast cancer | 0.39 | Not detected | <0.05% | Not detected | Negative | II | | No |  |
| ZMB561 | Breast cancer | 0.87 | Detected | <0.05% | Not detected | Negative | III | | No |  |
| ZMB567 | Breast cancer | 0.86 | Detected | 0.32 | Detected | PTEN_T319*  TP53_N239D  KMT2C_E633Kfs*2  PIK3CA_P104del  KRAS_G12D | II | | Yes |  |
| ZMB569 | Breast cancer | 0.96 | Detected | <0.05% | Not detected | Negative | II | | No |  |
| ZMB570 | Breast cancer | 0.98 | Detected | <0.05% | Not detected | Negative | II | | No |  |
| ZMB572 | Breast cancer | 0.90 | Detected | <0.05% | Not detected | Negative | II | | No |  |
| ZMB574 | Breast cancer | 0.93 | Detected | <0.05% | Not detected | Negative | II | | No |  |
| ZMB575 | Breast cancer | 0.72 | Detected | 0.11 | Detected | KRAS_G12S | II | | No |  |
| ZMB576 | Breast cancer | 0.96 | Detected | <0.05% | Not detected | Negative | II | | No |  |
| ZMB584 | Breast cancer | 0.33 | Not detected | <0.05% | Not detected | Negative | II | | No |  |
| ZMB585 | Breast cancer | 0.97 | Detected | <0.05% | Not detected | Negative | II | | No |  |
| ZMB590 | Breast cancer | 0.80 | Detected | <0.05% | Not detected | Negative | II | | No |  |
| ZMB596 | Breast cancer | 0.81 | Detected | <0.05% | Not detected | Negative | II | | No |  |
| ZMB598 | Breast cancer | 0.96 | Detected | <0.05% | Not detected | Negative | II | | No |  |
| ZMB601 | Breast cancer | 0.26 | Not detected | 0.82 | Detected | TP53_P151R | II | | No |  |
| ZMB605 | Breast cancer | 0.11 | Not detected | <0.05% | Not detected | Negative | II | | No |  |
| ZMB607 | Breast cancer | 0.97 | Detected | <0.05% | Not detected | Negative | II | | No |  |
| ZMB608 | Breast cancer | 0.91 | Detected | <0.05% | Not detected | Negative | II | | No |  |
| ZMB609 | Breast cancer | 0.05 | Not detected | <0.05% | Not detected | Negative | II | | No |  |
| ZMB612 | Breast cancer | 0.98 | Detected | <0.05% | Not detected | Negative | II | | No |  |
| ZMB007 | Breast cancer | 0.25 | Not detected | <0.05% | Not detected | Negative | II | | No |  |
| ZMG164 | Gastric cancer | 0.04 | Not detected | 0.06 | Detected | RNF43_R117Afs*41  B2M_L15Ffs*41  PARP1_R841H  GNAS_R356C | II | | No |  |
| ZMG031 | Gastric cancer | 0.98 | Detected | 0.35 | Detected | ALK_R1209Q | III | | No |  |
| ZMG150 | Gastric cancer | 0.86 | Detected | <0.05% | Not detected | Negative | III | | No |  |
| ZMH027 | Liver cancer | 1.00 | Detected | 9.78 | Detected | TP53_G334V | II | | No |  |
| LAAF62 | Lung cancer | 0.91 | Detected | <0.05% | Not detected | Negative | III | | No |  |
| LAAJ24 | Lung cancer | 0.92 | Detected | 0.21 | Detected | STK11_E223*  KRAS_G12C | III | | Yes |  |
| LABF82 | Lung cancer | 0.99 | Detected | <0.05% | Not detected | Negative | III | | No |  |
| LABB48 | Lung cancer | 0.74 | Detected | 0.31 | Detected | EGFR_L747_E749del | III | | Yes |  |
| LABD46 | Lung cancer | 0.99 | Detected | 1.78 | Detected | KRAS_G12A | III | | Yes |  |
| LABD63 | Lung cancer | 1.00 | Detected | 8.51 | Detected | KRAS_G12V | III | | Yes |  |
| LAAW21 | Lung cancer | 1.00 | Detected | 37.83 | Detected | EGFR_L858R | III | | Yes |  |
| YCAB67 | Lung cancer | 0.97 | Detected | <0.05% | Not detected | Negative | Nonmetastatic with unknow staging | | No |  |
| LABH01 | Lung cancer | 1.00 | Detected | 1.05 | Detected | KRAS_G12D | III | | Yes |  |
| LHAF44 | Lung cancer | 0.63 | Detected | 0.06 | Detected | EGFR_E746_A750del | III | | Yes |  |
| YHAA03 | Lung cancer | 0.20 | Not detected | <0.05% | Not detected | Negative | Nonmetastatic with unknow staging | | No |  |
| YHAA05 | Lung cancer | 0.81 | Detected | <0.05% | Not detected | Negative | Nonmetastatic with unknow staging | | No |  |
| L12866 | Lung cancer | 1.00 | Detected | 16.31 | Detected | APC_G309*  KEAP1_E41*  TP53_V218Gfs*  STK11_E70Dfs*  CAMTA1_E540* | III | | Yes |  |
| L12964 | Lung cancer | 0.75 | Detected | 24.68 | Detected | ATM_R337C  EGFR_H773dup | III | | Yes |  |
| NL09 | Lung cancer | 0.05 | Not detected | 0.22 | Detected | KRAS_G13C  TP53_G117V | Nonmetastatic with unknow staging | | Yes |  |
| NL11 | Lung cancer | 0.15 | Not detected | <0.05% | Not detected | Negative | Nonmetastatic with unknow staging | | No |  |
| NL27 | Lung cancer | 0.76 | Detected | <0.05% | Not detected | Negative | Nonmetastatic with unknow staging | | No |  |
| LABA97 | Lung cancer | 0.24 | Not detected | <0.05% | Not detected | Negative | III | | No |  |
| QHAA87 | Lung cancer | 0.97 | Detected | <0.05% | Not detected | Negative | III | | No |  |
| LBG073 | Gastric cancer | 0.89 | Detected | <0.05% | Not detected | Negative | Nonmetastatic with unknow staging | | No |  |
| LBG66 | Gastric cancer | 0.70 | Detected | <0.05% | Not detected | Negative | II | | No |  |
| LBG32 | Gastric cancer | 0.83 | Detected | <0.05% | Not detected | Negative | I | | No |  |
| LBG38 | Gastric cancer | 0.15 | Not detected | <0.05% | Not detected | Negative | I | | No |  |
| LBG42 | Gastric cancer | 1.00 | Detected | <0.05% | Not detected | Negative | I | | No |  |
| LBG43 | Gastric cancer | 0.16 | Not detected | <0.05% | Not detected | Negative | I | | No |  |
| LBG44 | Gastric cancer | 1.00 | Detected | <0.05% | Not detected | Negative | I | | No |  |
| LBG45 | Gastric cancer | 0.49 | Not detected | <0.05% | Not detected | Negative | I | | No |  |
| ZMG111 | Gastric cancer | 0.83 | Detected | <0.05% | Not detected | Negative | II | | No |  |
| LBG67 | Gastric cancer | 0.79 | Detected | <0.05% | Not detected | Negative | I | | No |  |
| LC002 | Colorectal cancer | 0.32 | Not detected | <0.05% | Not detected | Negative | II | | No |  |
| LC007 | Colorectal cancer | 0.75 | Detected | <0.05% | Not detected | Negative | II | | No |  |
| LC010 | Colorectal cancer | 0.38 | Not detected | <0.05% | Not detected | Negative | II | | No |  |
| LC015 | Colorectal cancer | 0.84 | Detected | <0.05% | Not detected | Negative | Nonmetastatic with unknow staging | | No |  |
| LC018 | Colorectal cancer | 0.25 | Not detected | 0.12 | Detected | FBXW7_R479Q | II | | No |  |
| LC019 | Colorectal cancer | 0.26 | Not detected | 0.155 | Detected | BRAF_V600E  TP53_L194R | Nonmetastatic with unknow staging | | Yes |  |
| LC021 | Colorectal cancer | 0.16 | Not detected | 1.06 | Detected | TP53_R175C | Nonmetastatic with unknow staging | | No |  |
| LC023 | Colorectal cancer | 0.95 | Detected | 0.71 | Detected | KRAS_G13D | Nonmetastatic with unknow staging | | Yes |  |
| LC029 | Colorectal cancer | 0.72 | Detected | <0.05% | Not detected | Negative | Nonmetastatic with unknow staging | | No |  |
| LC034 | Colorectal cancer | 0.31 | Not detected | 0.2 | Detected | APC_R302*  APC_P1319Lfs*2 | Nonmetastatic with unknow staging | | No |  |
| LC036 | Colorectal cancer | 0.84 | Detected | 0.11 | Detected | AMER1_R631* | Nonmetastatic with unknow staging | | No |  |
| LC038 | Colorectal cancer | 0.79 | Detected | <0.05% | Not detected | Negative | Nonmetastatic with unknow staging | | No |  |
| LC043 | Colorectal cancer | 0.72 | Detected | <0.05% | Not detected | Negative | Nonmetastatic with unknow staging | | No |  |
| LC046 | Colorectal cancer | 0.91 | Detected | <0.05% | Not detected | Negative | Nonmetastatic with unknow staging | | No |  |
| LC049 | Colorectal cancer | 0.20 | Not detected | <0.05% | Not detected | Negative | Nonmetastatic with unknow staging | | No |  |
| LC069 | Colorectal cancer | 0.19 | Not detected | 0.59 | Detected | TP53_R306* | I | | No |  |
| LC084 | Colorectal cancer | 0.83 | Detected | <0.05% | Not detected | Negative | Nonmetastatic with unknow staging | | No |  |
| LC091 | Colorectal cancer | 0.89 | Detected | 7.336666667 | Detected | TP53_G245A | Nonmetastatic with unknow staging | | No |  |
| LC053 | Colorectal cancer | 0.14 | Not detected | <0.05% | Not detected | Negative | Nonmetastatic with unknow staging | | No |  |
| LBG30 | Gastric cancer | 1.00 | Detected | 0.12 | Detected | TP53_R306* | I | | No |  |
| LC131 | Colorectal cancer | 0.17 | Not detected | <0.05% | Not detected | Negative | III | | No |  |
| LC133 | Colorectal cancer | 0.88 | Detected | 0.63 | Detected | TP53_R175H | III | | No |  |
| LC135 | Colorectal cancer | 0.31 | Not detected | 1.57 | Detected | KRAS_G12D | II | | Yes |  |
| LC136 | Colorectal cancer | 0.96 | Detected | <0.05% | Not detected | Negative | Nonmetastatic with unknow staging | | No |  |
| LC139 | Colorectal cancer | 0.64 | Detected | 0.17 | Detected | TP53_Y220C  APC_R1399Ffs*9 | III | | No |  |
| LC143 | Colorectal cancer | 0.06 | Not detected | <0.05% | Not detected | Negative | Nonmetastatic with unknow staging | | No |  |
| LABY23 | Colorectal cancer | 0.04 | Not detected | <0.05% | Not detected | Negative | III | | No |  |
| LABY25 | Colorectal cancer | 0.25 | Not detected | <0.05% | Not detected | Negative | II | | No |  |
| L12401 | Lung cancer | 0.99 | Detected | 0.05 | Detected | EGFR_E746_A750del | III | | Yes |  |
| L11979 | Lung cancer | 0.34 | Not detected | <0.05% | Not detected | Negative | III | | No |  |
| L12406 | Lung cancer | 0.99 | Detected | 3.98 | Detected | KRAS_G12C | III | | Yes |  |
| L12174 | Lung cancer | 1.00 | Detected | 11.11 | Detected | KRAS_G12C  TP53_K120* | I | | Yes |  |
| L12859 | Lung cancer | 0.20 | Not detected | <0.05% | Not detected | Negative | II | | No |  |
| L12880 | Lung cancer | 0.28 | Not detected | <0.05% | Not detected | Negative | III | | No |  |
| L12997 | Lung cancer | 0.82 | Detected | 4.075 | Detected | EGFR_L858R  EGFR_T790M | III | | Yes |  |
| L13200 | Lung cancer | 0.10 | Not detected | <0.05% | Not detected | Negative | III | | No |  |
| LAAD30 | Lung cancer | 0.87 | Detected | <0.05% | Not detected | Negative | III | | No |  |
| ZMC093 | Colorectal cancer | 0.17 | Not detected | 0.12 | Detected | PIK3CA_E542K  GNAS_R201C  SMARCA4_G1232S  NRAS_A146V | Nonmetastatic with unknow staging | | Yes |  |
| ZMC094 | Colorectal cancer | 0.74 | Detected | 0.09 | Detected | APC_R232*  TP53_L93Vfs*55 | Nonmetastatic with unknow staging | | No |  |
| ZMC211 | Colorectal cancer | 0.76 | Detected | 0.14 | Detected | TP53_C275Y  APC_E1309Dfs*4 | Nonmetastatic with unknow staging | | No |  |
| LABA02 | Lung cancer | 0.98 | Detected | 4.95 | Detected | TP53_R175HC>T | Nonmetastatic with unknow staging | | No |  |
| LABD60 | Lung cancer | 0.95 | Detected | <0.05% | Not detected | Negative | Nonmetastatic with unknow staging | | No |  |
| LAAZ94 | Lung cancer | 0.78 | Detected | <0.05% | Not detected | Negative | Nonmetastatic with unknow staging | | No |  |
| LAAN29 | Lung cancer | 0.96 | Detected | 0.345 | Detected | KRAS_Q22RT>C  KRAS_G13CC>A | Nonmetastatic with unknow staging | | Yes |  |
| LAAT20 | Lung cancer | 0.99 | Detected | <0.05% | Not detected | Negative | III | | No |  |
| LABH01 | Lung cancer | 0.78 | Detected | 1.05 | Detected | KRAS_G12DC>T | III | | Yes |  |
| LAAL31 | Lung cancer | 0.98 | Detected | 1.16 | Detected | KRAS_G12CC>A  TP53_V157FC>A | Nonmetastatic with unknow staging | | Yes |  |
| LAAL98 | Lung cancer | 1.00 | Detected | 17.565 | Detected | KRAS_G12VC>A  TP53_R213*G>A | Nonmetastatic with unknow staging | | Yes |  |
| LAAM80 | Lung cancer | 1.00 | Detected | 21.33 | Detected | TP53_R248QC>T | Nonmetastatic with unknow staging | | No |  |
| LAAM90 | Lung cancer | 1.00 | Detected | 80.36 | Detected | KRAS_G12CC>A | Nonmetastatic with unknow staging | | Yes |  |
| LAAK82 | Lung cancer | 0.73 | Detected | <0.05% | Not detected | Negative | Nonmetastatic with unknow staging | | No |  |
| LAAM03 | Lung cancer | 0.92 | Detected | <0.05% | Not detected | Negative | Nonmetastatic with unknow staging | | No |  |
| LAAM12 | Lung cancer | 0.75 | Detected | 1.51 | Detected | EGFR_K745RA>G  EGFR_E746_A750delAGGAATTAAGAGAAGC>A | Nonmetastatic with unknow staging | | Yes |  |
