# Supplementary material for: Combination of Hotspot Mutations With Methylation and Fragmentomic Profiles to Enhance Multi‐Cancer Early Detection
Source: Cancer Med. 2025 Jan 3;14(1):e70575. doi: 10.1002/cam4.70575 (PMC11695824; doi:10.1002/cam4.70575)
Supplement: Supplementary file 6 — Table S5. Detection Accuracy of the combination approach. [file CAM4-14-e70575-s006.docx]

| **Table S5: Detection Accuracy of the combination approach** | | | | |
| --- | --- | --- | --- | --- |
| **Type** | **Accuracy percentage** | **lowCI** | **highCI** | **Note** |
| Breast cancer | 31.3 | 21.2 | 43.4 | Hotspot only |
| Colorectal cancer | 59.3 | 46.6 | 70.9 | Hotspot only |
| Gastric cancer | 41.9 | 30.5 | 54.3 | Hotspot only |
| Liver cancer | 96.6 | 82.8 | 99.4 | Hotspot only |
| Lung cancer | 53.7 | 38.7 | 67.9 | Hotspot only |
| Pan-cancer | 51.4 | 45.3 | 57.4 | Hotspot only |
| Healthy-control | 99.3 | 97.6 | 99.8 | Hotspot only |
| Breast cancer | 51.6 | 39.6 | 63.4 | SPOT-MAS model only |
| Colorectal cancer | 57.6 | 44.9 | 69.4 | SPOT-MAS model only |
| Gastric cancer | 62.9 | 50.5 | 73.8 | SPOT-MAS model only |
| Liver cancer | 100.0 | 88.3 | 100.0 | SPOT-MAS model only |
| Lung cancer | 80.5 | 66.0 | 89.8 | SPOT-MAS model only |
| Pan-cancer | 65.9 | 59.9 | 71.4 | SPOT-MAS model only |
| Healthy-control | 98.4 | 96.2 | 99.3 | SPOT-MAS model only |
| Breast cancer | 67.2 | 55.0 | 77.4 | Combination |
| Colorectal cancer | 81.4 | 69.6 | 89.3 | Combination |
| Gastric cancer | 74.2 | 62.1 | 89.3 | Combination |
| Liver cancer | 100.0 | 88.3 | 89.3 | Combination |
| Lung cancer | 82.9 | 68.7 | 89.3 | Combination |
| Pan-cancer | 78.4 | 73.0 | 89.3 | Combination |
| Healthy-control | 97.7 | 95.3 | 89.3 | Combination |
